# Development and validation of prognostic index based on purine metabolism genes in patients with bladder cancer

**DOI:** 10.3389/fmed.2023.1193133

**Published:** 2023-09-14

**Authors:** Zixuan Wu, Ziqing Feng, Hongyan Wei, Chuying Lin, Ke Chen

**Affiliations:** ^1^Guangzhou University of Chinese Medicine, Guangzhou, Guangdong, China; ^2^Department of Clinical Laboratory, The Sixth Affiliated Hospital, Sun Yat-sen University, Guangzhou, China; ^3^Biomedical Innovation Center, The Sixth Affiliated Hospital, Sun Yat-sen University, Guangzhou, China

**Keywords:** BLCA, PMGs, immunity, m6A and immune checkpoint, drug prediction, CNV, SNP

## Abstract

**Background:**

Bladder cancer (BLCA) is a prevalent malignancy affecting the urinary system and is associated with significant morbidity and mortality worldwide. Dysregulation of tumor metabolic pathways is closely linked to the initiation and proliferation of BLCA. Tumor cells exhibit distinct metabolic activities compared to normal cells, and the purine metabolism pathway, responsible for providing essential components for DNA and RNA synthesis, is believed to play a crucial role. However, the precise involvement of Purine Metabolism Genes (PMGs) in the defense mechanism against BLCA remains elusive.

**Methods:**

The integration of BLCA samples from the TCGA and GEO datasets facilitated the quantitative evaluation of PMGs, offering potential insights into their predictive capabilities. Leveraging the wealth of information encompassing mRNAsi, gene mutations, CNV, TMB, and clinical features within these datasets further enriched the analysis, augmenting its robustness and reliability. Through the utilization of Lasso regression, a prediction model was developed, enabling accurate prognostic assessments within the context of BLCA. Additionally, co-expression analysis shed light on the complex relationship between gene expression patterns and PMGs, unraveling their functional relevance and potential implications in BLCA.

**Results:**

PMGs exhibited increased expression levels in the high-risk cohort of BLCA patients, even in the absence of other clinical indicators, suggesting their potential as prognostic markers. GSEA revealed enrichment of immunological and tumor-related pathways specifically in the high-risk group. Furthermore, notable differences were observed in immune function and m6a gene expression between the low- and high-risk groups. Several genes, including CLDN6, CES1, SOST, SPRR2A, MYBPH, CGB5, and KRT1, were found to potentially participate in the oncogenic processes underlying BLCA. Additionally, CRTAC1 was identified as potential tumor suppressor genes. Significant discrepancies in immunological function and m6a gene expression were observed between the two risk groups, further highlighting the distinct molecular characteristics associated with different prognostic outcomes. Notably, strong correlations were observed among the prognostic model, CNVs, SNPs, and drug sensitivity profiles.

**Conclusion:**

PMGs have been implicated in the etiology and progression of bladder cancer (BLCA). Prognostic models corresponding to this malignancy aid in the accurate prediction of patient outcomes. Notably, exploring the potential therapeutic targets within the tumor microenvironment (TME) such as PMGs and immune cell infiltration holds promise for effective BLCA management, albeit necessitating further research. Moreover, the identification of a gene signature associated with purine Metabolism presents a credible and alternative approach for predicting BLCA, signifying a burgeoning avenue for targeted therapeutic investigations in the field of BLCA.

## 1. Introduction

Bladder cancer (BLCA) represents a prevalent malignancy with a substantial global burden in terms of both morbidity and mortality (1). Annually, more than 500,000 new cases of BLCA are reported worldwide, accompanied by approximately 200,000 deaths attributed to this disease (2). BLCA can be categorized into two main subtypes: muscle-invasive BLCA and non-muscle-invasive BLCA. While the non-muscle-invasive variant exhibits a favorable 5-year survival rate of 90%, a significant proportion (15–20%) of these patients undergo disease progression, leading to a marked decline in survival rate to a minimum of 60% (3). The primary treatment modalities for BLCA comprise surgical intervention and adjuvant chemotherapy (4). Nonetheless, the prognosis for patients remains bleak due to the occurrence of postoperative relapse, even following complete resection of the tumor with curative intent (5). Chemotherapy is predominantly employed for the management of muscle invasive or advanced BLCA. Nevertheless, the emergence of drug resistance in patients following chemotherapy renders them susceptible to tumor recurrence, disease progression, and ultimately, death (6). Therefore, the identification of therapeutic targets for BLCA and the molecular characterization of diagnostic biomarkers hold paramount importance for both fundamental and clinical investigations pertaining to this malignancy.

Nutrient absorption and metabolism are indispensable processes for all living organisms. Among the myriad of metabolites, purines hold immense significance as they serve as essential building blocks for DNA and RNA, making them vital for sustaining life (7). Moreover, purines are integral components of various biomolecules, including ATP, GTP, cAMP, NADH, and coenzyme A. These molecules play pivotal roles in diverse cellular processes such as energy production, cellular signaling pathways, redox metabolism, and fatty acid synthesis. Additionally, purines are implicated in immunological responses and mediate host-pathogen interactions, including tumor-host interactions (8). In mammalian cells, purine metabolism is primarily governed by two major pathways: the *de novo* synthesis pathway and the salvage pathway (9). While the salvage pathway, involving the recycling of degraded purine bases, caters to the majority of cellular purine requirements, rapidly proliferating cells and tumor cells exhibit heightened demands for purines, often met through upregulation of the *de novo* synthesis pathway. Notably, purines play a pivotal role in tumor cell replication, thus paving the way for the development of purine antimetabolites as the first-generation anticancer drugs, currently employed in the treatment of acute lymphocytic leukemia, acute myeloid leukemia, and chronic myeloid leukemia (10). These purine antimetabolites exert their therapeutic effect by inhibiting DNA synthesis and impeding cellular proliferation. Recently, the discovery of purinosomes, distinct organelles involved in purine metabolism, has shed light on their formation, tightly linked to the cell cycle (11). These intriguing findings propose a novel therapeutic strategy targeting purinosome assembly and purine metabolism, holding promise for innovative approaches in cancer therapy.

Tumor microenvironments (TME) encompass a complex milieu characterized by a constellation of unfavorable conditions, including hypoxia, heightened oxidative stress, acidic pH, and nutrient scarcity, which arise due to the uncontrolled proliferation of tumor cells coupled with inadequate angiogenesis (12). In response to these hostile TME factors, cancer cells undergo metabolic reprogramming, thereby acquiring distinct metabolic traits that confer them with a survival advantage and facilitate sustained growth, even under conditions of attenuated carcinogenic signaling. The reprogramming of energy metabolism is a hallmark feature of cancer, playing a pivotal role in driving cellular proliferation and division (13). Unlike their normal counterparts, cancer cells exhibit altered utilization patterns of glucose, lipids, and purines. Intriguingly, emerging evidence suggests that purine metabolism exerts a notable influence on oncogenesis and the metastatic cascade in cancer.

**Figure 1:**
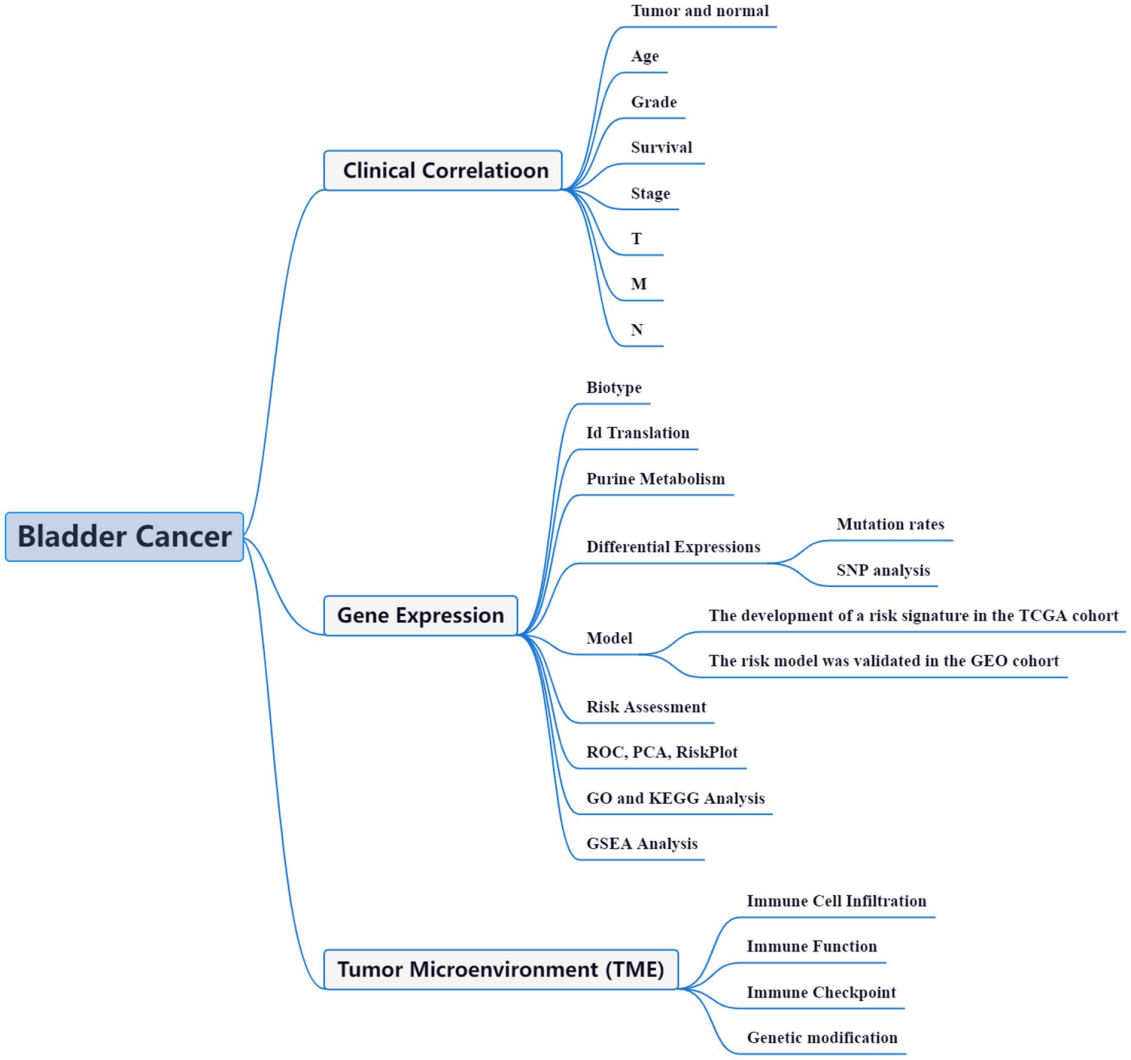
Framework.

High-throughput data analysis, aided by advanced bioinformatic tools, has become a widely adopted approach to comprehensively unravel the functional networks of genes in diverse disease models. This strategy has proven invaluable in offering crucial insights into the molecular mechanisms underlying various pathological conditions (14). Within the realm of BLCA, bioinformatics studies have also emerged as a prominent field of investigation. These studies leverage the power of computational analyses to gain a deeper understanding of BLCA at the molecular level (15–17). With a plethora of ongoing bioinformatics investigations, the intricate complexities of BLCA are being unraveled, thereby providing valuable clues for potential therapeutic interventions. The precise etiology and mechanisms underlying the aberrant gene expression and perturbed purine metabolism in BLCA remain shrouded in mystery. Unraveling the intricate relationship between purine metabolism and the synthesis of BLCA holds great potential in identifying promising biomarkers and therapeutic strategies. In this context, we present a coherent framework to investigate the intricate interplay between purine metabolism and BLCA pathogenesis (Figure 1). Through this comprehensive study, we aim to decipher the complex molecular landscape of BLCA, opening up new avenues for innovative treatment modalities and personalized medicine approaches.

## 2. Materials and methods

The research methodology employed in this study was developed based on the approach described by Zixuan Wu et al. in 2022 (18).

### 2.1. Datasets and PMGs

A total of 412 BLCA tissues and 19 normal tissues were included in this study, sourced from the esteemed repository, The Cancer Genome Atlas (TCGA) (19), as of February 26, 2023. To augment our understanding of BLCA at the molecular level, we further accessed microarray data on mRNA expression from publicly available resources, including the Gene Expression Omnibus (GEO) database. Specifically, we utilized datasets GSE13507, GSE48075, and GSE48276, alongside the corresponding platforms GPL6102, GPL6947, and GPL14951. The GEO database proved instrumental in maintaining the expression patterns of an additional 402 BLCA cases, as outlined in Supplementary Table S1A. Subsequently, a curated set of 175 PMGs was obtained from the Molecular Signatures Database (MSigDB), as presented in Supplementary Table S1B.

### 2.2. Identification of DEGs associated with purine metabolism and examination of mutation rates in DEGs

To obtain accurate mRNA data, transcription data were processed and organized using Perl scripting. The IDs were then converted into corresponding gene names. By comparing the data between the BLCA sample group and the normal sample group, significant changes in the expression of PMGs were observed. Genes with a FDR below 0.05 and a |log2FC| greater than or equal to 1 were considered DEGs. The relevance of these DEGs was further investigated.

The variant frequencies of the DEGs were evaluated using the Cbioportal platform. Correlation analysis between the expression of DEGs in the prognostic model and CNV was conducted using the Spearman method (*p* < 0.05) and visualized using the Corrplot R package. Furthermore, the correlation between the expression of DEGs in the prognostic model and drug sensitivity was assessed using the Pearman method (*p* < 0.05) based on the corresponding data from CellMiner.

### 2.3. Tumor categorization using the DEGs

To categorize tumors based on the identified DEGs, we conducted cluster analysis using the Limma and ConsensusClusterPlus packages. This analysis resulted in the classification of prognosis-related PMGs into two distinct clusters: cluster 1 and cluster 2. To assess the relationship between PMGs and patient survival, we employed Survminer, which allowed us to investigate PMGs survivorship and evaluate their predictive value in terms of patient outcomes.

Furthermore, the Limma package was employed to identify specific gene alterations among different subtypes and tissue types. This analysis facilitated the identification of genes that exhibited significant changes in expression levels, providing valuable insights into the molecular distinctions between various tumor subtypes and tissue types.

### 2.4. The establishment of a predictive signature for PMGs

In order to develop a prognostic model for PMGs, we employed the glmnet and survival packages. The predictive signature for PMGs was constructed using Lasso-penalized Cox regression and Univariate Cox regression analysis. The risk score for each bladder cancer (BLCA) patient was determined based on the formula: (Coefficient DEGs1 × expression of DEGs1) + (Coefficient DEGs2 × expression of DEGs2) + … + (Coefficient DEGsn × expression DEGsn). This risk score was then used to stratify patients into two subgroups: low-risk (< median number) and high-risk (≥ median number).

Lasso regression was performed to identify the low-risk and high-risk groups, and the results were visualized through appropriate plots. Subsequently, the confidence interval and risk ratio were calculated, and a forest diagram was generated using the pheatmap package. Survival curves were plotted to analyze the differences between the high-risk and low-risk groups.

To assess the accuracy of the prognostic model in predicting survival outcomes in BLCA, the timeROC package was utilized to generate a receiver-operating characteristics (ROC) curve for comparison. The risk score was evaluated in relation to the chance curve and examined for its association with PMGs’ risk and survival status. Additionally, an independent prognostic study was conducted to confirm the model’s reliability across different clinical factors. The relationship between clinical characteristics and the risk prediction model, as well as the relationship between the two PMGs in patients, were analyzed. The analysis of risk and clinical relationships was comprehensively performed.

Moreover, Principal Component Analysis (PCA) and T-distributed Neighbor Embedding (T-SNE) were employed using the Rtsne and ggplot2 packages to investigate the potential of the prognostic model to accurately categorize patients into two risk groups. By integrating the predictive signals, a representation was developed to predict the 1-, 3-, and 5-year overall survival (OS) of BLCA patients.

### 2.5. Functional enrichment of PMGs with differential expression

To gain insights into the biological functions and pathways associated with the differentially expressed PMGs, we performed GO and KEGG analyses. Using R, we explored the BP, MF, and CC regulated by the differentially expressed PMGs.

### 2.6. The predicted nomogram and GSEA enrichment analysis

To identify relevant functions and pathway alterations across a range of samples, we employed GSEA. The accompanying scores and diagrams were used to assess the dynamic activities and pathways within the various risk subcategories. Each sample was labeled as either ‘H’ or ‘L’ based on the analysis results.

### 2.7. Comparison of immune activity levels in different subgroups

We utilized ssGSEA to evaluate the enriching values of immune cells and activities in different subgroups. Additionally, we examined the relationship between PMGs, immune checkpoints, and mRNA chemical modifications (such as m6A, m1A, M7G, and m5C). Furthermore, regulators of m6A, m1A, M7G, and m5C were identified to further investigate their connection with immune activity levels (20) (Supplementary Table S2).

## 3. Results

### 3.1. Differentially expressed PMGs

**Figure 2:**
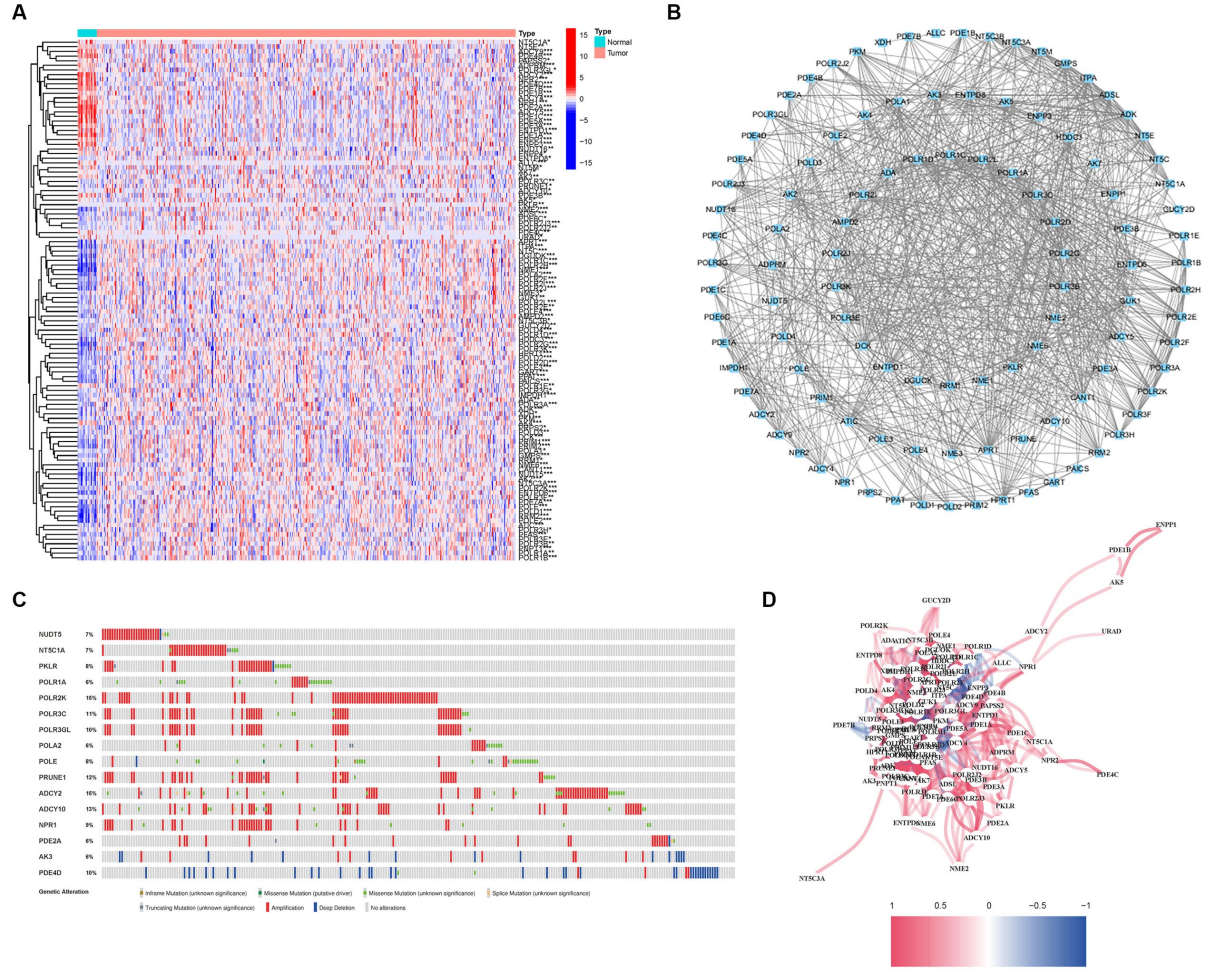
Expressions of the 112 PMGs and their interactions. **(A)** A PPI network illustrating the interactions of PMG. **(B)** The Purine Metabolism gene correlation network. **(C)** Mutations in PMGs. 16 genes over a 5% mutation rate, with POLR2K and ADCY2 being the most often modified (15%). **(D)** The correlation network of the genes participating in autophagy (red line: positive correlation; blue line: negative correlation. The depth of the colors reflects the strength of the relevance).

A comprehensive analysis was conducted to explore the relationship between purine metabolism and bladder cancer. Through differential gene expression analysis, a total of 112 genes were identified as being associated with purine metabolism, comprising 80 upregulated genes and 32 downregulated genes (Supplementary Table S2; Figure 2A). To assess the potential interactions among these PMGs, a PPI network was constructed (Figure 2B). Notably, by applying a more stringent interaction threshold of 0.7, several hub genes were identified, including GMPS, ENTPD1, APRT, ENTPD8, ADSL, GUK1, and ITPA (Supplementary Table S3). It is worth mentioning that these hub genes encompassed all the differentially expressed genes observed in both normal and cancerous bladder tissues, thereby highlighting their potential as prognostic markers for BLCA. The correlation network of all PMGs is visually presented in Figure 2C.

Given the clinical significance of these PMGs, further investigations were conducted to explore the genetic aberrations within these genes. Specifically, the focus was on identifying the types of mutations that occurred. The analysis revealed that the most prevalent types of mutations were truncating variants and missense variants (Figure 2D). Among the analyzed genes, a total of 16 genes exhibited a mutation rate exceeding 5%, with POLR2K and ADCY2 being the most frequently altered genes, occurring in 15% of the cases. These findings shed light on the genetic landscape of bladder cancer, emphasizing the importance of studying the genetic anomalies within these PMGs due to their clinical implications.

### 3.2. Alterations of purine metabolism regulatory genes are associated with clinicopathological and molecular characteristics

**Figure 3:**
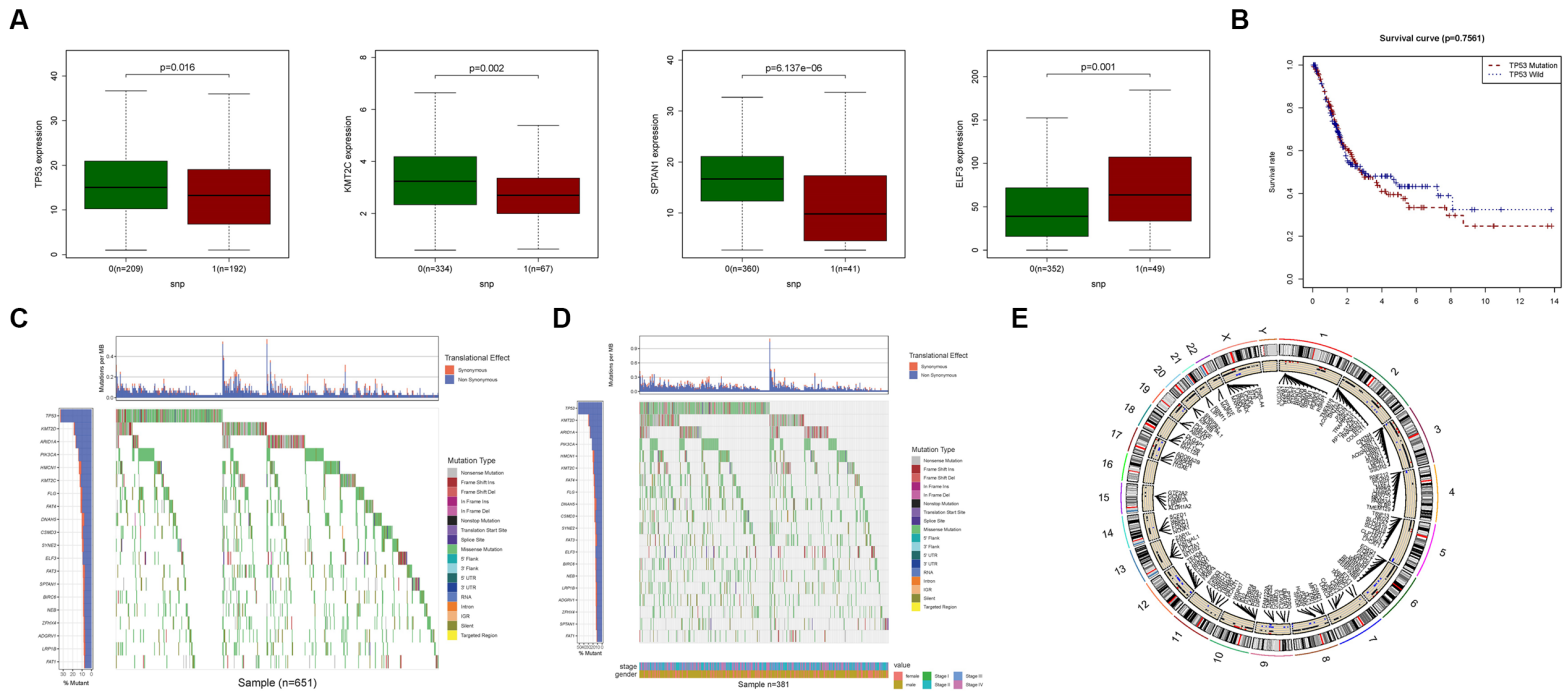
CNV, SNP and mutation analysis. **(A)** Correlation analysis in prognostic signatures and SNP. **(B)** The survival analysis of TP53. **(C,D)** The mutation distribution of genes in prognostic signatures. **(E)** CNV analysis.

We conducted an investigation into the association between alterations in regulatory genes involved in PyM and the clinicopathological parameters of patients. By performing correlation analyses between the expression levels of DEGs in the prognostic model and SNPs, we identified four SNP-driven DEGs, namely TP53, ELF3, KMT2C, and SPTAN1 (Figure 3A). Notably, the expression of TP53 was significantly higher in the group with single mutations compared to the group without mutations, indicating that SNPs in BLCA may lead to dysregulation of crucial genes (*p* < 0.05) (Figure 3B). To visually represent the gene mutation status, a waterfall plot was utilized. The average mutation frequency of DEGs within the prognostic model ranged from 12% to 50% (Figures 3C,D), suggesting a potential relationship between BLCA mutations and the dysregulation of key genes. Additionally, we examined the correlation between the expression levels of DEGs in the prognostic model and CNVs, revealing several CNV-driven DEGs (Figure 3E).

Furthermore, our medication prediction model highlighted certain genes that exhibited substantial differences (Supplementary Figure S1). Additionally, an analysis of the relationship between the expression levels of DEGs in the prognostic model and drug sensitivity revealed strong associations between multiple genes and drug sensitivity. Notably, KRT1 demonstrated a significant relationship with Nelarabine, Fluphenazine, Dexamethasone Decadron, Hydroxyurea, and Fludarabine, indicating potential medication pathways (Supplementary Figure S2). These findings provide valuable insights into the potential implications of DEGs and their association with drug sensitivity, thereby suggesting promising avenues for medication strategies in the context of BLCA.

### 3.3. Tumor categorization based on differentially expressed genes

**Figure 4:**
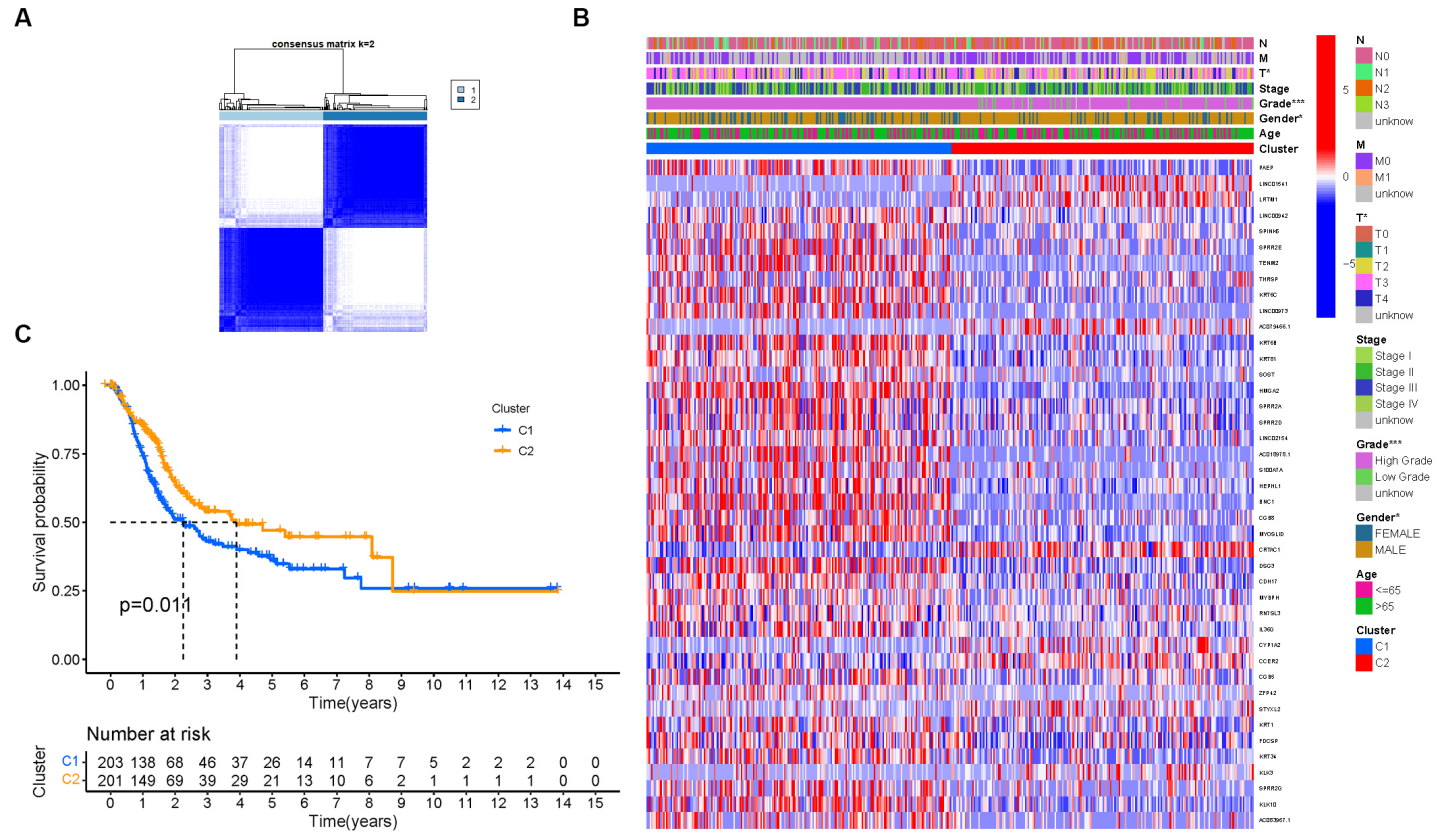
Tumor categorization based on DEGs associated with purine metabolism. **(A)** The consensus clustering matrix (*k* = 2) was used to divide 414 BLCA patients into two groups. Heatmap **(B)**. The heatmap and clinicopathologic features of the two clusters identified by these DEGs (T, Grade, and Stage indicate the degree of tumor differentiation). *p* values were showed as:*p < 0.05; **p < 0.01; ***p < 0.001. **(C)** Kaplan–Meier OS curves for the two clusters.

To investigate the relationship between the expression of PMGs and BLCA, a consensus clustering analysis was performed on the entire cohort of 414 BLCA patients from the TCGA dataset. By setting the clustering variable (k) to 2, we observed the strongest intragroup correlation and the weakest intergroup correlation, indicating that the 414 BLCA patients could be categorized into two distinct groups based on the expression patterns of their PMGs (Figure 4A). The heatmap visualization provides a comprehensive display of gene expression profiles along with corresponding clinical features (Figure 4B; Supplementary Table S4). Furthermore, a survival analysis was conducted to explore the prognostic potential of PMG subtypes, revealing that patients belonging to cluster 2 exhibited a significantly higher survival rate compared to those in cluster 1 (*p* = 0.011) (Figure 4C).

These findings highlight the significance of PMGs in tumor categorization and underscore their potential as prognostic markers for BLCA patients. The identified subtypes based on PMG expression patterns provide valuable insights into the heterogeneity of BLCA and may contribute to personalized therapeutic approaches and improved patient outcomes.

### 3.4. Development of a prognostic gene model in the TCGA cohort

**Figure 5:**
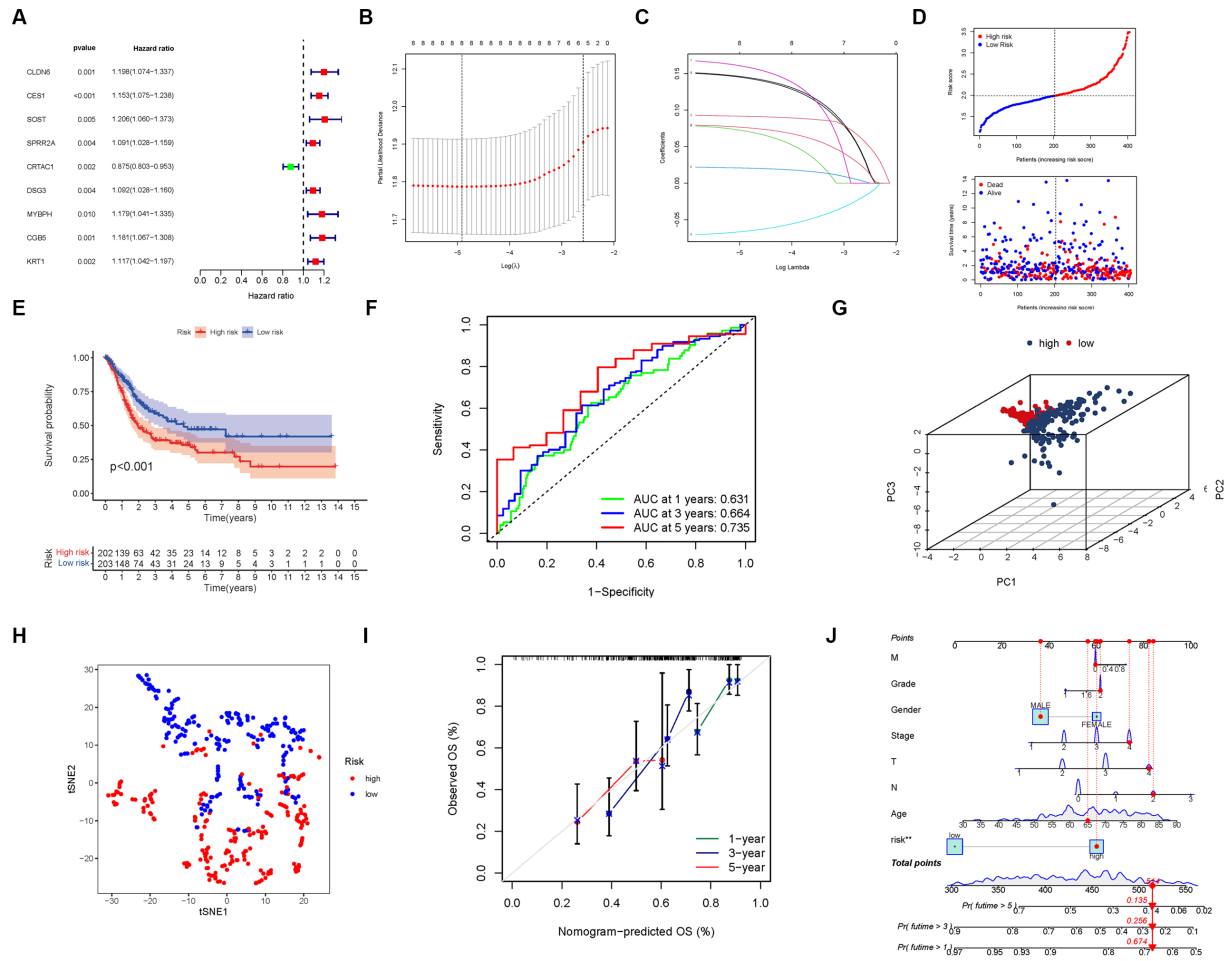
The development of a risk signature in the TCGA cohort. **(A)** A Univariate Cox regression analysis of OS for each purine metabolism-related gene, with *p* < 0.05 for 9 genes. **(B)** Regression of the 9 OS-related genes using LASSO. **(C)** Cross-validation is used in the LASSO regression to fine-tune parameter selection. **(D)** The patient’s chance of survival. **(E)** Kaplan–Meier curves for patients in the high- and low-risk groups’ OS. **(F)** The AUC for predicting the 1-, 3-, and 5-year survival rates of BLCA. **(G)** A PCA plot based on the risk score for BLCAs. **(H)** A t-SNE plot based on the risk score for BLCAs. **(I,J)** Nomogram.

Through an extensive univariate Cox analysis, we identified nine significant PMGs that emerged as independent prognostic indicators for BLCA (Figure 5A). These PMGs, namely CLDN6, CES1, SOST, SPRR2A, CRTAC1, DSG3, MYBPH, CGB5, and KRT1, held considerable promise in predicting the prognosis of BLCA patients. To construct a robust gene signature, we employed the LASSO regression analysis and Cox regression analysis, determining the optimal value for gene selection (Figures 5B,C). The resulting gene signature was associated with the risk scores of individual patients, revealing an inverse correlation with BLCA survival. Notably, the majority of the newly discovered PMGs exhibited a negative correlation with the risk model, necessitating further investigations into their functional roles (Figure 5D).

We found that the presence of high-risk PMG signatures was significantly associated with a lower likelihood of survival (*p* < 0.001, Figure 5E). The AUC values for the unique PMG signature in predicting the 1-, 3-, and 5-year survival rates were 0.631, 0.664, and 0.735, respectively, indicating its predictive capacity (Figure 5F). Based on PCA and t-SNE results, patients were successfully stratified into two distinct groups, further validating the robustness of the gene signature (Figures 5G,H).

To enhance the clinical applicability of our findings, we developed a hybrid nomogram that integrated both clinicopathological characteristics from the TCGA dataset and the prognostic signature based on the selected PMGs. This nomogram demonstrated stability and accuracy, highlighting its potential as a valuable tool for guiding the therapy of BLCA patients (Figures 5I,J).

### 3.5. External validation of the risk signature

**Figure 6:**
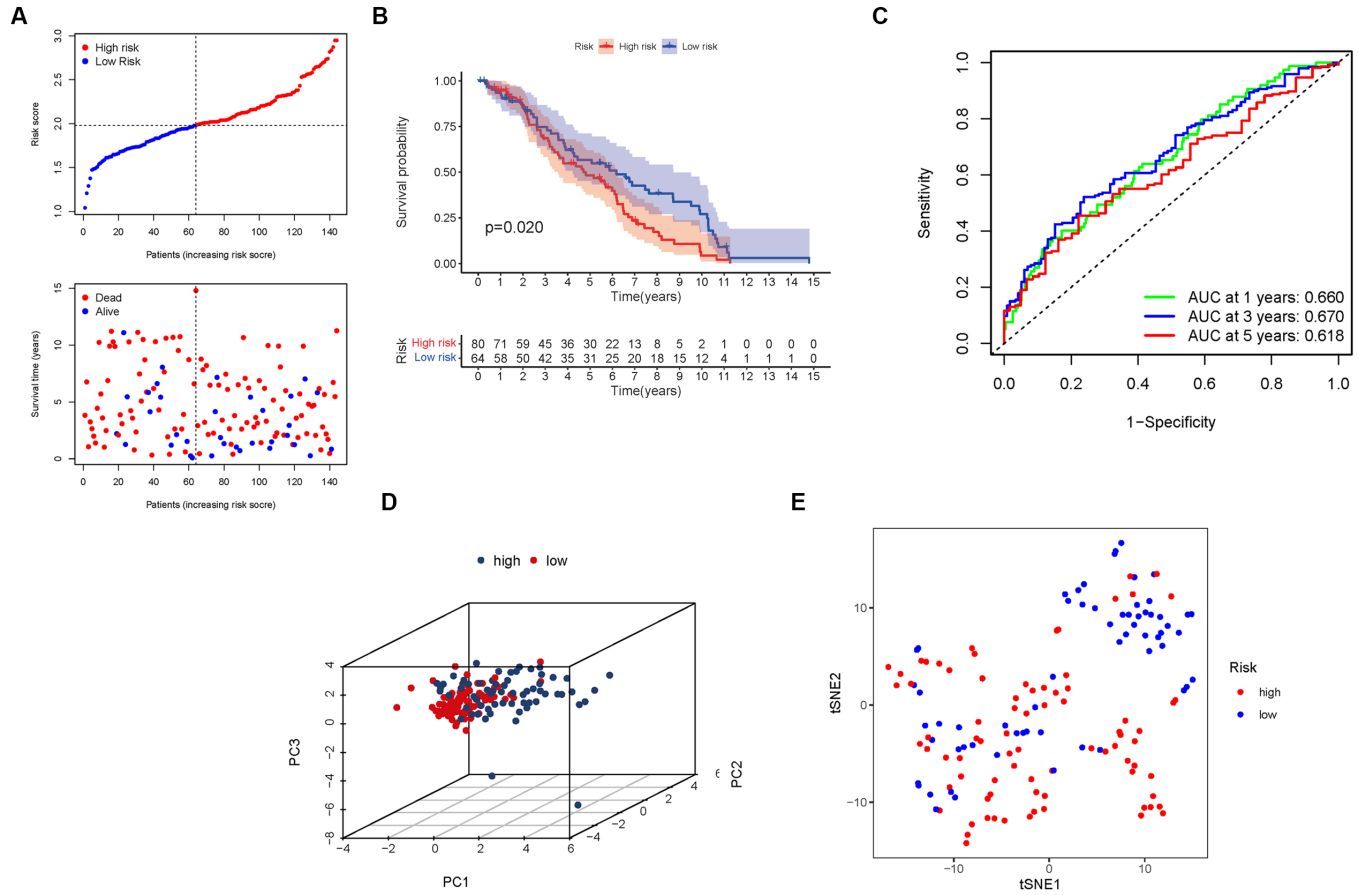
The risk model was validated in the GEO cohort. **(A)** Each patient’s chance of survival. **(B)** Kaplan–Meier curves for patients in the high- and low-risk groups’ overall survival. **(C)** The AUC for predicting the 1-, 3-, and 5-year survival rates of BLCA. **(D)** A PCA plot based on the risk score for BLCA. **(E)** A t-SNE plot based on the risk score for BLCA.

To validate the robustness and generalizability of our risk signature, we utilized an independent cohort from the GEO consisting of 402 BLCA patients. Consistent with the findings from the TCGA dataset, we observed an inverse relationship between the risk score and BLCA survival in the validation group. Similarly, the majority of the newly identified PMGs in this study displayed a negative correlation with the risk model (Figure 6A).

Furthermore, the presence of high-risk PMG signatures was significantly associated with a diminished chance of survival (*p* = 0.020), as demonstrated by Kaplan–Meier analysis (Figure 6B). The AUC values for the unique PMG signature in predicting the 1-, 3-, and 5-year survival rates were 0.660, 0.670, and 0.618, respectively (Figure 6C). It is worth noting that the relatively lower AUC values could be attributed to the high mortality rate observed within five years in the majority of BLCA patients. Nonetheless, the results of PCA and t-SNE indicated that patients with varying risk levels were effectively stratified into two distinct groups (Figures 6D,E).

These external validation results further consolidate the prognostic value and clinical relevance of our risk signature. The negative association between the risk score and BLCA survival was consistently observed in the GEO cohort. The AUC values indicate the predictive capacity of the PMG signature in the validation group, albeit considering the challenging nature of long-term survival prediction in BLCA. The successful stratification of patients into distinct risk groups based on the PCA and t-SNE results confirms the robustness and utility of our risk signature across different datasets.

### 3.6. Independent prognostic value of the risk model

**Figure 7:**
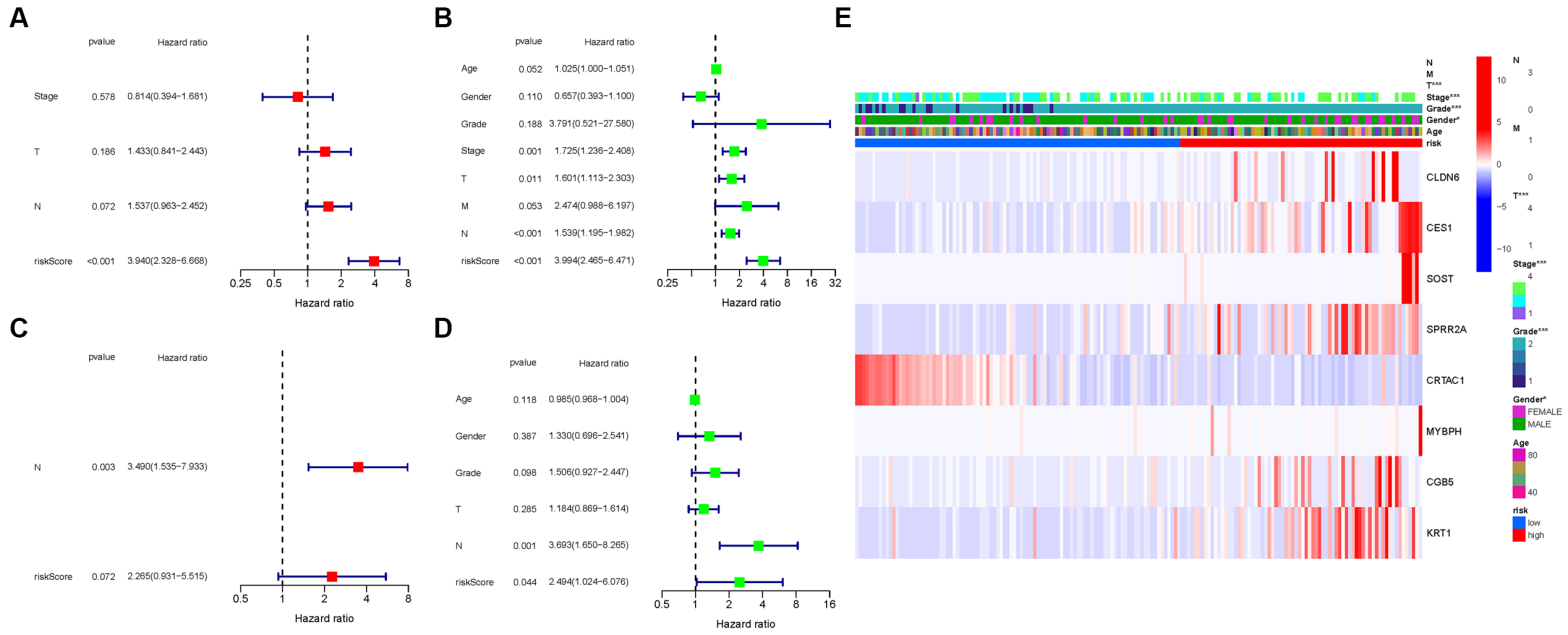
Cox regression analysis, both univariate and multivariate. **(A)** TCGA cohort multivariate analysis. **(B)** TCGA cohort univariate analysis. **(C)** GEO cohort multivariate analysis. **(D)** GEO cohort univariate analysis. **(E)** Heatmap (green: low expression; red: high expression) illustrating the relationships between clinicopathologic characteristics and risk groups (*p < 0.05; **p < 0.01; ***p < 0.001).

Cox regression analysis was performed to evaluate the independent prognostic value of the PMGs signature in both the TCGA and GEO cohorts. In the TCGA cohort, the PMGs signature exhibited a strong independent predictive value for the OS of BLCA patients (HR: 3.940, 95% CI: 2.328–6.668) (Figures 7A,B). Similarly, in the GEO cohort, the N stage emerged as a predominantly independent prognostic factor (HR: 3.490, 95% CI: 1.535–7.933) (Figures 7C,D). Furthermore, a heatmap displaying the clinical features of the TCGA cohort was generated, providing a comprehensive overview of the various parameters assessed (Figure 7E; Supplementary Tables S5, S6).

These results underscore the robustness and independent prognostic value of the PMGs signature in predicting the OS of BLCA patients. The PMGs signature consistently demonstrated its significance across both the TCGA and GEO cohorts. The identification of the N stage as an independent prognostic factor further highlights the multifactorial nature of BLCA prognosis. The heatmap visualization provides a comprehensive representation of the clinical characteristics, emphasizing the complexity of BLCA and the potential implications of the identified prognostic factors.

### 3.7. Enrichment analysis of PMGs

To gain deeper insights into the biological processes associated with the identified genes, we conducted a comprehensive GO enrichment analysis. This analysis revealed 378 core target genes that were significantly enriched in various functional categories, including BP, MF, and CC. In terms of MF, the enriched GO terms mainly involved guanyl nucleotide binding (GO:0019001), guanyl ribonucleotide binding (GO:0032561), and nucleoside binding (GO:0001882). These findings suggest the involvement of these genes in important molecular interactions and nucleotide-related processes. At the CC level, the enriched GO terms included the transferase complex, transferring phosphorus-containing groups (GO:0061695), nuclear chromosome (GO:0000228), and protein-DNA complex (GO:0032993). These results imply the presence of these genes in specific cellular compartments and their potential roles in processes related to protein-DNA interactions and chromosomal organization. Regarding BP, the enriched GO terms encompassed dephosphorylation (GO:0016311), RNA splicing (GO:0008380), and anatomical structure homeostasis (GO:0060249). These findings suggest the potential involvement of the identified genes in regulating phosphorylation events, RNA processing, and maintaining the homeostasis of anatomical structures.

**Figure 8:**
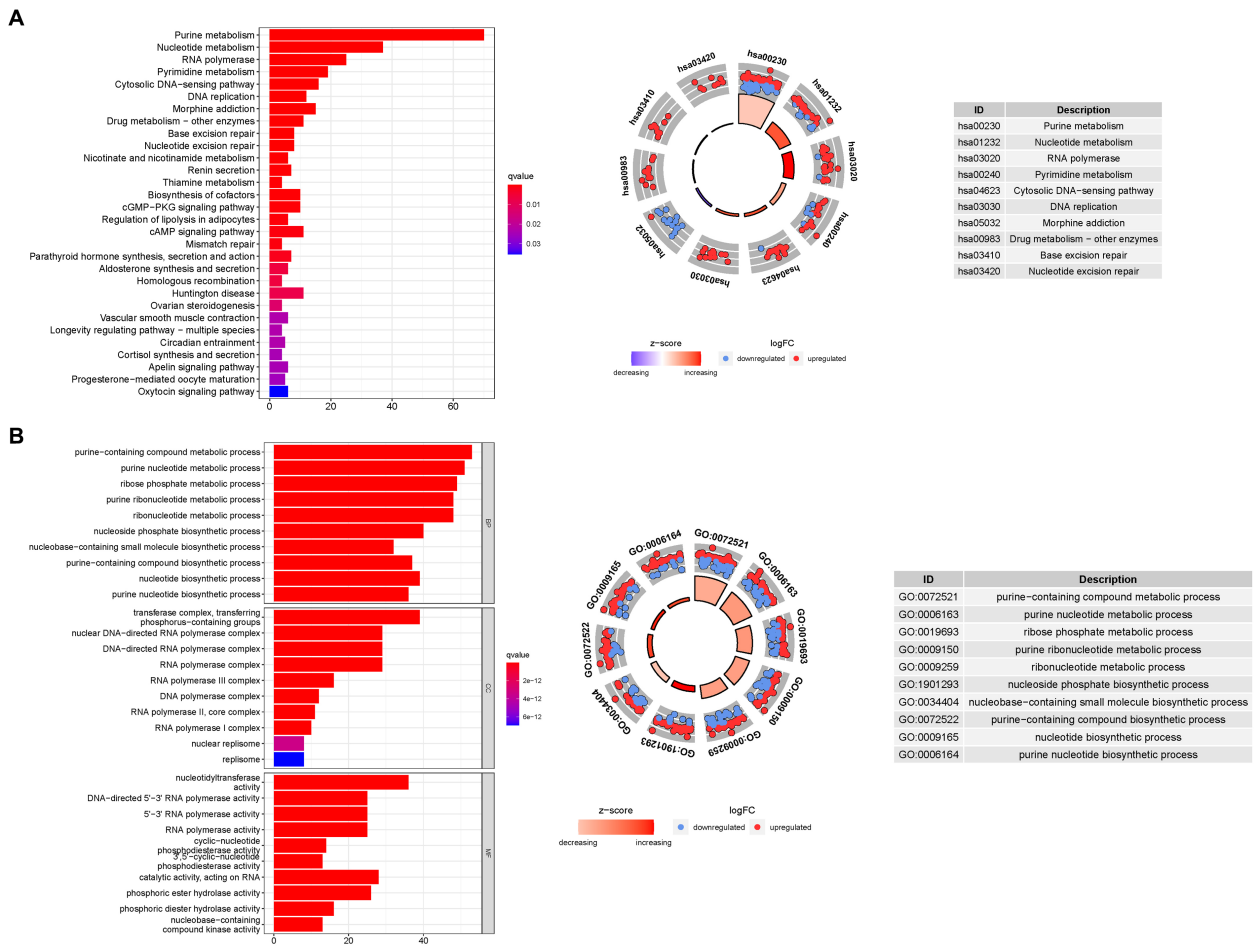
For PMGs, GO, and KEGG analyses were performed. **(A)** The GO circle illustrates the scatter map of the selected gene’s logFC. **(B)** The KEGG circle illustrates the scatter map of the logFC of the indicated gene. The greater the Z-score value, the greater the expression of the enriched pathway.

To further explore the functional pathways associated with the identified genes, we performed KEGG enrichment analysis. The overexpressed genes were predominantly involved in Huntington’s disease (hsa05016), the cAMP signaling pathway (hsa04024), and the cGMP-PKG signaling pathway (hsa04022). These findings provide valuable insights into the potential molecular mechanisms underlying bladder cancer and highlight specific pathways that may be dysregulated in the disease (Figure 8).

### 3.8. Analyses of gene set enrichment

**Figure 9:**
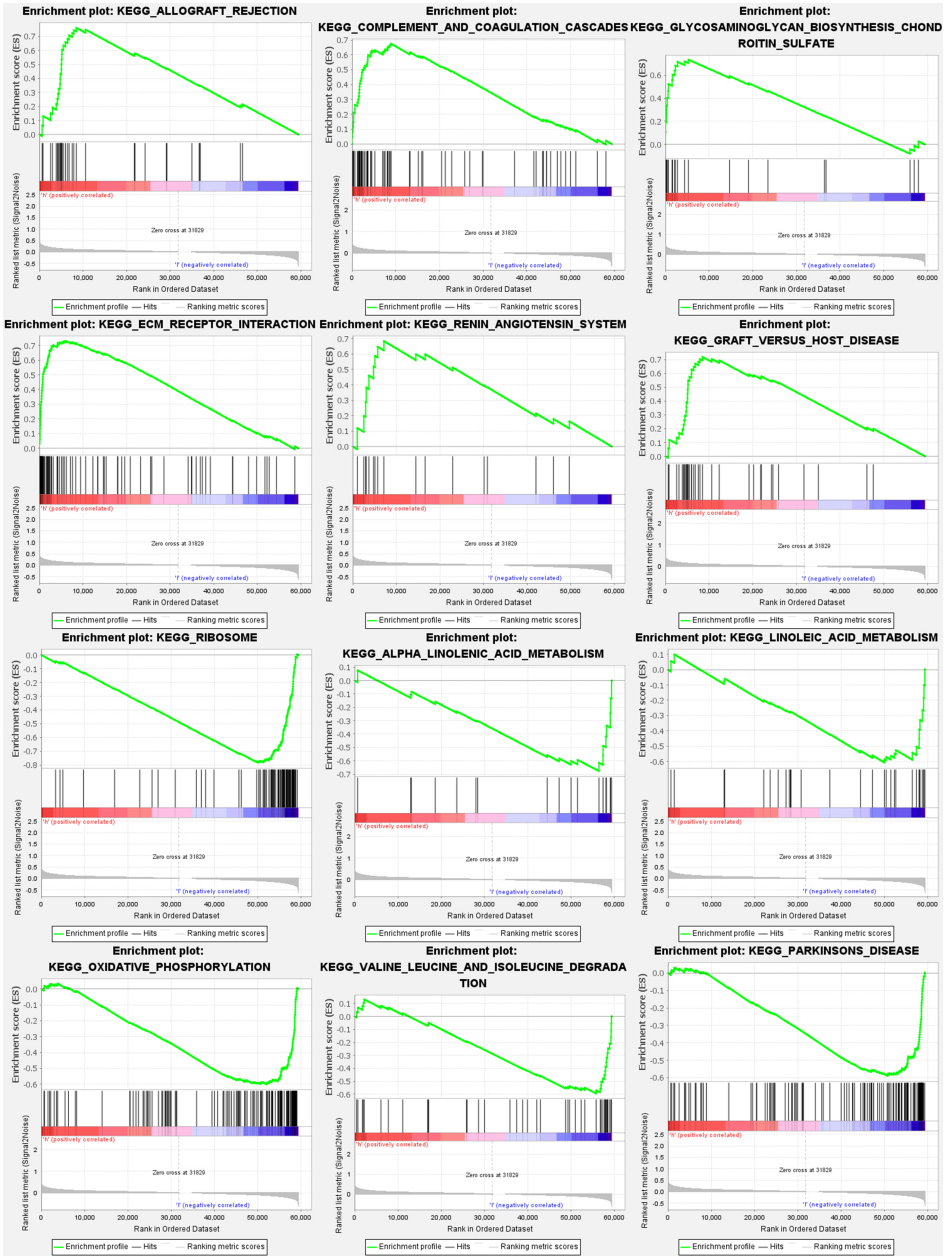
PMGs gene set enrichment studies. The top six enriched functions or pathways of each cluster were provided to illustrate the distinction between related activities or pathways in various samples. FDR *q*-value and FWER value of *p* were both <0.05.

The GSEA revealed that the prognostic signatures of most PMGs were associated with various immunological and tumor-related pathways. These pathways included allograft rejection, glycosaminoglycan biosynthesis chondroitin sulfate, ecm receptor interaction, and graft versus host disease. Each cluster exhibited the top 6 enriched functions or pathways (Figure 9; Table 1). Among them, the “nod-like receptor signaling pathway” showed the highest enrichment (Supplementary Tables S9A,B).

**Table 1:** The top six enriched functions or pathways.

Name	ES	NES	NOM *p*-val	FDR *q*-val
Allograft rejection	0.761138	1.7877699	0.021276595	0.03481406
Glycosaminoglycan biosynthesis chondroitin sulfate	0.7352404	2.0292728	0	0.006975394
Ecm receptor interaction	0.7297853	2.2827163	0	0
Graft versus host disease	0.71909297	1.6616324	0.054	0.061480176
Renin angiotensin system	0.684217	1.8742491	0.004219409	0.02193081
Complement and coagulation cascades	0.67470324	2.1027026	0.002040816	0.002401257

### 3.9. Comparison of immune activity levels in different subgroups

**Figure 10:**
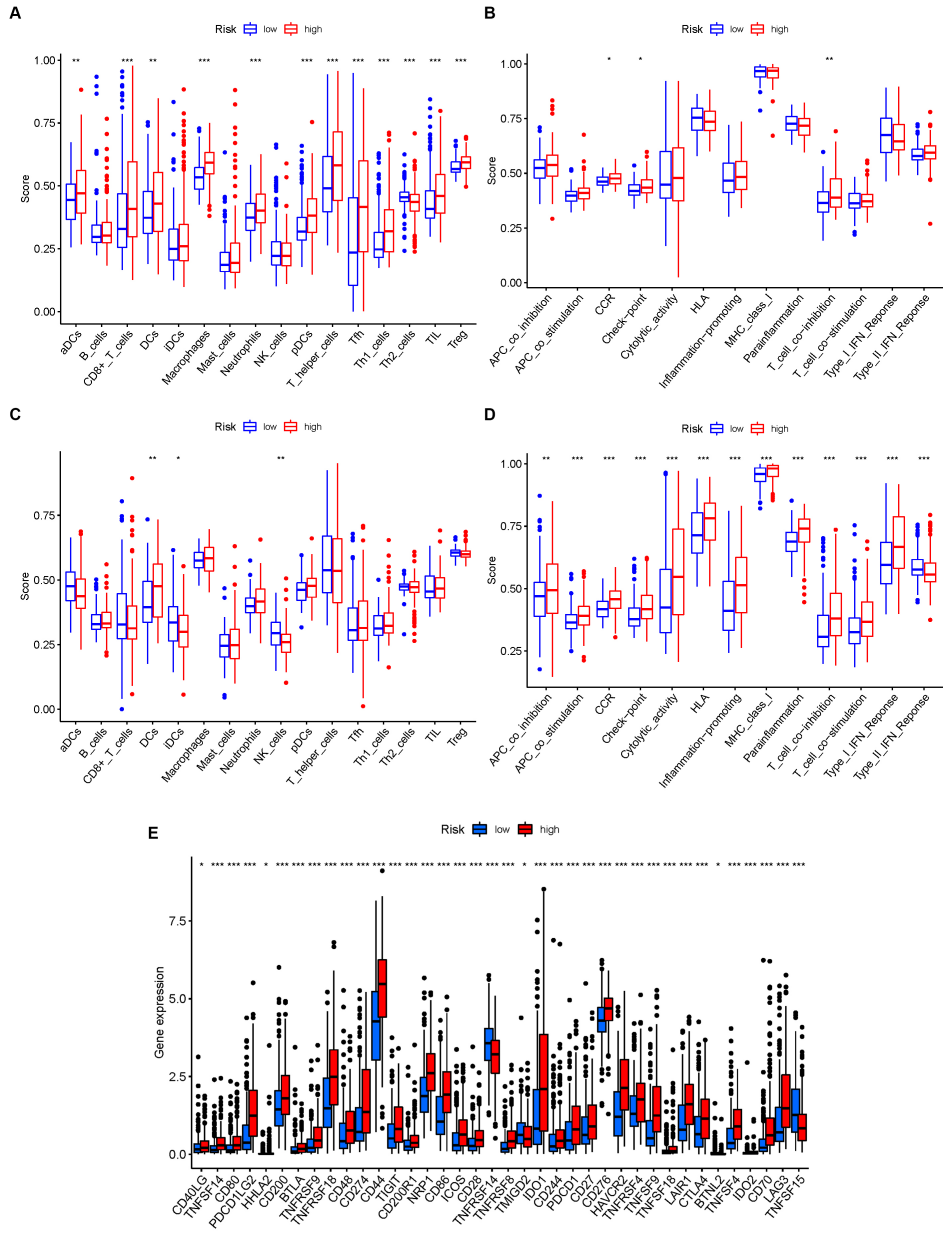
The ssGSEA scores are compared. **(A,B)** Comparison of the enrichment scores of 16 kinds of immune cells and 13 immune-related pathways in the TCGA cohort between the low-risk (green box) and high-risk (red box) groups. **(C,D)** In the GEO cohort, tumor immunity was compared between the low-risk (blue box) and high-risk (red box) groups. *p* values were shown as follows: ns not significant; *p < 0.05; **p < 0.01; ***p < 0.001. **(E)** Immune checkpoint.

To investigate the immune landscape in different risk subgroups, we compared the enrichment scores of 16 immune cell types and the activity of 13 immune-related functions between the low-risk and high-risk groups in both the TCGA and GEO cohorts using single-sample gene set enrichment analysis (ssGSEA). In the low-risk group, we observed significantly higher infiltration levels of various immune cell types, including activated dendritic cells (aDCs), CD8+ T cells, dendritic cells (DCs), macrophages, neutrophils, plasmacytoid dendritic cells (pDCs), T helper cells, T follicular helper cells (Tfh), T helper 1 cells (Th1), tumor-infiltrating lymphocytes (TILs), and regulatory T cells (Treg) (Figure 10A). These findings suggest a more favorable immune microenvironment in the low-risk subgroup, characterized by enhanced antitumor immune responses and immune cell infiltration.

Conversely, the high-risk group exhibited higher levels of immune-related activities associated with immune suppression and immune evasion, including APC co-inhibition, APC co-stimulation, chemokine receptor signaling (CCR), immune checkpoint signaling, cytolytic activity, and major histocompatibility complex class I (HLA) expression (Figure 10B). These results indicate a more immunosuppressive and immune evasive phenotype in the high-risk subgroup. Consistent conclusions were observed in the independent GEO cohort, further validating the differences in immune activity levels between risk subgroups (Figures 10C,D). Considering the importance of immune checkpoint inhibitors in cancer immunotherapy, we explored the differential expression of immune checkpoint genes between the low-risk and high-risk groups. We found significant alterations in the expression of immune checkpoint genes, including TNFSF14, CD80, PDCD1LG2, CD200, BTLA, TNFRSF9, TNFRSF18, and others (Figure 10E). These findings suggest potential differences in immune checkpoint regulation and responsiveness to immunotherapeutic interventions between the two subgroups.

**Figure 11:**
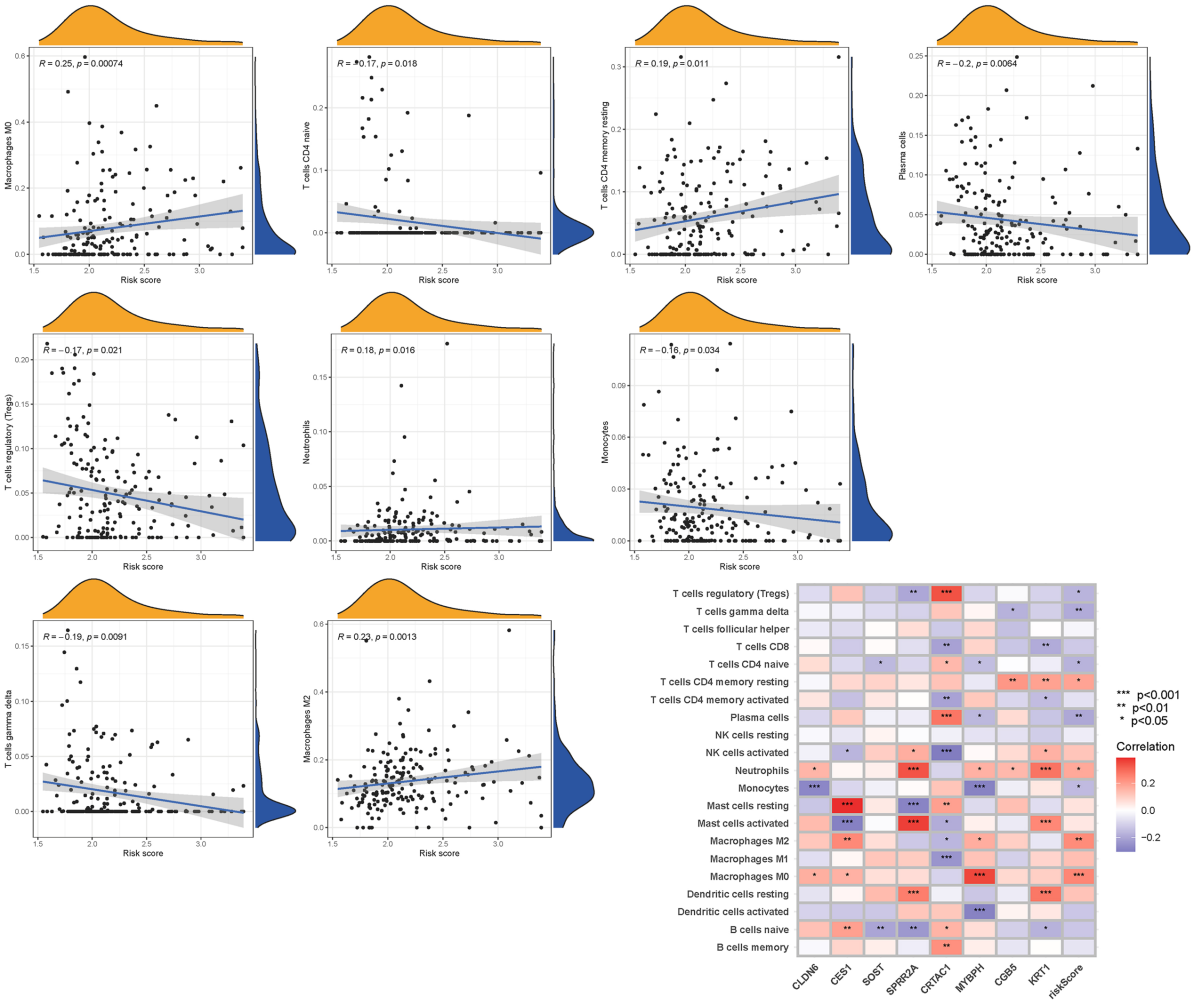
The CIBERSORT scores are validated.

To further validate the infiltration of these immune cell types, we utilized the CIBERSORT algorithm to assess their abundance in bladder cancer. Consistent with our previous findings, we observed significant differences in the immunoinfiltration levels of specific immune cell types, such as macrophages M0, macrophages M2, monocytes, and neutrophils, between the low-risk and high-risk subgroups (Figure 11). These results provide additional evidence for the distinct immune profiles associated with different risk categories in bladder cancer.

Overall, our findings highlight the contrasting immune landscapes in low-risk and high-risk subgroups, with the low-risk group exhibiting increased infiltration of effector immune cells and the high-risk group showing signs of immune suppression and immune evasion. These findings have important implications for the development of immunotherapeutic strategies and highlight the potential of immune-based interventions in bladder cancer treatment.

### 3.10. mRNA chemical modifications

**Figure 12:**
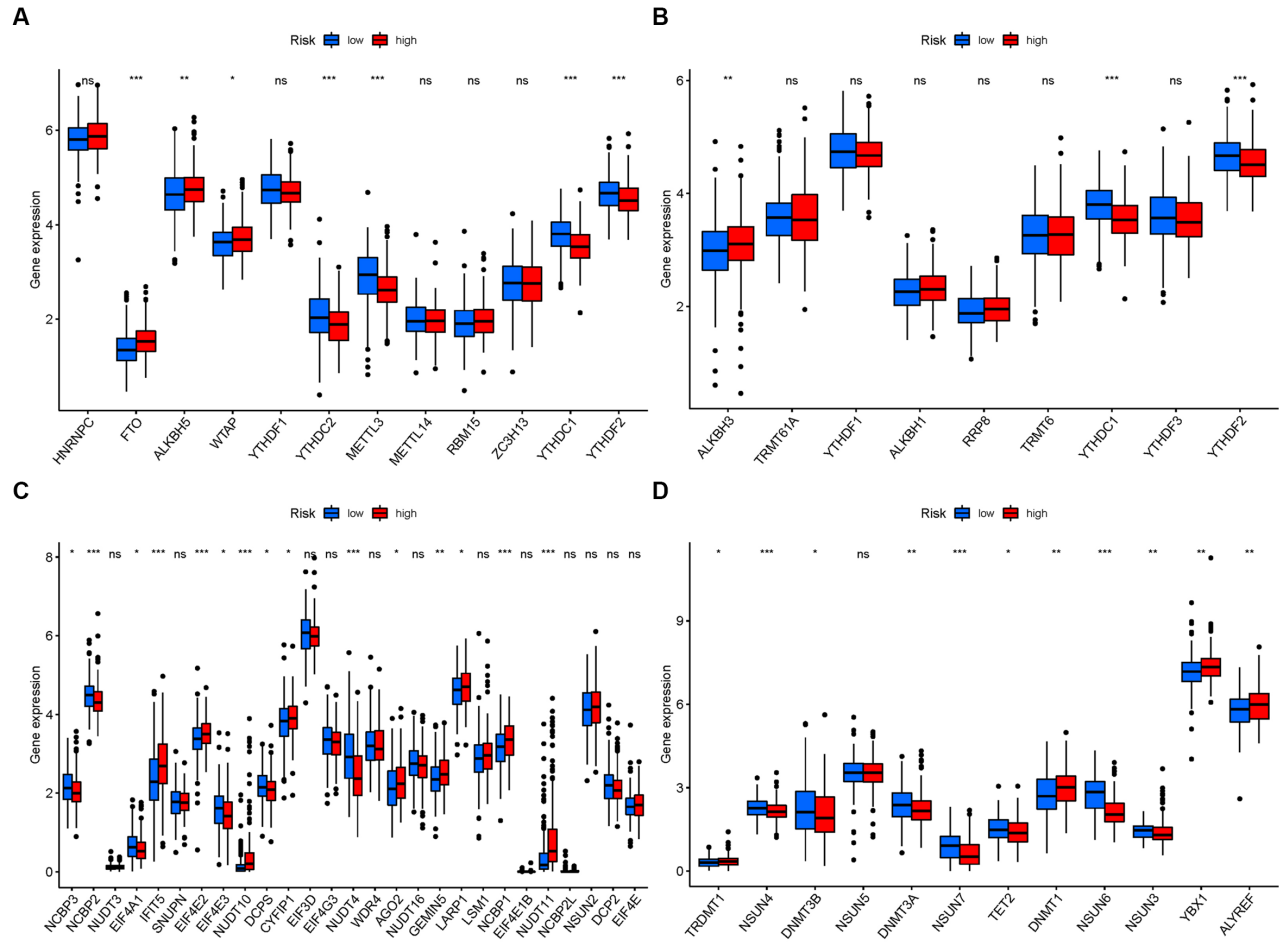
mRNA chemical modifications. **(A)** m6A. **(B)** M1A. **(C)** M7G. **(D)** M5C.

In terms of m6a modifications, significant differences in the expression of PMGs were observed between the two risk groups. *FTO, ALKBH5*, and *WTAP* showed higher significance in the high-risk group, while *YTHDC2, METTL3, YTHDC1*, and *YTHDF2* exhibited higher significance in the low-risk group (Figure 12A). Regarding m1A modifications, *ALKBH3* displayed significantly higher expression in the high-risk group compared to the low-risk group (Figure 12B). For M7G modifications, several genes including *IFIT5, EIF4E2, CYFIP1, AGO2, GEMIN5, NCBP1*, and *NUDT11* showed significantly higher expression in the high-risk group (Figure 12C). In the case of m5C modifications, *TRDMT1, DNMT1, YBX1, and ALYREF* exhibited significantly higher expression in the high-risk group (Figure 12D).

## 4. Discussion

BLCA is a prevalent malignant tumor affecting the urinary system (21). In China, BLCA is the most common genitourinary malignancy, ranking second only to prostate cancer in the United States (22). The incidence of BLCA tends to increase with age, with the highest prevalence observed among individuals aged between 50 and 70 years (23). Radical surgery represents the cornerstone of treatment for BLCA. However, due to the aggressive nature of the disease, postoperative complications such as anastomotic fistula, intestinal fistula, urinary tract infection, and urethral stricture frequently arise, leading to unfavorable prognoses and reduced survival rates (24). In recent years, various risk markers have been identified for different types of malignancies. Nonetheless, the application of these markers in clinical practice has remained largely theoretical, as rigorous evaluations and extensive replication studies are often lacking (25). Therefore, it is crucial to uncover reliable prognostic indicators for BLCA that can effectively identify individuals at high risk of disease progression. By doing so, appropriate interventions and treatment strategies can be implemented to improve patient outcomes.

Purine, a heterocyclic bicyclic aromatic molecule, plays a crucial role in various metabolic processes and cell signaling. One notable product of purine metabolism is uric acid, which tends to accumulate in the peripheral circulation when there is an increase in purine metabolism. During the process of tumor initiation and progression, several abnormalities in purine nucleotide metabolism occur. Numerous enzymes involved in purine nucleotide synthesis and degradation have been linked to tumor cell proliferation and resistance to treatment. Additionally, an imbalance in the antioxidant and pro-inflammatory properties of uric acid can contribute to the initiation and promotion of tumor formation. Abnormalities in purine nucleotide metabolism can disrupt gene and protein expression and affect cell behavior, including malignant transformation, invasion, and metastasis, by influencing signal transduction pathways. The specific features of nucleotide metabolism may vary among different tumor types. Several studies have shown a correlation between elevated blood uric acid levels and the occurrence of colorectal, liver, kidney, and other cancers. BLCA research has highlighted the importance of increased anaerobic metabolic pathways in cancer stem-like cells for tumor development, progression, and resistance to treatment. Although previous studies have mainly focused on the impact of individual regulators of purine metabolism in BLCA, the collective contributions of multiple genes involved in purine metabolism remain unclear (26). Investigating distinct patterns of purine metabolism during BLCA progression may provide insights into its underlying mechanisms and guide the development of targeted therapeutic approaches.

In this study, we identified 112 DEGs associated with purine metabolism in BLCA. These DEGs were categorized into two distinct groups. Previous research has demonstrated a strong association between prognostic PMGs and BLCA outcomes. Notably, eight prognostic PMGs showed differential expression in high-risk individuals, with certain PMGs exhibiting overexpression in the high-risk population (*p* < 0.05). To further investigate the role of PMGs in BLCA, we conducted survival analysis to evaluate their prognostic value. Patients with low-risk PMGs exhibited a higher probability of survival. Additionally, we observed significant expression of CLDN6, CES1, SOST, SPRR2A, MYBPH, CGB5, and KRT1 in the high-risk group, suggesting their potential involvement as cancer-promoting genes in BLCA development. While these findings shed light on future research directions, further investigation is warranted to establish significant evidence regarding their influence on the expression of specific transcription factors involved in iron toxicity control, such as Fin56, NRF2, and SFRS9 (27–29). Furthermore, we observed significant expression of CRTAC1 in the low-risk group, indicating its potential role as a tumor suppressor gene in BLCA. These findings provide valuable insights for future studies aimed at unraveling the molecular mechanisms underlying BLCA progression and identifying potential therapeutic targets.

We identified these genes as being associated with BLCA and Purine Metabolism by reviewing the literature. MYBPH is a transcriptional target of TTF-1, a master regulator of lung development that plays a role as a lineage-survival oncogene in lung adenocarcinoma development (30). In an *in vitro* co-culture model of PC3 prostate cancer cells and osteoblasts, Aimy Sebastian revealed that reduced SOST expression in the tumor microenvironment might promote bone metastasis in prostate cancer *via* up-regulation of MALAT1 (31). Estrogen receptor β inhibits breast cancer cells migration and invasion through CLDN6-mediated autophagy (32). Melatonin inhibits lipid accumulation to repress prostate cancer progression by mediating the epigenetic modification of CES1 (33). Serum Small Proline-Rich Protein 2A (SPRR2A) Is a Noninvasive Biomarker in Gastric Cancer (34). Since these 8 PMGs were linked to the development of BLCA, these investigations support the validity and plausibility of our findings. The GSE13507, GSE48075, and GSE48276 Kaplan–Meier curves’ OS and ROC analyses suggested that a purine metabolism-related signature might be a valid prognostic predictor. Nonetheless, there has been a paucity of studies on the gene alterations associated with purine metabolism. As a result, more research is needed to uncover the mechanism of PMGs changes and to identify and confirm the existing findings.

In our study, we investigated the impact of purine metabolism on BLCA. Through KEGG analysis, we found that Purine Metabolism Deficiency, which is exacerbated by hypertension, can be effectively suppressed by Naringin, a bi-flavonoid, in a rat model (35). This suppression is mediated through the NOS/cAMP/PKA and DARPP-32, BDNF/TrkB pathways. Additionally, we discovered that MoImd4, a regulator in Magnaporthe oryzae, influences proliferation and pathogenicity by facilitating crosstalk between MoPdeH-cAMP signaling and purine metabolism. Our findings highlight the significant role of purine metabolism in BLCA (36). Notably, the nod-like receptor signaling pathway emerged as the most highly enriched pathway in GSEA. Recent studies have revealed that certain botanicals and natural products have the ability to modulate NOD-like receptor signaling. NOD-like receptors (NLRs) are critical regulators involved in carcinogenesis, angiogenesis, cancer cell stemness, and chemoresistance, particularly in response to inflammation (37, 38). NLRs detect pathogen-associated molecular patterns, leading to the activation of other signaling regulators such as Rip2 kinase, NF-B, MAPK, and ASC/caspase-1, ultimately resulting in cytokine production (39). Considering these findings, it is plausible that PMGs may exert their influence on BLCA cell migration and proliferation through modulation of the nod-like receptor signaling pathway. Furthermore, several clinical studies have confirmed the impact of purine metabolism on the survival of BLCA patients. For instance, in Jacyna’s study, a distinct profile of 17 metabolites was observed in the urine of BCa patients compared to healthy individuals. These metabolites are primarily involved in amino acid metabolism, pyrimidine and purine metabolism, and energy metabolism, indicating their potential significance in the pathophysiology of BCa (40).

This study provides an accurate prediction of the survival outcomes in patients with BLCA. The prognostic model based on PMGs demonstrates that an increase in the risk score is associated with a higher risk of death. The PMGs identified in this study hold promise as valuable biomarkers for predicting patient outcomes in BLCA. Recent research has unveiled the intricate connection between various cell death mechanisms and the immune response against cancer (41). ICIs have revolutionized cancer treatment by unleashing the anticancer potential of the immune system. Activation of programmed cell death pathways such as pyroptosis, ferroptosis, and necroptosis in ICI-resistant tumors has been found to synergistically enhance the anticancer efficacy. Furthermore, the involvement of insulin in the regulation of immune checkpoints has been investigated, particularly in the context of pancreatic ductal adenocarcinoma cells (42). Insulin signaling pathways, including increased expression of InsR-A in A818-6 cells and modulation of the adaptor protein Gab1 in BxPc3 cells, contribute to the enhancement of PD-L1 expression (43). Moreover, Kyrollis Attalla has identified TIM-3 and TIGIT as promising targets for monotherapy or combination therapy with other immune checkpoint inhibitors in patients with urothelial cancer of the bladder. These findings provide valuable insights into the interplay between ICIs, mRNA chemical modifications (such as m6a), and purine metabolism. The observed alterations in PMGs are likely to play a crucial role in the initiation and progression of BLCA, shedding light on its etiology and development.

The link between purine metabolism and BLCA has been relatively underexplored in scientific literature. However, recent bioinformatics studies have provided valuable insights into this association in the context of cancer (7, 44–46). For instance, Hu et al. identified the susceptibility of pancreatic cancer to the combined targeting of *de novo* purine synthesis and glycolysis in the presence of MTAP deficiency. Similarly, Yang et al. developed a Purine and Uric Metabolism Signature, highlighting the prognostic value of peripheral blood uric acid levels in hepatocellular carcinoma (HCC). Additionally, Su et al. constructed a novel HCC prediction model incorporating five PMGs, enabling the prediction of patient prognosis. Building upon these seminal works, our study supplements existing knowledge by incorporating additional PMGs data from the continually updated TCGA database. TCGA data served as the primary analysis, with GEO data utilized for model validation, ensuring robustness. The inclusion of GO and KEGG analyses, as well as GSEA analysis, further strengthens the credibility of our findings. Moreover, to enhance the reliability of the results, multiple databases were employed to evaluate immune cells and functions. Nevertheless, it is essential to acknowledge certain limitations, including the reliance on public databases and the need for further validation of protein expression in larger datasets, as protein expression may diverge from RNA expression.

## 5. Conclusion

The present study delineates the regulatory functions of PMGs and elucidates the underlying factors governing diverse clinical outcomes and immunotherapy responses in BLCA across various PMG regulatory patterns. A meticulous exploration of individual PMG regulatory patterns not only facilitates the development of personalized immunotherapy strategies for BLCA patients but also enhances our understanding of BLCA immune cell characterization. Moreover, the primary objective of this research is to identify and comprehensively profile the gene signatures associated with PMG-related regulators in BLCA. The diverse array of PMG modification patterns significantly contributes to the intricate diversity and complexity of the TME. Additionally, a predictive model based on PMG signatures has been devised, offering the potential to forecast the clinical course of BLCA.

## Data availability statement

The original contributions presented in the study are included in the article/Supplementary material, further inquiries can be directed to the corresponding authors.

## Author contributions

ZW and ZF made substantial contributions to the conception and design of the works. ZF and HW were responsible for data acquisition, analysis, or interpretation. KC and CL drafted or revised work for important intellectual content, and final approval of the version to be released. All authors are agreementing to be accountable for all aspects of the work in ensuring that questions related to the accuracy or integrity of any part of the work are appropriately investigated and resolved.

## References

[1] Lenis AT Lec PM Chamie K Mshs MD. Bladder cancer: a review. JAMA. (2020) 324:1980–91. doi: 10.1001/jama.2020.17598 33201207

[2] Antoni S Ferlay J Soerjomataram I Znaor A Jemal A Bray F. Bladder cancer incidence and mortality: a global overview and recent trends. Eur Urol. (2017) 71:96–108. doi: 10.1016/j.eururo.2016.06.010 27370177

[3] Buisan O Orsola A Oliveira M Martinez R Etxaniz O Areal J. Role of inflammation in the perioperative management of urothelial bladder cancer with squamous-cell features: impact of neutrophil-to-lymphocyte ratio on outcomes and response to neoadjuvant chemotherapy. Clin Genitourin Cancer. (2017) 15:e697–706. doi: 10.1016/j.clgc.2017.01.024 28274590

[4] DeGeorge KC Holt HR Hodges SC. Bladder cancer: diagnosis and treatment. Am Fam Physician. (2017) 96:507–14. PMID: 29094888

[5] Barani M Hosseinikhah SM Rahdar A Farhoudi L Arshad R Cucchiarini M. Nanotechnology in bladder cancer: diagnosis and treatment. Cancers (Basel). (2021) 13:2214. doi: 10.3390/cancers13092214 34063088 PMC8125468

[6] Patel VG Oh WK Galsky MD. Treatment of muscle-invasive and advanced bladder cancer in 2020. CA Cancer J Clin. (2020) 70:404–23. doi: 10.3322/caac.21631 32767764

[7] Yang S Zhang B Tan W Qi L Ma X Wang X. A novel purine and uric metabolism signature predicting the prognosis of hepatocellular carcinoma. Front Genet. (2022) 13:942267. doi: 10.3389/fgene.2022.942267 35903353 PMC9315342

[8] Liu J Hong S Yang J Zhang X Wang Y Wang H. Targeting purine metabolism in ovarian cancer. J Ovarian Res. (2022) 15:93. doi: 10.1186/s13048-022-01022-z 35964092 PMC9375293

[9] Yin J Ren W Huang X Deng J Li T Yin Y. Potential mechanisms connecting purine metabolism and cancer therapy. Front Immunol. (2018) 9:1697. doi: 10.3389/fimmu.2018.01697 30105018 PMC6077182

[10] Shatova OP Butenko EV Khomutov EV Kaplun DS Sedakov IE Zinkovych II. Metformin impact on purine metabolism in breast cancer. Biomed Khim. (2016) 62:302–5. doi: 10.18097/PBMC20166203302 27420623

[11] Chen X Chen J. MiR-10b-5p-mediated upregulation of PIEZO1 predicts poor prognosis and links to purine metabolism in breast cancer. Genomics. (2022) 114:110351. doi: 10.1016/j.ygeno.2022.110351 35351580

[12] Koundouros N Poulogiannis G. Reprogramming of fatty acid metabolism in cancer. Br J Cancer. (2020) 122:4–22. doi: 10.1038/s41416-019-0650-z 31819192 PMC6964678

[13] Wu L Zhang X Zheng L Zhao H Yan G Zhang Q. RIPK3 orchestrates fatty acid metabolism in tumor-associated macrophages and hepatocarcinogenesis. Cancer Immunol Res. (2020) 8:710–21. doi: 10.1158/2326-6066.CIR-19-0261 32122992

[14] Hu J Yu A Othmane B Qiu D Li H Li C. Siglec15 shapes a non-inflamed tumor microenvironment and predicts the molecular subtype in bladder cancer. Theranostics. (2021) 11:3089–108. doi: 10.7150/thno.53649 33537076 PMC7847675

[15] Hu J Othmane B Yu A Li H Cai Z Chen X. 5MC regulator-mediated molecular subtypes depict the hallmarks of the tumor microenvironment and guide precision medicine in bladder cancer. BMC Med. (2021) 19:289. doi: 10.1186/s12916-021-02163-6 34836536 PMC8627095

[16] Cai Z Chen J Yu Z Li H Liu Z Deng D. BCAT2 shapes a noninflamed tumor microenvironment and induces resistance to anti-PD-1/PD-L1 immunotherapy by negatively regulating proinflammatory chemokines and anticancer immunity. Adv Sci (Weinh). (2023) 10:e2207155. doi: 10.1002/advs.202207155 36642843 PMC10015882

[17] Liu Z Tang Q Qi T Othmane B Yang Z Chen J. A robust hypoxia risk score predicts the clinical outcomes and tumor microenvironment immune characters in bladder cancer. Front Immunol. (2021) 12:725223. doi: 10.3389/fimmu.2021.725223 34484235 PMC8415032

[18] Zhao E Chen S Dang Y. Development and external validation of a novel immune checkpoint-related gene signature for prediction of overall survival in hepatocellular carcinoma. Front Mol Biosci. (2020) 7:620765. doi: 10.3389/fmolb.2020.620765 33553243 PMC7859359

[19] Wu Z Huang X Cai M Huang P Guan Z. Novel necroptosis-related gene signature for predicting the prognosis of pancreatic adenocarcinoma. Aging (Albany NY). (2022) 14:869–91. doi: 10.18632/aging.203846 35077391 PMC8833111

[20] Xu D Ji Z Qiang L. Molecular characteristics, clinical implication, and cancer immunity interactions of Pyroptosis-related genes in breast cancer. Front Med (Lausanne). (2021) 8:702638. doi: 10.3389/fmed.2021.702638 34589498 PMC8473741

[21] Buraschi S Neill T Xu SQ Palladino C Belfiore A Iozzo RV. Progranulin/EphA2 axis: a novel oncogenic mechanism in bladder cancer. Matrix Biol. (2020) 93:10–24. doi: 10.1016/j.matbio.2020.03.009 32417448 PMC8162889

[22] Facchini G Cavaliere C Romis L Mordente S Facchini S Iovane G. Advanced/metastatic bladder cancer: current status and future directions. [journal article; research support, non-U.S. Gov’t; review]. Eur Rev Med Pharmacol Sci. (2020) 24:11536–52. doi: 10.26355/eurrev_202011_23795 33275220

[23] Tran L Xiao JF Agarwal N Duex JE Theodorescu D. Advances in bladder cancer biology and therapy. [journal article; research support, N.I.H., extramural; review]. Nat Rev Cancer. (2021) 21:104–21. doi: 10.1038/s41568-020-00313-1 33268841 PMC10112195

[24] Sylvester RJ van der Meijden AP Oosterlinck W Witjes JA Bouffioux C Denis L. Predicting recurrence and progression in individual patients with stage ta T1 bladder cancer using EORTC risk tables: a combined analysis of 2596 patients from seven EORTC trials. Eur Urol. (2006) 49:465–466, 475–477. doi: 10.1016/j.eururo.2005.12.031 16442208

[25] Yu F Quan F Xu J Zhang Y Xie Y Zhang J. Breast cancer prognosis signature: linking risk stratification to disease subtypes. Brief Bioinform. (2019) 20:2130–40. doi: 10.1093/bib/bby073 30184043

[26] Jiang Z Shen H Tang B Yu Q Ji X Wang L. Quantitative proteomic analysis reveals that proteins required for fatty acid metabolism may serve as diagnostic markers for gastric cancer. Clin Chim Acta. (2017) 464:148–54. doi: 10.1016/j.cca.2016.11.032 27884752

[27] Sun Y Berleth N Wu W Schlutermann D Deitersen J Stuhldreier F. Fin56-induced ferroptosis is supported by autophagy-mediated GPX4 degradation and functions synergistically with mTOR inhibition to kill bladder cancer cells. Cell Death Dis. (2021) 12:1028. doi: 10.1038/s41419-021-04306-2 34716292 PMC8556316

[28] Xiang Y Chen X Wang W Zhai L Sun X Feng J. Natural product erianin inhibits bladder cancer cell growth by inducing ferroptosis via NRF2 inactivation. Front Pharmacol. (2021) 12:775506. doi: 10.3389/fphar.2021.775506 34776986 PMC8585785

[29] Wang R Xing R Su Q Yin H Wu D Lv C. Knockdown of SFRS9 inhibits progression of colorectal cancer through triggering ferroptosis mediated by GPX4 reduction. Front Oncol. (2021) 11:683589. doi: 10.3389/fonc.2021.683589 34336668 PMC8322952

[30] Attalla K Farkas AM Anastos H Audenet F Galsky MD Bhardwaj N. TIM-3 and TIGIT are possible immune checkpoint targets in patients with bladder cancer. Urol Oncol. (2022) 40:403–6. doi: 10.1016/j.urolonc.2020.06.007 32665122 PMC7980780

[31] Sebastian A Hum NR Hudson BD Loots GG. Cancer-osteoblast interaction reduces sost expression in osteoblasts and up-regulates lncRNA MALAT1 in prostate cancer. Microarrays (Basel). (2015) 4:503–19. doi: 10.3390/microarrays4040503 27600237 PMC4996404

[32] Song P Li Y Dong Y Liang Y Qu H Qi D. Estrogen receptor beta inhibits breast cancer cells migration and invasion through CLDN6-mediated autophagy. J Exp Clin Cancer Res. (2019) 38:354. doi: 10.1186/s13046-019-1359-9 31412908 PMC6694553

[33] Zhou L Zhang C Yang X Liu L Hu J Hou Y. Melatonin inhibits lipid accumulation to repress prostate cancer progression by mediating the epigenetic modification of CES1. Clin Transl Med. (2021) 11:e449. doi: 10.1002/ctm2.449 34185414 PMC8181204

[34] Xu X Wei S Chen Y Yu D Wang X Dong X. Serum small proline-rich protein 2A (SPRR2A) is a noninvasive biomarker in gastric cancer. Dis Markers. (2020) 2020:8493796. doi: 10.1155/2020/8493796 32908616 PMC7475742

[35] Akintunde JK Abinu OS Taiwo KF Sodiq RA Folayan AD Ate AD. Disorders of hippocampus facilitated by hypertension in purine metabolism deficiency is repressed by naringin, a bi-flavonoid in a rat model via NOS/cAMP/PKA and DARPP-32, BDNF/TrkB pathways. Neurotox Res. (2022) 40:2148–66. doi: 10.1007/s12640-022-00578-4 36098940

[36] Yang L Ru Y Cai X Yin Z Liu X Xiao Y. MoImd4 mediates crosstalk between MoPdeH-cAMP signalling and purine metabolism to govern growth and pathogenicity in Magnaporthe oryzae. Mol Plant Pathol. (2019) 20:500–18. doi: 10.1111/mpp.12770 30426699 PMC6422694

[37] Fan Z Pan J Wang H Zhang Y. NOD-like receptor X1, tumor necrosis factor receptor-associated factor 6 and NF-kappaB are associated with clinicopathological characteristics in gastric cancer. Exp Ther Med. (2021) 21:208. doi: 10.3892/etm.2021.9640 33574909 PMC7818523

[38] Peng L Hu Y Chen D Linghu R Wang Y Kou X. Ubiquitin specific protease 21 upregulation in breast cancer promotes cell tumorigenic capability and is associated with the NOD-like receptor signaling pathway. Oncol Lett. (2016) 12:4531–7. doi: 10.3892/ol.2016.5263 28105162 PMC5228564

[39] Liu P Lu Z Liu L Li R Liang Z Shen M. NOD-like receptor signaling in inflammation-associated cancers: from functions to targeted therapies. Phytomedicine. (2019) 64:152925. doi: 10.1016/j.phymed.2019.152925 31465982

[40] Jacyna J Wawrzyniak R Balayssac S Gilard V Malet-Martino M Sawicka A. Urinary metabolomic signature of muscle-invasive bladder cancer: a multiplatform approach. Talanta. (2019) 202:572–9. doi: 10.1016/j.talanta.2019.05.039 31171223

[41] Kuo CJ Hansen M Troemel E. Autophagy and innate immunity: insights from invertebrate model organisms. Autophagy. (2018) 14:233–42. doi: 10.1080/15548627.2017.1389824 29130360 PMC5902216

[42] Tang R Xu J Zhang B Liu J Liang C Hua J. Ferroptosis, necroptosis, and pyroptosis in anticancer immunity. J Hematol Oncol. (2020) 13:110. doi: 10.1186/s13045-020-00946-7 32778143 PMC7418434

[43] Heckl SM Mau F Senftleben A Daunke T Beckinger S Abdullazade S. Programmed death-ligand 1 (PD-L1) expression is induced by insulin in pancreatic ductal adenocarcinoma cells pointing to its role in immune checkpoint control. Med Sci (Basel). (2021) 9:48. doi: 10.3390/medsci9030048 34202040 PMC8293454

[44] Hu Q Qin Y Ji S Shi X Dai W Fan G. MTAP deficiency-induced metabolic reprogramming creates a vulnerability to cotargeting de novo purine synthesis and glycolysis in pancreatic cancer. Cancer Res. (2021) 81:4964–80. doi: 10.1158/0008-5472.CAN-20-0414 34385182

[45] Su WJ Lu PZ Wu Y Kalpana K Yang CK Lu GD. Identification of key genes in purine metabolism as prognostic biomarker for hepatocellular carcinoma. Front Oncol. (2020) 10:583053. doi: 10.3389/fonc.2020.583053 33520699 PMC7841304

[46] Ludwig N Gillespie DG Reichert TE Jackson EK Whiteside TL. Purine metabolites in tumor-derived exosomes may facilitate immune escape of head and neck squamous cell carcinoma. Cancers (Basel). (2020) 12:1602. doi: 10.3390/cancers12061602 32560461 PMC7352909

