# Supplementary material for: Development and validation of prognostic index based on purine metabolism genes in patients with bladder cancer
Source: Front Med (Lausanne). 2023 Sep 14;10:1193133. doi: 10.3389/fmed.2023.1193133 (PMC10536175; doi:10.3389/fmed.2023.1193133)
Supplement: Supplementary file 1 [file Data_Sheet_1.doc]

Development and Validation of Prognostic Index Based On Purine Metabolism Genes in Patients with Bladder Cancer

**Supplementary appendix to the manuscript**

Contents of supplementary appendix

[Appendix 1 3](#__RefHeading___Toc22751)

[Datasets and Purine Metabolism 3](#__RefHeading___Toc16995)

[Table 1a. The clinical characteristics of patients. 3](#__RefHeading___Toc32115)

[Table 1b. Purine Metabolism genes 4](#__RefHeading___Toc23899)

[Appendix 2 5](#__RefHeading___Toc25225)

[Table 2a. m6A genes. 5](#__RefHeading___Toc7099)

[Table 2b. m1A genes. 5](#__RefHeading___Toc12198)

[Table 2c. m7G genes. 5](#__RefHeading___Toc10324)

[Table 2d. m5C genes. 6](#__RefHeading___Toc2090)

[Appendix 3 6](#__RefHeading___Toc5496)

[DEGs linked to FAMGs 6](#__RefHeading___Toc1998)

[Table 3. 112 DEGs linked to purine metabolism genes. 6](#__RefHeading___Toc16384)

[Appendix 4 11](#__RefHeading___Toc4093)

[The drug prediction of the model 11](#__RefHeading___Toc952)

[Appendix 5 12](#__RefHeading___Toc21793)

[Correlation analysis of gene expression in prognostic signatures and drug sensitivity 13](#__RefHeading___Toc5131)

[Appendix 6 13](#__RefHeading___Toc21266)

[hub genes analysis 13](#__RefHeading___Toc8988)

[Table 4. Hub genes. 13](#__RefHeading___Toc19020)

[Appendix 7 18](#__RefHeading___Toc4796)

[The gene expression profile and clinical characteristics 18](#__RefHeading___Toc13216)

[Table 5. The gene expression profile and clinical characteristics. 18](#__RefHeading___Toc1485)

[Appendix 8 22](#__RefHeading___Toc18025)

[6 risk PRGs 22](#__RefHeading___Toc7709)

[Table 6. 6 risk PRGs. 22](#__RefHeading___Toc19868)

[Appendix 9 51](#__RefHeading___Toc20925)

[GO and KEGG enrichment analysis 51](#__RefHeading___Toc10795)

[Table 7a. GO enrichment analysis. 51](#__RefHeading___Toc23644)

[Table 7b. KEGG enrichment analysis. 81](#__RefHeading___Toc5514)

[Appendix 10 84](#__RefHeading___Toc13360)

[gene set enrichment analyses (GSEA) 84](#__RefHeading___Toc3420)

[Table 8a. GSEA of high rish. 84](#__RefHeading___Toc26481)

[Table 8b. GSEA of low rish. 97](#__RefHeading___Toc22932)

# Appendix 1

**Datasets and Purine Metabolism**

**Table 1a. The clinical characteristics of patients.**

| TCGA | | GEO  (GSE13507, GSE48075, and GSE48276) | |
| --- | --- | --- | --- |
| Variables | Number of samples | Variables | Number of samples |
| Gender |  | Gender |  |
| Male/Female | 304/108 | Male/Female | 96/211 |
| Age at diagnosis |  | Age at diagnosis |  |
| ≤65/>65 | 162/250 | ≤65/>65 | 127/182 |
| Grade |  | Grade |  |
| High/Low/NA | 388/21/3 | High/Low | 60/105 |
| Stage |  | Stage |  |
| I/II/III/IV/NA | 2/131/141/136/2 | I/II/III/IV/NA | Unknow |
| T |  | T |  |
| T1/T2/T3/T4/NA | 3/120/196/59/34 | T1/T2/T3/T4/NA | 90/134/85/27/26 |
| M |  | M |  |
| M0/M1/NA | 196/11/205 | M0/M1/NA | 337/9/6 |
| N |  | N |  |
| N0/N1/N2/N3/NA | 239/47/76/8/42 | N0/N1/N2/N3/NA | 323/11/7/1/10 |

**Table 1b. Purine Metabolism genes**

| NUDT9 | POLA1 | ZNRD1 | AK2 | ENTPD4 | PDE6B |
| --- | --- | --- | --- | --- | --- |
| ADPRM | POLA2 | TWISTNB | AK1 | ENTPD5 | PDE6C |
| NUDT5 | PRIM1 | POLR1E | PDE6A | ENTPD6 | PDE6D |
| PGM1 | PRIM2 | POLR2A | AK8 | NUDT16 | PDE6G |
| PGM2 | POLD1 | POLR2B | AK6 | ITPA | PDE6H |
| PRPS1L1 | POLD2 | POLR2C | AK3 | XDH | PDE9A |
| PRPS2 | POLD3 | POLR2D | ENTPD2 | NUDT2 | PDE10A |
| PRPS1 | POLD4 | POLR2E | NTPCR | GMPS | PDE11A |
| PPAT | POLE | POLR2F | PNPT1 | GMPR | ADSSL1 |
| GART | POLE2 | POLR2G | PDE4A | GMPR2 | ADSS |
| PFAS | POLE3 | POLR2H | PDE4B | GDA | AMPD2 |
| PAICS | POLE4 | POLR2I | PDE4C | GUK1 | AMPD3 |
| ADSL | HDDC3 | POLR2L | PDE4D | PKM | AMPD1 |
| ATIC | PRUNE1 | POLR2J | PDE7A | PKLR | ADK |
| APRT | ADCY1 | POLR2J3 | PDE7B | RRM1 | DCK |
| NT5C2 | ADCY2 | POLR2J2 | PDE8B | RRM2B | ADA |
| NT5C1A | ADCY3 | POLR2K | PDE8A | RRM2 | CECR1 |
| NT5C1B | ADCY4 | POLR3A | FHIT | DGUOK | AK7 |
| NT5C | ADCY5 | POLR3B | ENPP4 | POLR1A | AK4 |
| NT5M | ADCY6 | POLR3C | PAPSS2 | POLR1B | AK5 |
| NT5C3A | ADCY7 | POLR3D | PAPSS1 | NME2 | NPR1 |
| NT5C3B | ADCY8 | POLR3E | ENPP1 | NME4 | NPR2 |
| NT5C1B-RDH14 | ADCY9 | RPC5 | ENPP3 | NME1 | PDE1A |
| NT5E | ADCY10 | POLR1C | URAD | NME3 | PDE1B |
| PNP | GUCY1A2 | POLR3K | ALLC | NME1-NME2 | PDE1C |
| HPRT1 | GUCY1A3 | POLR1D | NME7 | AK9 | PDE2A |
| IMPDH1 | GUCY1B3 | POLR3H | GUCY2F | ENTPD3 | PDE3A |
| IMPDH2 | GUCY2C | POLR3GL | POLR3F | ENTPD8 | PDE3B |
| NME6 | GUCY2D | POLR3G | CANT1 | ENTPD1 | PDE5A |

# Appendix 2

**mRNA chemical modifications**

**Table 2a. m6A genes.**

| METTL3 | YTHDC1 | ZC3H13 | RBM15 | HNRNPC |
| --- | --- | --- | --- | --- |
| METTL14 | YTHDC2 | FTO | YTHDF2 | KIAA1429 |
| WTAP | YTHDF1 | ALKBH5 |  |  |

**Table 2b. m1A genes.**

| YTHDF2 | YTHDF1 | TRMT61A | YTHDC1 | YTHDF3 |
| --- | --- | --- | --- | --- |
| RRP8 | ALKBH1 | ALKBH3 | TRMT6 |  |

**Table 2c. m7G genes.**

| METTL1 | EIF4E | EIF4A1 | NUDT4 | NCBP1 |
| --- | --- | --- | --- | --- |
| WDR4 | EIF4E1B | EIF4G3 | NUDT48 | NCBP2 |
| NSUN2 | EIF4E2 | IFIT5 | AGO2 | NCBP3 |
| DCP2 | EIF4E3 | LSM1 | CYFIP1 | EIF3D |
| DCPS | GEMIN5 | NCBP2L | NUDT16 | NUDT11 |
| NUDT10 | LARP1 | SNUPN | NUDT3 |  |

**Table 2d. m5C genes.**

| NSUN1 | DNMT2 | NSUN7 | TET2 | NSUN4 |
| --- | --- | --- | --- | --- |
| NSUN | DNMT3A | ALYREF | TRDMT1 | NSUN5 |
| NSUN3 | DNMT3B | DNMT1 | YBX1 | NSUN6 |

# Appendix 3

## **DEGs linked to FAMGs**

**Table 3. 112 DEGs linked to purine metabolism genes.**

| gene | conMean | treatMean | logFC | pValue |
| --- | --- | --- | --- | --- |
| ADPRM | 4.399326316 | 3.335596845 | -0.399337687 | 0.00026441 |
| NUDT5 | 6.047994737 | 13.17579782 | 1.123361533 | 2.64E-08 |
| PRPS2 | 21.32115263 | 28.27451019 | 0.407216599 | 0.021074213 |
| PPAT | 1.613394737 | 3.461698301 | 1.10138054 | 1.33E-08 |
| GART | 6.992694737 | 12.17168592 | 0.799608581 | 1.39E-07 |
| PFAS | 2.801042105 | 4.581432282 | 0.709835025 | 8.55E-05 |
| PAICS | 9.914368421 | 22.36865194 | 1.173885538 | 1.49E-09 |
| ADSL | 0.287336842 | 0.49736068 | 0.791549469 | 2.61E-06 |
| ATIC | 17.52768947 | 26.71810583 | 0.608181901 | 4.55E-05 |
| APRT | 47.08631579 | 79.30959296 | 0.752187534 | 3.90E-05 |
| NT5C1A | 0.031315789 | 0.011026942 | -1.505857526 | 0.019358539 |
| NT5C | 9.244805263 | 16.26957961 | 0.815462138 | 1.31E-05 |
| NT5M | 1.736805263 | 1.349306796 | -0.364217587 | 0.013490964 |
| NT5C3A | 6.517136842 | 10.50475558 | 0.688732402 | 2.11E-05 |
| NT5C3B | 11.58985263 | 15.26842743 | 0.397689257 | 0.013139808 |
| NT5E | 8.641731579 | 8.926609709 | 0.046791929 | 0.003054518 |
| HPRT1 | 15.75738421 | 31.3989216 | 0.994686949 | 4.14E-07 |
| IMPDH1 | 12.73516842 | 24.14305607 | 0.922790267 | 4.07E-05 |
| NME6 | 2.188647368 | 3.355904854 | 0.616662285 | 2.04E-06 |
| NME2 | 0.231384211 | 0.631837379 | 1.449262868 | 1.44E-07 |
| NME1 | 3.801884211 | 10.66328592 | 1.487865578 | 2.73E-10 |
| NME3 | 23.9072 | 31.11527937 | 0.380178027 | 0.036447038 |
| ENTPD8 | 0.061089474 | 1.204743447 | 4.301658332 | 0.012053775 |
| ENTPD1 | 4.975915789 | 2.22391165 | -1.161862595 | 4.10E-05 |
| CANT1 | 11.55090526 | 20.868725 | 0.853336538 | 1.24E-06 |
| ENTPD6 | 5.930252632 | 12.73940704 | 1.103132658 | 2.51E-09 |
| NUDT16 | 7.299194737 | 5.889121359 | -0.309684908 | 0.007752078 |
| ITPA | 20.91566842 | 40.96298034 | 0.969736581 | 1.34E-07 |
| XDH | 1.430694737 | 2.871271117 | 1.004973681 | 0.040873346 |
| GMPS | 7.038289474 | 11.73734806 | 0.737809726 | 2.43E-06 |
| GUK1 | 35.21089474 | 45.96097063 | 0.384387378 | 0.001672044 |
| PKM | 136.0835526 | 223.6837534 | 0.716967764 | 0.001175497 |
| PKLR | 0.006978947 | 0.067425 | 3.27220226 | 0.003223702 |
| RRM1 | 14.12525789 | 20.18890874 | 0.515285723 | 0.033518889 |
| RRM2 | 3.198436842 | 11.86983228 | 1.89186065 | 7.97E-08 |
| DGUOK | 17.84007895 | 29.93812597 | 0.746861917 | 8.11E-07 |
| POLR1A | 3.6594 | 5.143890534 | 0.49125282 | 0.001092782 |
| POLR1B | 2.255005263 | 3.919981796 | 0.797716154 | 1.60E-06 |
| POLR1E | 13.11182632 | 10.03986481 | -0.385128807 | 0.007752084 |
| POLR2D | 4.689589474 | 6.90170267 | 0.557490688 | 4.71E-06 |
| POLR2E | 27.47598421 | 33.91892184 | 0.303919152 | 0.003620625 |
| POLR2F | 4.463021053 | 6.94216335 | 0.6373647 | 1.55E-06 |
| POLR2G | 21.16087895 | 41.6199301 | 0.975874989 | 1.81E-09 |
| POLR2H | 10.79895263 | 25.00149587 | 1.211123021 | 2.45E-10 |
| POLR2I | 2.978510526 | 5.611044417 | 0.913678276 | 2.99E-07 |
| POLR2L | 69.31041053 | 93.2353466 | 0.427804937 | 0.000447486 |
| POLR2J | 22.2345 | 41.34390825 | 0.894874811 | 2.23E-07 |
| POLR2J3 | 0.01 | 0.044654612 | 2.158809176 | 1.60E-06 |
| POLR2J2 | 0.035315789 | 0.089865534 | 1.347454559 | 0.003136299 |
| POLR2K | 23.24354737 | 41.42327524 | 0.833611363 | 2.43E-06 |
| POLR3A | 2.894268421 | 3.753154369 | 0.374904903 | 0.000438068 |
| POLR3B | 2.401678947 | 3.255493204 | 0.43883282 | 0.003316532 |
| POLR3C | 5.645489474 | 7.217371117 | 0.354374769 | 0.004514837 |
| POLR3E | 3.281084211 | 4.174754612 | 0.347518778 | 0.016273481 |
| POLR1C | 3.697273684 | 6.217155583 | 0.749792842 | 2.65E-05 |
| POLR3K | 7.044 | 11.19791141 | 0.668762857 | 1.67E-06 |
| POLR1D | 8.503363158 | 11.56664005 | 0.443864384 | 0.000390889 |
| POLR3H | 2.631057895 | 3.237213592 | 0.299109564 | 0.01272909 |
| POLR3GL | 7.686521053 | 6.47935801 | -0.246479902 | 0.010381916 |
| POLR3G | 0.505173684 | 1.131371359 | 1.163221162 | 0.011107577 |
| POLR3F | 4.261678947 | 5.609759709 | 0.396517063 | 0.003017345 |
| POLA1 | 1.205610526 | 1.771375 | 0.555105744 | 0.013777848 |
| POLA2 | 0.733868421 | 1.874884466 | 1.353208373 | 2.16E-09 |
| PRIM1 | 0.782768421 | 2.119496359 | 1.437064028 | 1.58E-07 |
| PRIM2 | 1.774515789 | 3.548624029 | 0.99983432 | 8.06E-09 |
| POLD1 | 2.675657895 | 7.96053568 | 1.572971849 | 7.94E-11 |
| POLD2 | 19.87543158 | 35.01331141 | 0.816917324 | 4.13E-06 |
| POLD3 | 3.995942105 | 5.4867 | 0.457403014 | 0.004009303 |
| POLD4 | 2.088231579 | 3.151276699 | 0.593654725 | 0.000290762 |
| POLE | 1.870484211 | 4.227539806 | 1.176406551 | 2.84E-09 |
| POLE2 | 0.787910526 | 2.820518932 | 1.839856906 | 1.15E-09 |
| POLE3 | 18.53132632 | 28.84271602 | 0.638240884 | 5.94E-05 |
| POLE4 | 16.29894211 | 22.58602354 | 0.470651966 | 0.000497475 |
| HDDC3 | 0.770036842 | 1.201447087 | 0.641773735 | 0.000323048 |
| PRUNE1 | 9.935005263 | 13.35599272 | 0.426894575 | 0.04782955 |
| ADCY2 | 0.394294737 | 0.093984466 | -2.068780224 | 4.26E-09 |
| ADCY4 | 1.938257895 | 0.954192718 | -1.022407959 | 6.70E-06 |
| ADCY5 | 9.547236842 | 1.173913835 | -3.02375673 | 3.19E-11 |
| ADCY9 | 4.299368421 | 1.856462621 | -1.211568475 | 8.06E-06 |
| ADCY10 | 0.029073684 | 0.127999029 | 2.138347061 | 0.040963347 |
| GUCY2D | 0.0531 | 0.131466262 | 1.307908846 | 0.001916192 |
| NPR1 | 5.704478947 | 1.752181311 | -1.702943047 | 9.97E-09 |
| NPR2 | 3.807189474 | 1.317743204 | -1.530657119 | 1.93E-07 |
| PDE1A | 3.483605263 | 0.558007767 | -2.642224049 | 6.20E-10 |
| PDE1B | 0.953805263 | 0.894867718 | -0.092020308 | 0.000249323 |
| PDE1C | 1.881152632 | 0.175818447 | -3.419458561 | 7.71E-09 |
| PDE2A | 5.715273684 | 1.058530825 | -2.432759303 | 8.41E-12 |
| PDE3A | 0.901194737 | 0.428712621 | -1.071827997 | 4.32E-06 |
| PDE3B | 2.591910526 | 1.271446117 | -1.027545594 | 9.83E-05 |
| PDE5A | 7.480094737 | 1.609543932 | -2.216404587 | 4.61E-08 |
| PDE6C | 0.082168421 | 0.127932282 | 0.638724401 | 0.026934305 |
| AMPD2 | 3.579205263 | 5.475903155 | 0.613457649 | 2.61E-05 |
| ADK | 5.818805263 | 9.601445631 | 0.722528675 | 0.000936942 |
| DCK | 4.867947368 | 7.874188592 | 0.693817697 | 0.000205685 |
| ADA | 3.049163158 | 6.459507767 | 1.083010882 | 0.009939916 |
| AK7 | 0.163968421 | 0.210010922 | 0.357046372 | 0.045746766 |
| AK4 | 7.141942105 | 3.742423786 | -0.932343503 | 0.000949626 |
| AK5 | 0.215789474 | 0.339274029 | 0.652826507 | 0.043349708 |
| AK2 | 9.486863158 | 14.60160291 | 0.622123709 | 6.96E-08 |
| AK3 | 23.04864737 | 19.32984733 | -0.253851844 | 0.001231106 |
| PNPT1 | 5.031947368 | 8.485930583 | 0.753956045 | 4.10E-07 |
| PDE4B | 4.385778947 | 1.223103883 | -1.842286156 | 8.82E-09 |
| PDE4C | 0.009784211 | 0.00564199 | -0.794251263 | 0.006517301 |
| PDE4D | 5.578605263 | 1.395348058 | -1.999279436 | 7.59E-10 |
| PDE7A | 2.454247368 | 4.420761408 | 0.849014205 | 0.00046689 |
| PDE7B | 3.196247368 | 1.045466019 | -1.612232897 | 2.85E-08 |
| ENPP4 | 4.7491 | 3.467987864 | -0.453555285 | 0.018774494 |
| PAPSS2 | 4.950442105 | 4.329154612 | -0.193472047 | 0.048042077 |
| ENPP1 | 1.074336842 | 0.875247087 | -0.295684139 | 1.08E-05 |
| ENPP3 | 0.222310526 | 0.066632524 | -1.738277807 | 3.51E-08 |
| URAD | 0.020794737 | 0.014062136 | -0.564402683 | 0.023226066 |
| ALLC | 0.030063158 | 0.015687136 | -0.938414584 | 3.59E-05 |

# Appendix 4

**The drug prediction of the model**

**
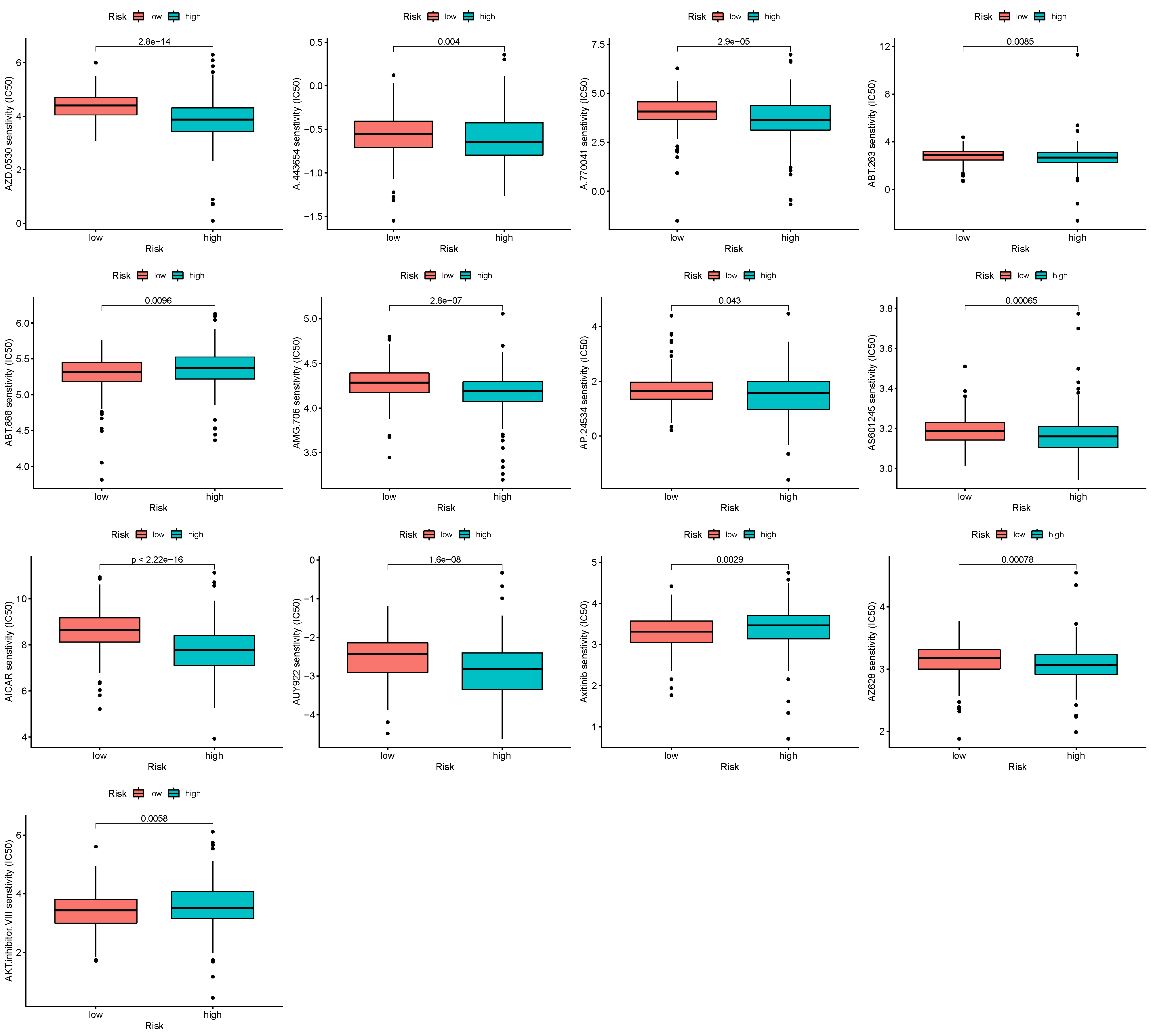
**

Figure S1. The drug prediction of the model.

# Appendix 5

## **Correlation analysis of gene expression in prognostic signatures and drug sensitivity**


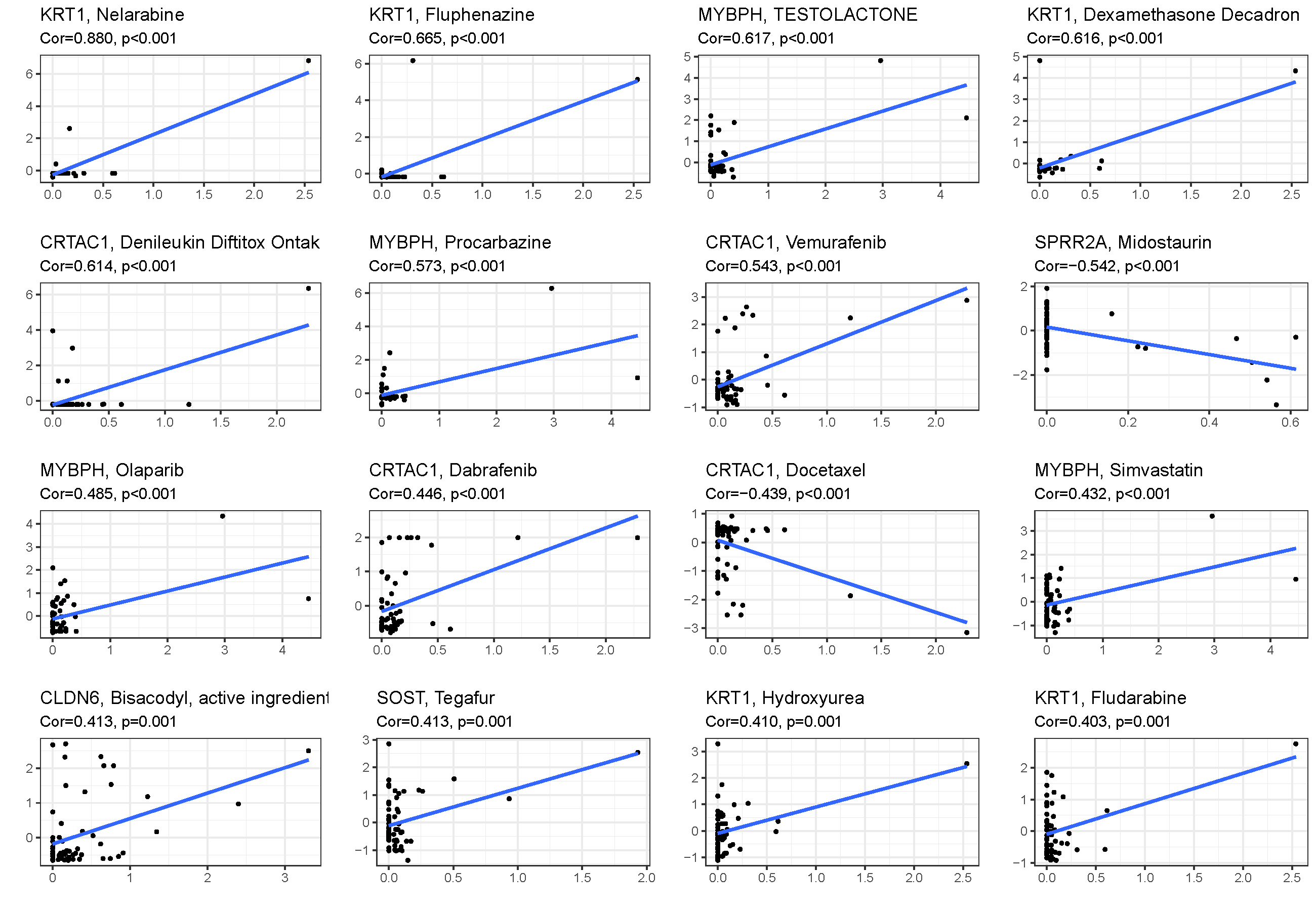


Figure.S2. Correlation analysis (KRT1, MYBPH, CRTAC1, SPRR2A, CLDN6, and SOST) in prognostic signatures and drug sensitivity.

# Appendix 6

## **hub genes analysis**

**Table 4. Hub genes.**

| name | Betweenness | Closeness | Degree | Network |
| --- | --- | --- | --- | --- |
| APRT | 828.704621 | 0.493087558 | 40 | 27.40412118 |
| ADSL | 873.3638309 | 0.486363636 | 38 | 25.51229816 |
| AK3 | 585.3028189 | 0.447698745 | 35 | 20.21861471 |
| DCK | 443.7513094 | 0.443983402 | 33 | 18.00123868 |
| ITPA | 661.2044784 | 0.469298246 | 31 | 20.58919999 |
| ADK | 136.6726562 | 0.416342412 | 30 | 15.74915622 |
| ENTPD8 | 465.2935566 | 0.490825688 | 30 | 22.47399125 |
| ENTPD1 | 515.8025815 | 0.493087558 | 28 | 18.51930358 |
| GMPS | 4189.276682 | 0.502347418 | 27 | 18.03359053 |
| NT5E | 73.15933993 | 0.453389831 | 26 | 20.79567977 |
| AMPD2 | 100.1294418 | 0.413127413 | 25 | 15.89612323 |
| RRM1 | 1670.585483 | 0.426294821 | 25 | 14.88244511 |
| POLR1C | 1783.945674 | 0.391941392 | 25 | 24.08333333 |
| HPRT1 | 476.4480227 | 0.453389831 | 24 | 16.97502012 |
| GUK1 | 639.9504829 | 0.479820628 | 24 | 13.87719621 |
| POLR2F | 13.2790071 | 0.300561798 | 24 | 23.8875 |
| POLR2L | 13.2790071 | 0.300561798 | 24 | 23.8875 |
| NT5M | 83.96301638 | 0.445833333 | 23 | 16.50016503 |
| NT5C3B | 72.88730533 | 0.445833333 | 23 | 17.65688452 |
| POLR2K | 10.06941114 | 0.299719888 | 23 | 22.38361653 |
| POLR3A | 11.28468476 | 0.299719888 | 23 | 22.31636254 |
| POLR2E | 10.06941114 | 0.299719888 | 23 | 22.38361653 |
| POLR2H | 10.06941114 | 0.299719888 | 23 | 22.38361653 |
| NT5C3A | 69.32060719 | 0.443983402 | 22 | 16.28571698 |
| ATIC | 80.62507377 | 0.443983402 | 22 | 17.05866013 |
| NT5C1A | 52.40209433 | 0.44214876 | 21 | 16.23631222 |
| NT5C | 63.43906497 | 0.44214876 | 21 | 15.40056561 |
| PKM | 146.007316 | 0.413127413 | 21 | 13.03073617 |
| ENPP1 | 245.5960189 | 0.449579832 | 21 | 12.9451693 |
| RRM2 | 638.803852 | 0.413127413 | 21 | 12.58903273 |
| CANT1 | 133.0017194 | 0.44214876 | 21 | 12.48277698 |
| POLR1B | 1110.192982 | 0.386281588 | 21 | 18.55035751 |
| POLR1D | 5.41513209 | 0.298050139 | 21 | 20.012629 |
| ENPP3 | 95.44019551 | 0.443983402 | 20 | 13.45562661 |
| PKLR | 47.85437997 | 0.409961686 | 20 | 13.30968354 |
| POLR3B | 4.607245532 | 0.297222222 | 20 | 18.76004042 |
| POLR3F | 5.161502387 | 0.297222222 | 20 | 18.60008194 |
| POLR3K | 4.921156621 | 0.297222222 | 20 | 18.41748836 |
| NME6 | 53.12487633 | 0.411538462 | 19 | 12.76152761 |
| POLR2G | 4.986249861 | 0.296398892 | 19 | 17.26143791 |
| POLR3H | 3.538197913 | 0.296398892 | 19 | 17.60947712 |
| POLR3C | 3.345535021 | 0.296398892 | 19 | 17.90918803 |
| NME1 | 172.3314682 | 0.409961686 | 18 | 11.91112025 |
| NUDT16 | 343.2213179 | 0.387681159 | 18 | 9.66512605 |
| POLR2D | 3.647460872 | 0.29558011 | 18 | 16.56092437 |
| POLR2I | 4.130996781 | 0.29558011 | 18 | 16.46250808 |
| ADCY5 | 54.8321709 | 0.346278317 | 17 | 7.756988844 |
| ENTPD6 | 63.42280905 | 0.43495935 | 17 | 11.75320513 |
| POLR1A | 775.7990842 | 0.380782918 | 17 | 14.40865385 |
| POLR3E | 1.632561883 | 0.29476584 | 17 | 16.25 |
| PDE1B | 106.1939845 | 0.393382353 | 16 | 7.112770563 |
| PDE1A | 165.5105741 | 0.389090909 | 16 | 8.22987013 |
| ADCY9 | 27.34604456 | 0.309248555 | 16 | 7.301656677 |
| AK4 | 61.65932186 | 0.408396947 | 16 | 7.141025641 |
| AK2 | 49.63971482 | 0.402255639 | 16 | 7.366666667 |
| NME2 | 41.79339148 | 0.406844106 | 16 | 10.68018648 |
| PDE1C | 84.9880413 | 0.387681159 | 15 | 6.693722944 |
| ADCY2 | 20.67827977 | 0.308357349 | 15 | 6.977414252 |
| ADCY4 | 20.67827977 | 0.308357349 | 15 | 6.977414252 |
| NME3 | 35.70124539 | 0.40530303 | 15 | 10.42707293 |
| POLR2J | 1.438383838 | 0.293150685 | 15 | 14.28571429 |
| ADA | 12.02932406 | 0.421259843 | 14 | 12.15384615 |
| PDE4B | 130.797718 | 0.387681159 | 14 | 5.846153846 |
| HDDC3 | 96.97871391 | 0.380782918 | 14 | 10.46153846 |
| POLR1E | 0.544047619 | 0.292349727 | 14 | 13.38461538 |
| POLR3G | 0.5 | 0.292349727 | 14 | 13.67832168 |
| PDE2A | 84.9880413 | 0.384892086 | 13 | 2.75 |
| IMPDH1 | 26.72361168 | 0.413127413 | 13 | 9.555555556 |
| POLA1 | 191.3163864 | 0.314705882 | 13 | 11.91666667 |
| POLE2 | 191.3163864 | 0.314705882 | 13 | 11.91666667 |
| ADCY10 | 47.54291432 | 0.330246914 | 12 | 0.761363636 |
| PDE4D | 76.33674825 | 0.379432624 | 12 | 4.909090909 |
| PDE3A | 66.90306669 | 0.380782918 | 12 | 4.613636364 |
| AK7 | 22.15556907 | 0.396296296 | 12 | 6.909090909 |
| AK5 | 43.13951604 | 0.393382353 | 12 | 5.272727273 |
| POLA2 | 102.9925083 | 0.313782991 | 12 | 11.36363636 |
| POLE | 102.9925083 | 0.313782991 | 12 | 11.36363636 |
| PRIM1 | 137.7059623 | 0.313782991 | 12 | 10.32727273 |
| POLD1 | 102.9925083 | 0.313782991 | 12 | 11.36363636 |
| PRIM2 | 137.7059623 | 0.313782991 | 12 | 10.32727273 |
| POLR2J2 | 0 | 0.29076087 | 12 | 12 |
| POLR3GL | 0.142857143 | 0.29076087 | 12 | 11.81818182 |
| POLR2J3 | 0 | 0.29076087 | 12 | 12 |
| PDE7B | 61.61583116 | 0.378091873 | 11 | 4.2 |
| PDE7A | 66.29360702 | 0.382142857 | 11 | 4 |
| POLD3 | 0.444444444 | 0.245412844 | 11 | 10.8 |
| POLE3 | 0.444444444 | 0.245412844 | 11 | 10.8 |
| POLE4 | 0.444444444 | 0.245412844 | 11 | 10.8 |
| POLD2 | 0.444444444 | 0.245412844 | 11 | 10.8 |
| PDE4C | 50.27586126 | 0.376760563 | 10 | 3.333333333 |
| PPAT | 309.762446 | 0.403773585 | 10 | 7.111111111 |
| GART | 276.1303877 | 0.43495935 | 10 | 7.111111111 |
| PDE5A | 9.637765976 | 0.300561798 | 9 | 3.791666667 |
| PDE6C | 55.54992863 | 0.357859532 | 9 | 1.75 |
| DGUOK | 3.500964395 | 0.357859532 | 9 | 7 |
| NPR2 | 30.04417697 | 0.349673203 | 9 | 3 |
| NPR1 | 30.04417697 | 0.349673203 | 9 | 3 |
| POLD4 | 0 | 0.244292237 | 9 | 9 |
| PDE3B | 16.93935305 | 0.375438596 | 8 | 4.857142857 |
| GUCY2D | 27.22539617 | 0.34516129 | 7 | 1.333333333 |
| PFAS | 0 | 0.391941392 | 6 | 6 |
| PAICS | 0 | 0.391941392 | 6 | 6 |
| NUDT5 | 119.1185336 | 0.283819629 | 3 | 1.5 |
| PRPS2 | 117.3588787 | 0.296398892 | 3 | 1.5 |
| ADPRM | 0 | 0.242081448 | 2 | 2 |
| XDH | 212 | 0.314705882 | 2 | 0 |
| PRUNE | 0 | 0.29476584 | 2 | 2 |
| ALLC | 0 | 0.239910314 | 1 | 0 |

# Appendix 7

# **The gene expression profile and clinical characteristics**

**Table 5. The gene expression profile and clinical characteristics.**

| gene | Mean1 | Mean2 | logFC | pValue | fdr |
| --- | --- | --- | --- | --- | --- |
| PAEP | 1.798931373 | 0.084833663 | -4.406359477 | 5.62E-19 | 6.97E-18 |
| LINC01541 | 0.045627451 | 1.384264356 | 4.923073616 | 4.71E-18 | 5.02E-17 |
| GKN1 | 0.233159314 | 2.376395545 | 3.349387023 | 7.45E-11 | 2.97E-10 |
| LRTM1 | 0.050092647 | 1.170244059 | 4.546066781 | 2.36E-13 | 1.30E-12 |
| COL2A1 | 1.602362745 | 0.134878713 | -3.570466205 | 9.71E-06 | 2.07E-05 |
| BHMT | 1.882848529 | 27.26006683 | 3.855800251 | 1.19E-28 | 8.82E-27 |
| LINC00942 | 5.545228431 | 0.203543564 | -4.76783738 | 1.91E-06 | 4.46E-06 |
| SPINK6 | 5.291398039 | 0.081830693 | -6.014863066 | 8.92E-07 | 2.17E-06 |
| SPRR2E | 39.71209951 | 0.958987624 | -5.371922629 | 9.58E-07 | 2.32E-06 |
| TENM2 | 3.391495098 | 0.089106931 | -5.250239949 | 6.95E-22 | 1.40E-20 |
| THRSP | 1.813728922 | 0.088734158 | -4.353325458 | 0.029459183 | 0.038403531 |
| KRT6C | 35.93815245 | 0.668114851 | -5.749276304 | 7.80E-15 | 5.25E-14 |
| MSMB | 2.460891176 | 20.38297327 | 3.050111746 | 4.23E-11 | 1.74E-10 |
| KRT14 | 934.699398 | 81.91027178 | -3.512386178 | 3.67E-12 | 1.72E-11 |
| LRRC38 | 1.17312549 | 0.089291584 | -3.715689332 | 3.70E-05 | 7.29E-05 |
| LINC00973 | 1.121172059 | 0.014561386 | -6.26671619 | 1.13E-18 | 1.33E-17 |
| AC079466.1 | 0.140646569 | 2.855989604 | 4.343844467 | 1.19E-11 | 5.24E-11 |
| KRT6B | 162.9860716 | 2.592054455 | -5.974508842 | 1.05E-18 | 1.25E-17 |
| PI3 | 954.7646858 | 73.55469257 | -3.698255917 | 3.55E-16 | 2.92E-15 |
| LINC00165 | 1.224313235 | 0.077159901 | -3.98797761 | 3.40E-12 | 1.60E-11 |
| LEAP2 | 0.92639902 | 7.748729208 | 3.064254096 | 1.81E-26 | 8.39E-25 |
| HKDC1 | 1.314255392 | 0.151984653 | -3.112248093 | 7.68E-12 | 3.47E-11 |
| KLK5 | 25.31973137 | 2.499563861 | -3.340513807 | 2.56E-17 | 2.46E-16 |
| AL161431.1 | 9.144605392 | 0.663481683 | -3.784792374 | 6.81E-22 | 1.37E-20 |
| SPRR2F | 4.417575 | 0.372728218 | -3.567058679 | 0.000156556 | 0.000282451 |
| CLDN6 | 4.038860294 | 0.449620297 | -3.167169174 | 2.88E-09 | 9.55E-09 |
| TGM1 | 12.01443725 | 0.927826238 | -3.694770621 | 3.11E-15 | 2.21E-14 |
| KRT81 | 45.7234451 | 1.469460396 | -4.959575728 | 2.50E-19 | 3.26E-18 |
| CES1 | 57.55910245 | 4.701367327 | -3.613891785 | 2.33E-06 | 5.40E-06 |
| SAA1 | 91.88946618 | 8.224939604 | -3.481822496 | 7.25E-17 | 6.52E-16 |
| KLK13 | 3.23207549 | 0.256682178 | -3.654405859 | 0.000221799 | 0.000392337 |
| NFE4 | 1.225235784 | 0.120170792 | -3.349901216 | 0.000212769 | 0.000377536 |
| CTXND1 | 0.134192157 | 1.42415099 | 3.407729853 | 5.89E-19 | 7.29E-18 |
| TMPRSS11D | 2.826080882 | 0.211373267 | -3.740937922 | 0.000284564 | 0.000495724 |
| SOST | 4.680380392 | 0.059061386 | -6.308266761 | 1.96E-08 | 5.89E-08 |
| LTF | 59.29762696 | 3.861524257 | -3.940732031 | 0.000677072 | 0.001118684 |
| HMGA2 | 2.882510784 | 0.094885644 | -4.924992375 | 4.77E-30 | 5.03E-28 |
| B3GAT1-DT | 0.143316176 | 1.395761881 | 3.283779472 | 1.88E-28 | 1.33E-26 |
| SPRR2A | 72.03839853 | 1.478732673 | -5.60633294 | 1.23E-08 | 3.77E-08 |
| SPRR2D | 35.24302696 | 1.94025495 | -4.183019699 | 2.27E-07 | 5.94E-07 |
| LINC02154 | 6.038455882 | 0.347453465 | -4.119288005 | 3.50E-11 | 1.45E-10 |
| AC018978.1 | 1.113668627 | 0.065309406 | -4.091885426 | 1.40E-23 | 3.71E-22 |
| S100A7A | 6.274935784 | 0.38074505 | -4.042703511 | 2.95E-08 | 8.65E-08 |
| ANKRD1 | 1.471294608 | 0.165585149 | -3.15144097 | 6.22E-09 | 1.98E-08 |
| KRT6A | 844.2711922 | 61.01801238 | -3.790399397 | 2.85E-18 | 3.16E-17 |
| SH2D5 | 1.03117549 | 0.098708416 | -3.384972974 | 2.06E-25 | 7.95E-24 |
| HEPHL1 | 2.642261275 | 0.145417822 | -4.183497136 | 1.73E-12 | 8.47E-12 |
| BNC1 | 5.672532843 | 0.212063366 | -4.741425733 | 3.79E-23 | 9.33E-22 |
| CGB8 | 2.348606373 | 0.072608416 | -5.01552435 | 4.54E-08 | 1.29E-07 |
| MYOSLID | 1.145035294 | 0.070476733 | -4.022101216 | 1.19E-24 | 3.91E-23 |
| CRTAC1 | 1.486046078 | 29.69485149 | 4.320662061 | 1.33E-19 | 1.80E-18 |
| AL356433.1 | 0.404583333 | 3.433194554 | 3.08504282 | 2.61E-08 | 7.70E-08 |
| APCDD1L | 2.750064216 | 0.319597525 | -3.105137167 | 4.85E-24 | 1.43E-22 |
| RNVU1-31 | 1.605322549 | 0.176408416 | -3.185871906 | 0.018763446 | 0.025223423 |
| CCDC190 | 1.858604412 | 0.159322277 | -3.544199827 | 1.24E-06 | 2.98E-06 |
| DSG3 | 43.32325049 | 1.750038614 | -4.629682831 | 5.85E-20 | 8.37E-19 |
| MANCR | 0.983223039 | 0.118603465 | -3.051372559 | 2.74E-15 | 1.97E-14 |
| AC112721.1 | 1.08802549 | 0.129809406 | -3.067245527 | 3.87E-12 | 1.81E-11 |
| PICSAR | 8.587410294 | 0.551760891 | -3.960108016 | 7.55E-16 | 5.91E-15 |
| CDH17 | 3.012536275 | 0.081917327 | -5.200666167 | 2.89E-08 | 8.48E-08 |
| S100A3 | 58.57099608 | 5.75130495 | -3.348225189 | 3.71E-06 | 8.36E-06 |
| MYBPH | 3.708394118 | 0.052981188 | -6.129170571 | 1.24E-06 | 2.97E-06 |
| CRH | 8.091680882 | 80.08474208 | 3.307016074 | 7.17E-20 | 1.01E-18 |
| KRT31 | 7.759623529 | 0.831666337 | -3.221909917 | 0.003239527 | 0.004870708 |
| CXCL5 | 7.852669118 | 0.90104604 | -3.12351038 | 2.16E-17 | 2.10E-16 |
| UTS2 | 1.189780392 | 0.148067327 | -3.006370079 | 0.002453485 | 0.003752676 |
| RN7SL3 | 10.3180299 | 0.527804455 | -4.289020193 | 0.019590819 | 0.026257638 |
| SPRR1B | 231.4783377 | 28.42689653 | -3.025548681 | 0.000106607 | 0.000197358 |
| KRT5 | 1122.089218 | 119.8022975 | -3.227459909 | 1.29E-17 | 1.29E-16 |
| AC012307.1 | 0.317427451 | 3.578739604 | 3.494952772 | 1.47E-16 | 1.27E-15 |
| NMRAL2P | 3.992963725 | 0.273894059 | -3.865770085 | 3.66E-15 | 2.57E-14 |
| BTBD16 | 4.351156863 | 34.8907698 | 3.003374495 | 1.00E-31 | 1.57E-29 |
| PPBP | 1.598137255 | 0.140111386 | -3.511745212 | 1.70E-07 | 4.53E-07 |
| IL36G | 5.212036275 | 0.24299901 | -4.422824784 | 7.05E-05 | 0.00013382 |
| KRTAP5-10 | 0.177505882 | 1.919980693 | 3.435153064 | 8.00E-34 | 1.98E-31 |
| CYP1A2 | 0.170655392 | 15.69288564 | 6.522880853 | 1.30E-06 | 3.10E-06 |
| CCER2 | 0.404537745 | 10.6023604 | 4.711967358 | 1.57E-10 | 6.05E-10 |
| CGB5 | 7.374586275 | 0.232810891 | -4.985331659 | 0.006302074 | 0.009068478 |
| ZFP42 | 1.123379902 | 0.059144059 | -4.24746882 | 1.71E-05 | 3.52E-05 |
| STYXL2 | 0.112422059 | 1.825892574 | 4.021604841 | 0.006971236 | 0.009975397 |
| KRT1 | 61.08407745 | 3.032706931 | -4.332118374 | 1.49E-05 | 3.09E-05 |
| LINC00709 | 0.091319118 | 1.14405396 | 3.64709437 | 2.87E-21 | 5.12E-20 |
| SCGB1A1 | 12.5427902 | 1.327385644 | -3.240198836 | 0.003848236 | 0.005719928 |
| SPIB | 5.16347549 | 0.423077723 | -3.609347831 | 2.54E-05 | 5.12E-05 |
| KLK7 | 7.411704412 | 0.471007426 | -3.975983636 | 1.94E-13 | 1.08E-12 |
| AC078880.3 | 0.144590196 | 1.98500297 | 3.779099527 | 2.24E-20 | 3.47E-19 |
| LINC00930 | 0.394121569 | 3.560245545 | 3.175264136 | 7.06E-37 | 4.72E-34 |
| FDCSP | 126.4176127 | 7.036363861 | -4.167223578 | 1.50E-05 | 3.11E-05 |
| KRT34 | 2.004078922 | 0.087284158 | -4.521075677 | 8.77E-14 | 5.12E-13 |
| AMTN | 3.602858824 | 0.381191089 | -3.240555822 | 1.66E-05 | 3.43E-05 |
| KLK3 | 0.100397059 | 2.206938119 | 4.458257267 | 5.54E-07 | 1.38E-06 |
| SPRR2G | 17.35531275 | 0.513058911 | -5.080109063 | 3.51E-07 | 8.99E-07 |
| STK32A-AS1 | 0.210547549 | 1.906823762 | 3.178953523 | 4.87E-18 | 5.18E-17 |
| FGFBP2 | 1.388912745 | 0.093777228 | -3.888574528 | 4.24E-05 | 8.29E-05 |
| KLK10 | 6.902260294 | 0.261779703 | -4.720643733 | 1.14E-16 | 1.00E-15 |
| KRT75 | 3.554111275 | 0.229133168 | -3.955230634 | 3.89E-16 | 3.19E-15 |
| AC083967.1 | 1.482943137 | 0.02840297 | -5.706277659 | 3.97E-10 | 1.45E-09 |

**Appendix 8**

**6 risk PRGs**

**Table 6. 6 risk PRGs.**

| id | CLDN6 | CES1 | SOST | SPRR2A | CRTAC1 | MYBPH | CGB5 | KRT1 | risk |
| --- | --- | --- | --- | --- | --- | --- | --- | --- | --- |
| TCGA-ZF-A9RN | 2.59111 | 2.935892 | 2.54112 | 2.429008 | 3.295386 | 3.257082 | 2.83786 | 8.78884 | high |
| TCGA-CF-A1HR | 2.568406 | 4.082216 | 2.546338 | 2.382946 | 7.716706 | 3.237093 | 2.83786 | 2.685695 | low |
| TCGA-XF-A8HH | 2.637824 | 4.902328 | 2.55736 | 2.645143 | 3.615747 | 3.232687 | 2.881544 | 2.693166 | low |
| TCGA-ZF-AA4W | 2.796482 | 2.981199 | 2.574683 | 7.02559 | 3.900324 | 3.398852 | 3.203752 | 3.990756 | high |
| TCGA-XF-A9SU | 2.568406 | 4.835142 | 2.497418 | 3.143062 | 3.746007 | 3.210315 | 3.970005 | 3.067613 | high |
| TCGA-DK-A3IQ | 2.666685 | 6.466353 | 2.572003 | 2.417158 | 3.630695 | 3.197081 | 2.930173 | 2.826405 | high |
| TCGA-FD-A6TK | 3.134578 | 4.451554 | 2.657392 | 3.039233 | 3.661161 | 3.238623 | 5.346553 | 2.701309 | high |
| TCGA-DK-A1A7 | 2.613797 | 3.802493 | 2.507371 | 2.558983 | 8.463898 | 3.176865 | 2.866393 | 3.414276 | low |
| TCGA-CU-A5W6 | 2.609501 | 3.189658 | 2.510851 | 4.921477 | 3.912806 | 3.250252 | 3.30436 | 4.359507 | high |
| TCGA-DK-A3X1 | 2.685392 | 3.204895 | 2.561486 | 2.996316 | 3.303946 | 3.224808 | 2.911962 | 3.119671 | low |
| TCGA-XF-A9ST | 11.142175 | 4.21892 | 2.497418 | 2.735936 | 6.313235 | 3.176865 | 11.428677 | 2.615272 | high |
| TCGA-CF-A9FM | 2.590954 | 3.91047 | 2.497418 | 2.382946 | 8.914129 | 3.176865 | 2.83786 | 2.628104 | low |
| TCGA-BT-A20V | 2.681581 | 2.954954 | 2.534963 | 2.382946 | 5.126931 | 3.558452 | 2.83786 | 2.785941 | low |
| TCGA-GV-A3QF | 2.608271 | 3.004842 | 2.497418 | 2.423769 | 3.284267 | 3.176865 | 2.83786 | 2.615272 | low |
| TCGA-DK-A2I4 | 2.636616 | 4.005226 | 2.584844 | 2.438939 | 4.263747 | 3.352297 | 2.889207 | 2.699158 | low |
| TCGA-DK-A6B2 | 2.587063 | 5.166738 | 2.497418 | 2.627711 | 3.613837 | 3.22134 | 3.004479 | 2.615272 | high |
| TCGA-DK-A1AG | 2.585815 | 3.12188 | 2.497418 | 3.610652 | 6.081835 | 3.176865 | 2.870195 | 2.634912 | low |
| TCGA-XF-A9SZ | 2.592974 | 3.850542 | 3.501327 | 3.823096 | 3.343338 | 3.263701 | 2.83786 | 2.670011 | low |
| TCGA-E7-A519 | 2.598863 | 3.755841 | 2.497418 | 2.613401 | 8.70937 | 3.483223 | 3.101884 | 2.632521 | low |
| TCGA-2F-A9KO | 2.642345 | 4.477532 | 2.513655 | 2.529465 | 3.595765 | 3.20682 | 2.973455 | 4.858913 | high |
| TCGA-ZF-A9R1 | 2.673782 | 4.879361 | 2.511519 | 2.978591 | 5.224199 | 3.176865 | 2.955768 | 2.77731 | low |
| TCGA-4Z-AA7S | 2.568406 | 3.084984 | 2.497418 | 2.504976 | 7.5012 | 3.176865 | 2.83786 | 2.638554 | low |
| TCGA-BL-A13J | 2.585194 | 3.831447 | 2.53489 | 4.571845 | 3.335107 | 3.191562 | 2.881355 | 7.534836 | high |
| TCGA-DK-A3IK | 2.61487 | 4.685786 | 2.669212 | 3.172061 | 7.189603 | 3.176865 | 3.005012 | 3.664037 | low |
| TCGA-C4-A0F1 | 2.62083 | 3.968237 | 2.597439 | 7.362665 | 3.667455 | 3.219024 | 4.050164 | 5.62869 | high |
| TCGA-DK-A3IS | 2.568406 | 2.987355 | 2.497418 | 2.441831 | 5.070908 | 3.194345 | 2.83786 | 2.99327 | low |
| TCGA-UY-A9PH | 2.568406 | 3.771242 | 2.497418 | 2.809528 | 3.552638 | 3.176865 | 3.081739 | 4.61873 | high |
| TCGA-E5-A4TZ | 2.627376 | 3.130469 | 2.497418 | 7.041938 | 3.298816 | 3.212447 | 2.893096 | 8.360981 | high |
| TCGA-DK-A3WW | 2.568406 | 2.94935 | 2.533517 | 3.778437 | 3.302549 | 3.243397 | 2.872822 | 4.413036 | low |
| TCGA-K4-A5RH | 2.722583 | 3.596965 | 3.028704 | 2.447834 | 4.442098 | 3.189647 | 2.858023 | 2.615272 | low |
| TCGA-ZF-AA56 | 2.588153 | 9.986179 | 3.139273 | 7.068151 | 5.136537 | 3.400614 | 3.537139 | 4.580508 | high |
| TCGA-UY-A8OB | 2.595301 | 2.939752 | 2.514855 | 6.013167 | 3.284267 | 3.240534 | 3.028316 | 2.864786 | low |
| TCGA-YC-A9TC | 2.643248 | 3.398558 | 2.746373 | 2.459282 | 3.357353 | 3.434489 | 3.271795 | 2.787504 | low |
| TCGA-CF-A7I0 | 2.613184 | 4.342389 | 2.497418 | 2.515806 | 6.580552 | 3.176865 | 2.879949 | 2.640809 | low |
| TCGA-K4-A5RJ | 2.594836 | 8.667655 | 2.531544 | 3.633735 | 3.42144 | 4.937628 | 2.83786 | 2.615272 | high |
| TCGA-YF-AA3L | 2.675404 | 3.636194 | 2.497418 | 2.382946 | 8.289827 | 3.176865 | 2.83786 | 2.676478 | low |
| TCGA-CF-A3MI | 2.625704 | 3.082413 | 2.497418 | 2.667481 | 3.817576 | 3.222882 | 2.909119 | 2.647799 | low |
| TCGA-DK-A6B0 | 2.586907 | 3.217737 | 2.497418 | 2.42059 | 8.833655 | 3.199033 | 3.033824 | 2.615272 | low |
| TCGA-DK-A6B1 | 2.658961 | 3.380937 | 2.497418 | 3.113522 | 3.329044 | 3.176865 | 2.83786 | 2.748762 | low |
| TCGA-G2-AA3C | 2.642646 | 9.286003 | 2.497418 | 2.662236 | 3.284267 | 12.293386 | 2.974273 | 2.862473 | high |
| TCGA-XF-AAN3 | 2.599946 | 10.172579 | 5.788218 | 4.484128 | 3.541602 | 3.205265 | 3.206553 | 2.689677 | high |
| TCGA-CU-A0YO | 2.632987 | 5.422679 | 2.497418 | 2.864512 | 4.163622 | 3.176865 | 2.83786 | 3.947997 | high |
| TCGA-BT-A3PJ | 2.583162 | 2.955548 | 2.497418 | 4.539702 | 3.313077 | 3.176865 | 3.29164 | 2.824455 | low |
| TCGA-CF-A47Y | 2.585971 | 3.479802 | 2.497418 | 2.382946 | 12.09924 | 3.176865 | 2.870633 | 2.615272 | low |
| TCGA-FD-A62O | 2.568406 | 3.981929 | 2.497418 | 4.406247 | 6.863521 | 3.20332 | 2.918906 | 2.974133 | low |
| TCGA-BL-A0C8 | 2.739235 | 3.221817 | 2.497418 | 2.779548 | 3.451633 | 3.197935 | 4.797401 | 2.645703 | high |
| TCGA-E7-A7DV | 2.774909 | 2.999346 | 2.633314 | 2.382946 | 3.291949 | 6.650431 | 2.895826 | 2.624179 | high |
| TCGA-DK-A3WY | 2.587842 | 5.206177 | 2.510049 | 2.382946 | 4.330893 | 3.376871 | 2.83786 | 2.615272 | low |
| TCGA-BT-A20N | 2.668018 | 3.491158 | 2.593799 | 3.360959 | 3.505976 | 3.176865 | 2.975906 | 3.305848 | low |
| TCGA-G2-A2ES | 2.598709 | 3.208481 | 2.583831 | 11.978168 | 3.299127 | 3.176865 | 2.83786 | 3.156971 | high |
| TCGA-GC-A3YS | 2.588776 | 4.083973 | 2.835747 | 2.612665 | 3.362146 | 3.225193 | 2.83786 | 3.284329 | low |
| TCGA-FD-A43X | 2.618082 | 3.004727 | 2.497418 | 2.382946 | 10.761213 | 3.294049 | 2.869026 | 2.689195 | low |
| TCGA-XF-A9SM | 3.358259 | 3.517705 | 2.715255 | 4.652393 | 3.338942 | 3.333982 | 3.540872 | 3.713449 | high |
| TCGA-DK-A6AV | 2.649851 | 3.521987 | 2.497418 | 2.382946 | 6.20826 | 3.306096 | 2.83786 | 2.631008 | low |
| TCGA-GU-A42Q | 2.617929 | 10.607328 | 8.91209 | 4.63183 | 3.440053 | 3.176865 | 2.83786 | 4.339014 | high |
| TCGA-C4-A0F7 | 2.628591 | 4.277104 | 2.497418 | 6.539059 | 3.838924 | 3.191606 | 4.442799 | 11.152529 | high |
| TCGA-CF-A47W | 2.568406 | 3.441493 | 2.497418 | 2.382946 | 7.840375 | 3.197472 | 3.021601 | 2.625066 | low |
| TCGA-XF-AAMQ | 2.568406 | 3.315025 | 2.497418 | 2.951325 | 5.436563 | 3.176865 | 2.882414 | 2.744961 | low |
| TCGA-K4-A3WS | 2.60904 | 6.133829 | 2.562644 | 2.715661 | 4.356206 | 3.249491 | 3.053459 | 3.380994 | high |
| TCGA-C4-A0EZ | 8.151728 | 3.970061 | 2.521105 | 5.745263 | 7.651299 | 3.385408 | 2.83786 | 6.832573 | high |
| TCGA-CF-A47S | 2.597625 | 3.273693 | 2.497418 | 3.054504 | 11.311606 | 3.176865 | 2.892089 | 2.615272 | low |
| TCGA-ZF-AA5P | 2.634349 | 6.615105 | 2.497418 | 3.153031 | 3.6709 | 3.331977 | 2.83786 | 2.736511 | high |
| TCGA-XF-A9SX | 2.629805 | 3.536745 | 2.576339 | 2.382946 | 3.344246 | 3.250442 | 11.963421 | 2.765559 | high |
| TCGA-FD-A3B8 | 2.677318 | 5.971799 | 2.497418 | 2.978669 | 3.609754 | 3.289396 | 2.83786 | 2.769752 | high |
| TCGA-CF-A27C | 2.584411 | 2.973255 | 2.507907 | 2.382946 | 6.171741 | 3.176865 | 2.867856 | 2.633403 | low |
| TCGA-XF-A9T4 | 2.652246 | 3.22004 | 2.50536 | 5.898599 | 3.32568 | 3.191606 | 4.497941 | 3.291116 | high |
| TCGA-HQ-A2OF | 5.64311 | 4.751231 | 2.522961 | 2.42328 | 3.576178 | 3.176865 | 2.83786 | 2.637676 | high |
| TCGA-DK-AA74 | 2.603651 | 5.305433 | 2.670638 | 6.441182 | 4.741013 | 3.2603 | 3.084019 | 4.172447 | high |
| TCGA-FD-A3SM | 2.672896 | 10.590784 | 2.751863 | 2.52271 | 3.336055 | 3.198252 | 2.871217 | 2.615272 | high |
| TCGA-KQ-A41P | 2.568406 | 4.255663 | 2.497418 | 3.008048 | 3.691775 | 3.320455 | 3.330447 | 3.01493 | high |
| TCGA-C4-A0F6 | 2.568406 | 4.241841 | 2.497418 | 2.510801 | 3.300217 | 3.215738 | 3.065553 | 9.080917 | high |
| TCGA-FD-A3B5 | 2.784014 | 2.992443 | 2.605314 | 10.282227 | 3.305187 | 3.228079 | 5.862781 | 8.668023 | high |
| TCGA-4Z-AA80 | 3.214986 | 3.096806 | 2.497418 | 2.616444 | 9.765324 | 3.206432 | 2.83786 | 2.670622 | low |
| TCGA-FD-A3B6 | 2.707206 | 3.019263 | 2.599318 | 2.896135 | 3.293044 | 3.282489 | 6.924859 | 2.665599 | high |
| TCGA-4Z-AA87 | 3.105903 | 3.961019 | 2.649936 | 3.31922 | 6.472416 | 3.2603 | 2.83786 | 2.615272 | low |
| TCGA-DK-A1A6 | 2.644601 | 3.101672 | 2.497418 | 4.0222 | 5.359919 | 3.213803 | 2.83786 | 2.615272 | low |
| TCGA-DK-A1AD | 2.568406 | 4.464368 | 2.611283 | 3.940036 | 4.456999 | 3.195127 | 2.83786 | 3.636445 | low |
| TCGA-FD-A5BS | 2.605193 | 5.787228 | 3.307886 | 2.382946 | 3.752087 | 3.504558 | 2.83786 | 2.696884 | high |
| TCGA-2F-A9KW | 5.440011 | 4.026 | 2.512855 | 2.763281 | 3.418845 | 3.233454 | 7.255833 | 2.642185 | high |
| TCGA-DK-AA6T | 2.591421 | 3.632909 | 2.497418 | 2.429737 | 5.791176 | 3.176865 | 2.83786 | 2.628357 | low |
| TCGA-4Z-AA84 | 3.037949 | 3.268911 | 2.555292 | 2.636509 | 3.306273 | 3.176865 | 2.921025 | 2.615272 | low |
| TCGA-FJ-A3Z9 | 2.828097 | 2.938547 | 2.497418 | 5.798549 | 7.163545 | 3.207403 | 6.0127 | 3.113981 | high |
| TCGA-CU-A0YR | 4.709616 | 3.793147 | 2.512454 | 2.382946 | 3.394272 | 3.337804 | 2.88111 | 2.667072 | high |
| TCGA-FD-A3B3 | 2.617318 | 5.137564 | 2.513389 | 8.785114 | 3.349532 | 3.23537 | 2.949964 | 9.147857 | high |
| TCGA-FD-A43N | 2.850147 | 3.976709 | 2.507371 | 4.941933 | 3.501739 | 3.231536 | 2.922295 | 2.910182 | high |
| TCGA-BL-A13I | 2.593253 | 10.530442 | 9.724249 | 2.622472 | 3.356176 | 3.283394 | 2.868934 | 3.805163 | high |
| TCGA-KQ-A41S | 2.658514 | 4.595148 | 2.497418 | 2.74477 | 6.149736 | 3.176865 | 2.83786 | 2.666704 | low |
| TCGA-4Z-AA86 | 2.629654 | 4.577328 | 2.746485 | 6.322202 | 3.484261 | 3.33835 | 7.677123 | 4.264232 | high |
| TCGA-E7-A5KF | 2.568406 | 2.940716 | 2.497418 | 2.420835 | 8.562076 | 3.264456 | 2.939544 | 2.615272 | low |
| TCGA-XF-AAN7 | 2.609194 | 5.023855 | 2.52402 | 2.382946 | 3.456055 | 3.273305 | 2.83786 | 2.661172 | low |
| TCGA-DK-AA6M | 2.989193 | 3.4022 | 2.573407 | 2.708472 | 3.298972 | 3.247779 | 4.832071 | 3.501518 | high |
| TCGA-CU-A3YL | 2.587998 | 3.302717 | 2.57213 | 2.382946 | 4.278927 | 3.200398 | 2.94511 | 2.701786 | low |
| TCGA-ZF-A9RD | 2.643097 | 3.527225 | 2.682343 | 10.948204 | 3.42676 | 3.222111 | 2.90798 | 7.803016 | high |
| TCGA-C4-A0F0 | 2.623117 | 3.406244 | 2.497418 | 3.135724 | 3.439341 | 3.176865 | 2.83786 | 3.358718 | low |
| TCGA-FD-A3SR | 2.626009 | 4.649389 | 2.510049 | 3.347003 | 3.421296 | 3.200203 | 2.83786 | 2.771448 | low |
| TCGA-DK-A6B5 | 2.646103 | 3.72056 | 2.517917 | 4.25014 | 3.439341 | 3.195909 | 2.8964 | 4.599789 | high |
| TCGA-HQ-A5ND | 2.610115 | 3.432574 | 2.497418 | 3.474928 | 3.345002 | 4.399957 | 2.83786 | 2.627219 | high |
| TCGA-DK-AA76 | 2.592508 | 3.385555 | 2.497418 | 4.394454 | 4.688837 | 3.176865 | 2.83786 | 3.120961 | low |
| TCGA-CF-A47V | 2.622355 | 3.073048 | 2.509246 | 2.382946 | 10.428302 | 3.176865 | 3.000201 | 2.79795 | low |
| TCGA-E7-A3Y1 | 2.58971 | 4.221511 | 2.497418 | 2.467918 | 5.030867 | 3.202346 | 2.87748 | 3.166958 | low |
| TCGA-ZF-AA53 | 2.647603 | 4.385659 | 3.215941 | 6.822361 | 3.310452 | 3.332707 | 2.887909 | 5.379113 | high |
| TCGA-ZF-A9R7 | 2.688465 | 3.534595 | 2.787396 | 2.561832 | 3.587623 | 3.213803 | 2.83786 | 2.981055 | low |
| TCGA-GV-A40G | 2.643548 | 2.929229 | 2.497418 | 2.808449 | 3.413932 | 3.195127 | 2.866539 | 2.666458 | low |
| TCGA-4Z-AA7M | 2.593285 | 3.324748 | 2.513655 | 3.191804 | 3.32062 | 3.176865 | 3.056427 | 2.629368 | low |
| TCGA-BT-A2LD | 8.049042 | 4.104076 | 2.497418 | 6.908489 | 3.510882 | 3.421113 | 3.29153 | 3.057718 | high |
| TCGA-4Z-AA82 | 2.589866 | 3.583773 | 2.552444 | 2.858731 | 5.696645 | 3.228079 | 3.486493 | 3.180153 | low |
| TCGA-FD-A62P | 2.709947 | 4.141918 | 2.676917 | 5.579798 | 3.487165 | 3.263512 | 2.83786 | 3.356862 | high |
| TCGA-DK-AA6U | 2.585815 | 3.220424 | 2.508711 | 3.984942 | 4.311229 | 3.218251 | 3.18719 | 3.353213 | low |
| TCGA-FD-A3N6 | 2.595456 | 6.230998 | 2.648247 | 10.897095 | 3.430631 | 3.318804 | 2.888342 | 11.731332 | high |
| TCGA-2F-A9KR | 2.568406 | 7.709177 | 2.511386 | 5.470087 | 4.033338 | 3.202541 | 2.954941 | 8.457513 | high |
| TCGA-CF-A3MF | 2.588776 | 2.979685 | 2.497418 | 2.464612 | 4.148187 | 3.176865 | 2.83786 | 2.626839 | low |
| TCGA-CU-A3KJ | 2.614257 | 3.070885 | 2.497418 | 4.777551 | 3.435492 | 3.387714 | 2.880819 | 2.886757 | low |
| TCGA-FD-A6TC | 2.641291 | 4.571628 | 2.874792 | 2.626152 | 3.512514 | 3.199033 | 2.939404 | 2.706907 | low |
| TCGA-XF-A8HB | 2.584723 | 3.057279 | 2.508041 | 2.479886 | 3.292262 | 3.176865 | 2.868295 | 2.651776 | low |
| TCGA-XF-AAN1 | 2.568406 | 3.267436 | 2.497418 | 2.817685 | 7.393035 | 3.214384 | 3.057973 | 2.667807 | low |
| TCGA-FJ-A871 | 3.035957 | 3.49208 | 2.520707 | 2.382946 | 3.837819 | 15.923566 | 3.198955 | 2.645681 | high |
| TCGA-UY-A9PA | 2.649402 | 3.209255 | 2.567396 | 2.491973 | 6.623646 | 3.209733 | 4.204486 | 2.895707 | low |
| TCGA-E7-A4IJ | 2.642345 | 3.098606 | 2.513655 | 2.482569 | 4.691985 | 3.176865 | 2.884441 | 2.629494 | low |
| TCGA-XF-AAMZ | 2.568406 | 3.269003 | 2.497418 | 2.382946 | 6.29848 | 3.176865 | 2.908692 | 2.835679 | low |
| TCGA-XF-AAMR | 2.595766 | 6.930625 | 2.846736 | 2.741453 | 4.21826 | 3.198643 | 2.871947 | 2.889953 | high |
| TCGA-BT-A42E | 2.647153 | 3.02871 | 2.701049 | 4.138401 | 3.338487 | 3.176865 | 2.867856 | 9.221881 | high |
| TCGA-K4-A6FZ | 2.644601 | 3.357501 | 2.537324 | 9.464154 | 5.591579 | 3.176865 | 2.950657 | 8.898526 | high |
| TCGA-DK-A1AF | 2.701128 | 4.099398 | 2.515255 | 5.80589 | 3.850696 | 3.723225 | 2.83786 | 3.076986 | high |
| TCGA-FD-A6TB | 2.716278 | 5.598621 | 2.509915 | 2.532941 | 3.428625 | 3.200008 | 2.83786 | 2.637048 | high |
| TCGA-GU-AATO | 2.661044 | 3.919783 | 2.497418 | 2.382946 | 3.935089 | 3.287718 | 3.109742 | 2.767713 | low |
| TCGA-BT-A20U | 2.598863 | 10.728366 | 3.931581 | 7.074633 | 3.53371 | 3.284358 | 3.326364 | 9.446188 | high |
| TCGA-DK-A1AA | 2.609962 | 3.845308 | 2.568293 | 2.68844 | 10.020058 | 3.22654 | 2.863901 | 2.764877 | low |
| TCGA-GV-A3JW | 2.584255 | 3.334133 | 2.497418 | 3.417011 | 8.409507 | 3.195909 | 2.83786 | 5.48633 | low |
| TCGA-FD-A5BZ | 2.641442 | 5.78459 | 3.610698 | 4.025975 | 6.754975 | 3.176865 | 2.928628 | 4.959422 | high |
| TCGA-ZF-A9R9 | 2.568406 | 4.56343 | 2.536013 | 2.382946 | 3.47816 | 3.247779 | 2.893527 | 2.697962 | low |
| TCGA-G2-AA3F | 2.732705 | 3.503399 | 2.497418 | 3.689414 | 4.413321 | 3.353019 | 2.83786 | 2.739643 | low |
| TCGA-ZF-AA5H | 2.883558 | 3.880928 | 2.515388 | 2.894371 | 3.439626 | 3.209927 | 8.758179 | 2.661788 | high |
| TCGA-XF-A8HD | 2.615635 | 3.488309 | 2.681283 | 2.382946 | 3.322769 | 3.251961 | 5.014167 | 2.624306 | high |
| TCGA-FD-A6TH | 6.414634 | 4.357185 | 2.532334 | 4.378618 | 3.666346 | 3.256893 | 2.960725 | 2.841147 | high |
| TCGA-4Z-AA7O | 2.597625 | 3.448599 | 2.497418 | 5.529256 | 9.713625 | 3.176865 | 3.138328 | 6.062265 | low |
| TCGA-DK-A6B6 | 2.568406 | 3.092453 | 2.530491 | 2.434952 | 8.190954 | 3.237858 | 2.83786 | 2.629873 | low |
| TCGA-E7-A6MF | 2.568406 | 5.615771 | 2.497418 | 2.382946 | 7.740032 | 3.176865 | 2.83786 | 2.626966 | low |
| TCGA-DK-AA6W | 7.556568 | 3.011449 | 2.512988 | 6.230358 | 3.554095 | 3.233837 | 2.83786 | 4.366913 | high |
| TCGA-GC-A3BM | 2.624944 | 5.760531 | 2.509781 | 2.459519 | 4.841373 | 3.176865 | 3.071053 | 5.975861 | high |
| TCGA-GU-A42P | 6.456648 | 2.973138 | 2.497418 | 4.260455 | 3.318776 | 3.210896 | 2.864488 | 2.655246 | high |
| TCGA-GV-A3QI | 2.582693 | 2.934804 | 2.506701 | 2.382946 | 8.658908 | 3.176865 | 2.891081 | 2.768846 | low |
| TCGA-XF-A9T3 | 2.797573 | 5.892681 | 2.619695 | 2.382946 | 3.528875 | 3.176865 | 11.795877 | 2.669889 | high |
| TCGA-E7-A8O7 | 2.600255 | 3.354067 | 2.497418 | 2.824989 | 7.986161 | 3.196104 | 2.83786 | 2.624306 | low |
| TCGA-DK-AA77 | 2.568406 | 3.050033 | 2.497418 | 2.382946 | 6.61608 | 3.203125 | 2.83786 | 2.779333 | low |
| TCGA-XF-AAML | 2.62768 | 3.008833 | 2.497418 | 5.248843 | 4.346002 | 3.247779 | 3.233703 | 2.76044 | low |
| TCGA-2F-A9KQ | 2.607964 | 3.927678 | 2.497418 | 2.46272 | 8.375405 | 3.176865 | 3.014987 | 2.754843 | low |
| TCGA-CF-A3MH | 2.568406 | 2.981083 | 2.507907 | 2.382946 | 3.755585 | 3.1963 | 2.897978 | 2.624433 | low |
| TCGA-XF-A8HC | 2.568406 | 2.996705 | 2.497418 | 3.732009 | 5.591289 | 3.176865 | 2.83786 | 2.644558 | low |
| TCGA-ZF-AA4T | 2.68466 | 3.138487 | 2.512988 | 2.382946 | 3.493506 | 3.176865 | 2.83786 | 2.628862 | low |
| TCGA-FD-A6TI | 2.710668 | 4.484987 | 2.497418 | 2.382946 | 7.401826 | 3.212253 | 2.83786 | 2.623672 | low |
| TCGA-H4-A2HO | 2.568406 | 4.068642 | 2.497418 | 2.544976 | 3.412339 | 3.22654 | 2.9148 | 2.782473 | low |
| TCGA-CF-A47X | 2.568406 | 3.136025 | 2.497418 | 2.382946 | 10.615397 | 3.176865 | 2.83786 | 2.623037 | low |
| TCGA-XF-A9SP | 2.701563 | 6.37831 | 2.497418 | 2.600731 | 3.766608 | 3.258219 | 8.396185 | 2.615272 | high |
| TCGA-K4-A4AC | 2.568406 | 3.657062 | 2.497418 | 5.27634 | 3.328586 | 3.176865 | 2.83786 | 9.661293 | high |
| TCGA-BL-A5ZZ | 3.052519 | 4.833828 | 2.509648 | 3.462452 | 3.554492 | 4.098768 | 2.940519 | 4.074202 | high |
| TCGA-S5-AA26 | 2.568406 | 3.887684 | 2.497418 | 3.519184 | 4.335972 | 3.176865 | 2.83786 | 6.023216 | high |
| TCGA-E7-A677 | 2.665499 | 3.464889 | 2.505494 | 3.204359 | 8.266434 | 3.176865 | 3.096245 | 2.955487 | low |
| TCGA-DK-A1AC | 2.568406 | 3.585127 | 2.497418 | 2.414332 | 4.149003 | 3.249491 | 2.866539 | 2.623926 | low |
| TCGA-GU-A766 | 2.622812 | 4.273245 | 2.497418 | 8.776012 | 3.438202 | 3.538517 | 2.905415 | 4.883463 | high |
| TCGA-DK-A2HX | 2.609655 | 5.988467 | 2.515388 | 3.08869 | 3.81612 | 3.242634 | 2.83786 | 2.785047 | high |
| TCGA-UY-A8OC | 2.655383 | 4.054819 | 2.497418 | 5.501407 | 3.608348 | 3.229616 | 7.42492 | 2.689074 | high |
| TCGA-FD-A5BR | 2.999306 | 5.34726 | 2.512588 | 3.684346 | 6.883653 | 3.313288 | 2.965118 | 4.469951 | high |
| TCGA-DK-AA6S | 2.617318 | 4.393506 | 2.719613 | 2.52733 | 3.467007 | 3.23537 | 2.83786 | 4.087615 | high |
| TCGA-4Z-AA7Q | 2.568406 | 3.688763 | 2.514189 | 2.667984 | 3.284267 | 3.297392 | 2.83786 | 2.658583 | low |
| TCGA-HQ-A2OE | 2.590021 | 3.566103 | 2.497418 | 2.588684 | 7.655862 | 3.176865 | 3.166496 | 3.084171 | low |
| TCGA-XF-AAMX | 2.59344 | 3.438287 | 2.723045 | 2.52969 | 3.320774 | 3.20682 | 2.83786 | 2.657471 | low |
| TCGA-LT-A5Z6 | 2.615941 | 3.253333 | 2.497418 | 3.245624 | 4.11454 | 3.20546 | 2.83786 | 4.619501 | low |
| TCGA-YC-A89H | 2.666092 | 3.443813 | 2.622282 | 3.0199 | 3.361099 | 3.224616 | 2.911678 | 2.954117 | low |
| TCGA-E7-A8O8 | 2.568406 | 3.044074 | 2.497418 | 3.734646 | 3.383833 | 3.176865 | 3.124596 | 2.761465 | low |
| TCGA-GC-A3OO | 2.588932 | 5.829563 | 2.497418 | 2.504518 | 3.619434 | 3.365788 | 2.876171 | 3.440505 | high |
| TCGA-DK-AA75 | 3.203115 | 3.217833 | 2.497418 | 4.769689 | 3.856057 | 3.306096 | 3.033955 | 3.114327 | low |
| TCGA-BT-A2LB | 2.800162 | 5.130414 | 2.685988 | 4.064952 | 6.002666 | 3.195518 | 3.373284 | 3.67878 | high |
| TCGA-ZF-AA54 | 2.568406 | 3.490081 | 2.497418 | 2.382946 | 3.728216 | 3.204682 | 2.83786 | 2.729058 | low |
| TCGA-FT-A3EE | 2.664758 | 3.711467 | 2.497418 | 2.470509 | 3.38339 | 3.176865 | 3.120155 | 2.664248 | low |
| TCGA-FJ-A3ZF | 3.004697 | 3.044737 | 2.505762 | 2.409155 | 3.462383 | 3.176865 | 2.861846 | 2.636923 | low |
| TCGA-DK-A3X2 | 2.568406 | 3.011449 | 2.509112 | 4.299155 | 3.403332 | 3.176865 | 2.83786 | 8.548702 | high |
| TCGA-XF-AAME | 2.770059 | 6.706755 | 2.520441 | 8.673055 | 3.990707 | 3.240152 | 3.056556 | 4.104116 | high |
| TCGA-ZF-AA4R | 3.38855 | 3.79723 | 3.104935 | 5.333081 | 4.072275 | 3.176865 | 3.071053 | 5.882551 | high |
| TCGA-GV-A3JZ | 3.234644 | 4.436255 | 2.497418 | 2.382946 | 4.87143 | 4.991119 | 3.394199 | 2.630756 | high |
| TCGA-DK-A2I2 | 2.660449 | 3.901032 | 2.497418 | 8.873302 | 3.434064 | 3.490641 | 2.872968 | 7.32866 | high |
| TCGA-K4-A83P | 2.640237 | 5.194254 | 2.589518 | 2.456311 | 3.959108 | 3.283798 | 2.969907 | 2.656235 | high |
| TCGA-4Z-AA7Y | 2.632533 | 2.973958 | 2.525343 | 2.830573 | 11.154026 | 3.176865 | 2.917491 | 2.6988 | low |
| TCGA-E7-A7XN | 2.568406 | 3.035079 | 2.497418 | 7.270049 | 3.29445 | 3.201762 | 2.876753 | 5.282828 | high |
| TCGA-2F-A9KP | 2.644751 | 3.418379 | 2.497418 | 2.569143 | 5.091996 | 3.200203 | 2.83786 | 3.573027 | low |
| TCGA-UY-A78M | 2.568406 | 4.407739 | 3.860242 | 4.815683 | 3.509384 | 3.336531 | 2.83786 | 4.987441 | high |
| TCGA-FD-A6TD | 2.763523 | 4.237994 | 2.497418 | 3.822734 | 3.420143 | 3.201567 | 3.055137 | 2.727772 | low |
| TCGA-BT-A20Q | 2.629957 | 4.5989 | 2.777997 | 2.382946 | 4.160565 | 3.201762 | 2.914375 | 2.627092 | low |
| TCGA-ZF-AA51 | 2.598399 | 3.586052 | 2.64583 | 2.660517 | 4.270223 | 3.316416 | 2.83786 | 2.82033 | low |
| TCGA-GU-A42R | 2.568406 | 3.502409 | 2.497418 | 2.529914 | 4.029528 | 3.176865 | 2.873113 | 7.051611 | high |
| TCGA-PQ-A6FI | 2.64084 | 3.294803 | 2.506969 | 9.26151 | 3.375698 | 3.176865 | 2.865514 | 6.657306 | high |
| TCGA-ZF-AA4X | 2.608425 | 3.31058 | 2.497418 | 3.878306 | 4.938179 | 3.200983 | 2.83786 | 2.671234 | low |
| TCGA-FD-A5BU | 2.613338 | 9.931119 | 2.597063 | 9.086048 | 3.558853 | 3.203904 | 2.83786 | 6.713034 | high |
| TCGA-CF-A8HX | 2.568406 | 4.047946 | 2.510851 | 2.382946 | 7.853119 | 3.250442 | 3.154713 | 2.909675 | low |
| TCGA-XF-A9SK | 2.841947 | 4.190177 | 6.020151 | 2.433014 | 3.716799 | 3.176865 | 3.244313 | 2.926297 | high |
| TCGA-GD-A3OS | 2.869419 | 6.35942 | 2.580789 | 4.536688 | 6.989714 | 3.538517 | 3.30447 | 2.990037 | high |
| TCGA-FD-A6TG | 2.568406 | 5.930006 | 2.508443 | 2.450826 | 3.638493 | 3.236901 | 2.930453 | 2.843392 | high |
| TCGA-4Z-AA7N | 2.645802 | 5.20219 | 2.497418 | 2.735746 | 6.002359 | 3.48373 | 2.910399 | 3.170434 | low |
| TCGA-GV-A3QK | 2.582537 | 3.465831 | 2.515655 | 2.546971 | 8.41649 | 3.243778 | 2.864341 | 2.631261 | low |
| TCGA-K4-A5RI | 2.662976 | 10.775931 | 3.389929 | 5.846337 | 3.367671 | 3.176865 | 3.876688 | 3.608008 | high |
| TCGA-GV-A40E | 4.114078 | 3.129128 | 2.865059 | 4.956224 | 3.353447 | 3.458921 | 3.143931 | 2.713782 | high |
| TCGA-FD-A43Y | 2.568406 | 10.714994 | 4.35359 | 5.825754 | 3.45873 | 3.20682 | 6.800094 | 5.631112 | high |
| TCGA-DK-A3IV | 2.801931 | 3.395237 | 2.634291 | 2.919755 | 3.786829 | 3.21206 | 2.83786 | 2.640183 | low |
| TCGA-G2-A2EL | 3.600204 | 2.915057 | 2.497418 | 5.45286 | 4.604293 | 3.294049 | 2.891081 | 7.078624 | high |
| TCGA-E7-A541 | 2.568406 | 3.086054 | 2.497418 | 2.382946 | 3.56557 | 3.204682 | 2.88111 | 2.79983 | low |
| TCGA-XF-AAN2 | 2.601491 | 3.100404 | 2.518848 | 5.097461 | 3.300528 | 3.216318 | 4.108121 | 4.877093 | high |
| TCGA-LC-A66R | 2.637069 | 3.324484 | 2.497418 | 6.786567 | 3.334838 | 3.376514 | 2.964158 | 3.89624 | high |
| TCGA-ZF-A9RE | 2.638578 | 8.236162 | 2.497418 | 9.899787 | 3.352695 | 3.176865 | 2.83786 | 3.463383 | high |
| TCGA-UY-A9PE | 2.707928 | 4.687559 | 2.520839 | 4.1588 | 3.319391 | 3.176865 | 3.353446 | 4.222632 | high |
| TCGA-XF-AAMG | 3.319069 | 3.694185 | 2.526797 | 2.429129 | 4.253262 | 3.204098 | 3.621768 | 2.714963 | high |
| TCGA-GU-A763 | 2.596851 | 3.010426 | 2.497418 | 2.382946 | 13.319143 | 3.210896 | 2.890649 | 2.738832 | low |
| TCGA-G2-AA3D | 2.634349 | 3.208384 | 2.497418 | 4.034344 | 3.489235 | 3.229808 | 3.106878 | 2.665108 | low |
| TCGA-ZF-A9R4 | 2.587374 | 3.071967 | 2.509781 | 2.724349 | 4.197476 | 3.176865 | 2.907838 | 2.62608 | low |
| TCGA-E7-A97P | 2.568406 | 4.592986 | 2.538241 | 7.383692 | 3.325221 | 3.176865 | 2.83786 | 2.615272 | high |
| TCGA-5N-A9KM | 2.651049 | 6.862049 | 2.997281 | 2.382946 | 3.284267 | 3.176865 | 3.39574 | 2.627219 | high |
| TCGA-ZF-A9RC | 2.621288 | 5.206596 | 2.497418 | 2.632376 | 3.71668 | 3.27105 | 3.113221 | 3.637019 | high |
| TCGA-MV-A51V | 2.854231 | 3.616215 | 2.497418 | 2.47825 | 3.858895 | 3.233645 | 2.882414 | 2.615272 | low |
| TCGA-S5-A6DX | 2.595146 | 4.380039 | 2.497418 | 4.651434 | 3.707582 | 3.529793 | 3.113841 | 4.56809 | high |
| TCGA-FJ-A3ZE | 2.703157 | 4.878268 | 2.497418 | 2.755515 | 3.544535 | 3.176865 | 2.902416 | 4.048777 | high |
| TCGA-YC-A8S6 | 2.627832 | 4.536368 | 2.516853 | 2.443635 | 8.602274 | 3.447431 | 4.807703 | 3.504831 | low |
| TCGA-UY-A9PF | 3.260378 | 4.145535 | 2.519778 | 2.452378 | 7.168253 | 3.176865 | 2.901701 | 2.654008 | low |
| TCGA-GV-A3JV | 2.629198 | 3.513559 | 2.497418 | 2.414332 | 4.205718 | 3.176865 | 2.83786 | 2.615272 | low |
| TCGA-ZF-AA5N | 2.668166 | 3.209448 | 2.562644 | 4.520538 | 3.681799 | 3.296464 | 2.931575 | 3.034022 | low |
| TCGA-XF-A9T8 | 2.606117 | 3.536819 | 2.497418 | 11.033538 | 3.525239 | 3.287718 | 2.873259 | 2.6988 | high |
| TCGA-5N-A9KI | 2.783876 | 4.85697 | 2.534306 | 2.921402 | 3.511018 | 3.266908 | 3.040093 | 2.940233 | high |
| TCGA-CF-A3MG | 2.72058 | 4.502838 | 2.512187 | 2.516373 | 6.258133 | 3.176865 | 2.880094 | 2.615272 | low |
| TCGA-GC-A4ZW | 2.845126 | 3.103781 | 2.512454 | 2.382946 | 3.372581 | 3.176865 | 2.83786 | 2.641309 | low |
| TCGA-E7-A4XJ | 2.72644 | 3.869847 | 2.497418 | 3.308127 | 3.601298 | 3.176865 | 3.244767 | 2.749107 | low |
| TCGA-FD-A3NA | 2.599018 | 3.718134 | 2.527193 | 6.287647 | 4.917522 | 3.35356 | 4.017681 | 2.786835 | high |
| TCGA-DK-A3IT | 2.899491 | 4.546737 | 2.497418 | 2.491626 | 3.578395 | 3.176865 | 3.062089 | 3.455207 | high |
| TCGA-R3-A69X | 3.942562 | 4.801537 | 2.509246 | 2.382946 | 4.241852 | 3.262379 | 3.000067 | 2.91766 | high |
| TCGA-4Z-AA83 | 2.568406 | 4.528856 | 2.515655 | 2.691801 | 9.047761 | 3.210509 | 2.989722 | 2.708214 | low |
| TCGA-ZF-A9R0 | 2.645652 | 5.767426 | 2.497418 | 2.48699 | 5.338587 | 3.269356 | 2.83786 | 2.615272 | low |
| TCGA-GC-A3I6 | 2.568406 | 3.913691 | 2.556585 | 6.630375 | 3.633718 | 3.176865 | 2.971682 | 6.01639 | high |
| TCGA-XF-A9SH | 3.023362 | 4.584645 | 3.050748 | 2.755421 | 3.537193 | 3.176865 | 2.83786 | 2.654503 | high |
| TCGA-UY-A78L | 4.617179 | 6.582559 | 2.623267 | 8.820229 | 3.357053 | 3.197081 | 2.869318 | 4.700065 | high |
| TCGA-XF-AAN5 | 2.611037 | 3.333609 | 2.797811 | 3.155425 | 3.482184 | 3.227887 | 2.916925 | 2.686662 | low |
| TCGA-UY-A78P | 2.765612 | 4.055499 | 2.576721 | 5.637956 | 3.344548 | 3.644505 | 2.83786 | 3.127304 | high |
| TCGA-CF-A8HY | 2.592042 | 2.925945 | 2.497418 | 2.759638 | 5.488716 | 3.176865 | 3.045558 | 2.615272 | low |
| TCGA-CF-A9FL | 2.611957 | 4.123763 | 2.497418 | 2.427548 | 3.656455 | 3.203125 | 4.996559 | 2.676235 | high |
| TCGA-E7-A6MD | 2.612571 | 4.031759 | 2.51192 | 2.471802 | 4.475594 | 3.229808 | 3.175332 | 2.857202 | low |
| TCGA-CF-A9FF | 2.596696 | 3.981414 | 2.497418 | 3.843421 | 8.594406 | 3.176865 | 2.890505 | 2.708689 | low |
| TCGA-XF-AAMY | 2.984718 | 4.433508 | 2.497418 | 3.536885 | 4.045586 | 3.176865 | 3.018165 | 2.671845 | low |
| TCGA-G2-A3IE | 2.669942 | 3.843924 | 2.497418 | 3.018529 | 12.741504 | 3.197667 | 2.993626 | 2.960174 | low |
| TCGA-GU-A767 | 2.588776 | 4.737838 | 2.497418 | 3.239373 | 9.429174 | 3.176865 | 2.98486 | 3.245794 | low |
| TCGA-G2-A3IB | 2.599018 | 9.964685 | 4.584279 | 11.038869 | 3.616256 | 3.231536 | 3.103134 | 11.525553 | high |
| TCGA-CF-A1HS | 2.617318 | 2.982014 | 2.497418 | 10.232502 | 3.971032 | 3.176865 | 2.83786 | 10.953132 | high |
| TCGA-DK-A3IN | 3.04029 | 3.858666 | 2.497418 | 4.696797 | 3.349984 | 3.237667 | 4.238624 | 2.699995 | high |
| TCGA-2F-A9KT | 2.596076 | 3.619267 | 2.515388 | 4.96672 | 3.337727 | 3.305911 | 3.033562 | 6.286331 | high |
| TCGA-UY-A78N | 2.635861 | 4.347034 | 2.497418 | 2.579329 | 8.872961 | 3.217671 | 2.83786 | 3.686584 | low |
| TCGA-DK-A1A5 | 2.690217 | 4.476153 | 2.731358 | 2.439421 | 3.682287 | 3.176865 | 2.889784 | 2.906019 | low |
| TCGA-E7-A5KE | 2.63117 | 2.915057 | 2.497418 | 2.382946 | 3.560304 | 3.22731 | 2.83786 | 2.63918 | low |
| TCGA-FD-A62S | 2.603189 | 4.331222 | 2.531149 | 4.57112 | 3.50037 | 3.239005 | 8.529372 | 5.067333 | high |
| TCGA-G2-AA3B | 2.597935 | 3.146045 | 2.497418 | 5.207158 | 3.29148 | 3.176865 | 2.83786 | 2.632017 | low |
| TCGA-K4-A54R | 2.594526 | 4.278158 | 2.580027 | 4.046069 | 3.309679 | 3.328144 | 2.933396 | 2.644683 | low |
| TCGA-XF-A9T5 | 6.5343 | 6.36518 | 2.497418 | 2.642272 | 3.415379 | 3.258976 | 2.83786 | 3.190855 | high |
| TCGA-BT-A20J | 5.461917 | 4.385622 | 2.513522 | 2.382946 | 3.511018 | 3.481196 | 2.83786 | 2.859207 | high |
| TCGA-XF-AAMW | 2.634047 | 3.330984 | 2.518981 | 6.1986 | 3.831285 | 3.176865 | 4.029954 | 2.706788 | high |
| TCGA-ZF-AA4V | 2.768809 | 2.963371 | 2.553998 | 4.080342 | 3.368862 | 3.198057 | 4.642513 | 3.123794 | high |
| TCGA-GU-AATP | 3.642348 | 4.681415 | 4.461826 | 2.440145 | 3.298037 | 3.210703 | 3.95292 | 2.647176 | high |
| TCGA-CU-A0YN | 2.695173 | 3.770078 | 2.521635 | 10.764197 | 4.18388 | 3.307758 | 2.906983 | 8.955591 | high |
| TCGA-XF-AAMH | 11.651311 | 9.157171 | 2.716747 | 5.517001 | 5.089357 | 3.886887 | 2.875735 | 3.043096 | high |
| TCGA-DK-A2I6 | 2.619304 | 3.283747 | 2.505762 | 2.409278 | 3.309369 | 3.176865 | 2.83786 | 2.672577 | low |
| TCGA-DK-AA6Q | 2.617929 | 11.46172 | 9.980024 | 7.247704 | 3.308596 | 3.20682 | 2.83786 | 3.886415 | high |
| TCGA-XF-A8HI | 2.568406 | 5.382821 | 2.525608 | 2.590717 | 11.077576 | 3.254239 | 2.918199 | 2.821743 | low |
| TCGA-DK-A2I1 | 2.585503 | 5.354624 | 2.693598 | 3.206461 | 9.34869 | 3.315496 | 2.83786 | 2.812585 | low |
| TCGA-E7-A7PW | 2.6943 | 3.062957 | 2.531018 | 3.189877 | 7.146891 | 3.327779 | 3.065425 | 5.690475 | low |
| TCGA-XF-AAMT | 3.124856 | 4.691329 | 3.057809 | 3.084141 | 3.423599 | 3.218444 | 6.770357 | 3.867207 | high |
| TCGA-BT-A20R | 2.689633 | 5.222842 | 2.497418 | 2.382946 | 3.975999 | 3.240343 | 2.83786 | 2.615272 | low |
| TCGA-H4-A2HQ | 2.568406 | 3.062194 | 2.497418 | 2.467092 | 4.033631 | 3.176865 | 2.988913 | 3.089995 | low |
| TCGA-FD-A43S | 2.648653 | 6.121132 | 2.497418 | 2.464731 | 6.523286 | 3.225578 | 2.913098 | 3.334957 | low |
| TCGA-ZF-AA58 | 3.365506 | 4.337031 | 2.533649 | 2.458094 | 3.449985 | 3.370264 | 3.036439 | 2.707857 | high |
| TCGA-DK-AA6P | 2.568406 | 3.342753 | 2.497418 | 2.382946 | 10.207626 | 3.176865 | 2.885453 | 2.615272 | low |
| TCGA-XF-AAN0 | 2.583318 | 4.277712 | 2.573152 | 3.34847 | 4.969202 | 3.247589 | 2.920177 | 2.657101 | low |
| TCGA-BT-A0YX | 2.60904 | 10.457862 | 2.575192 | 8.650011 | 3.371987 | 3.201372 | 2.83786 | 2.829108 | high |
| TCGA-ZF-A9R5 | 2.568406 | 2.954121 | 2.497418 | 2.944799 | 5.840059 | 3.176865 | 2.991339 | 2.627345 | low |
| TCGA-GV-A3JX | 3.331172 | 5.659749 | 2.516321 | 6.42605 | 3.340308 | 3.32851 | 2.83786 | 6.629279 | high |
| TCGA-ZF-A9R3 | 2.568406 | 3.79104 | 2.497418 | 2.729342 | 8.04777 | 3.176865 | 2.902702 | 3.335974 | low |
| TCGA-G2-A2EO | 2.629805 | 4.206008 | 2.510851 | 2.719916 | 8.762312 | 3.201762 | 2.83786 | 2.627092 | low |
| TCGA-CF-A5UA | 2.629198 | 3.033183 | 2.497418 | 2.504289 | 3.832173 | 3.176865 | 2.913524 | 2.615272 | low |
| TCGA-K4-A3WU | 2.61073 | 6.457346 | 2.524946 | 4.538584 | 4.053216 | 3.193954 | 3.708782 | 2.82413 | high |
| TCGA-DK-AA71 | 2.620525 | 4.223335 | 2.497418 | 2.673304 | 3.788426 | 3.176865 | 2.934096 | 6.077874 | high |
| TCGA-XF-A9T2 | 2.798527 | 3.246878 | 2.523888 | 2.382946 | 3.526452 | 6.542568 | 2.913382 | 3.316584 | high |
| TCGA-FD-A5BY | 2.70417 | 4.468471 | 2.598567 | 3.579983 | 5.378812 | 3.224808 | 5.000981 | 3.492364 | high |
| TCGA-BT-A20T | 2.668018 | 4.40701 | 2.54112 | 4.203914 | 7.256142 | 3.217478 | 2.83786 | 6.201332 | low |
| TCGA-UY-A78O | 2.590177 | 3.914727 | 2.497418 | 2.427061 | 10.158896 | 3.20293 | 3.065681 | 2.675626 | low |
| TCGA-4Z-AA89 | 2.568406 | 3.028262 | 2.497418 | 3.286383 | 11.416344 | 3.176865 | 2.976042 | 2.877432 | low |
| TCGA-XF-A9SW | 2.609347 | 6.843246 | 2.649816 | 4.851272 | 5.228584 | 3.273681 | 2.913666 | 2.769865 | high |
| TCGA-BT-A20W | 2.568406 | 3.593645 | 2.551666 | 2.494746 | 7.179722 | 3.176865 | 3.083512 | 3.685204 | low |
| TCGA-FD-A3N5 | 2.594991 | 4.134788 | 2.497418 | 10.577479 | 3.303791 | 3.208762 | 4.994062 | 13.710479 | high |
| TCGA-DK-A3WX | 2.672601 | 3.883557 | 2.609048 | 6.410817 | 3.301616 | 3.240343 | 4.873283 | 5.950933 | high |
| TCGA-FD-A62N | 2.674961 | 4.90705 | 2.511653 | 2.629164 | 3.977618 | 3.304617 | 3.912206 | 2.627725 | high |
| TCGA-HQ-A5NE | 2.728579 | 4.157262 | 2.510985 | 7.558998 | 5.538669 | 3.251012 | 2.988238 | 3.481718 | low |
| TCGA-XF-A8HE | 2.62768 | 3.055307 | 2.548679 | 10.422887 | 3.379549 | 3.200788 | 3.01565 | 4.545141 | high |
| TCGA-DK-AA6R | 2.568406 | 3.781849 | 2.497418 | 10.735197 | 3.305032 | 3.176865 | 5.122199 | 4.111812 | high |
| TCGA-XF-A9SI | 2.590332 | 5.728805 | 2.52574 | 2.427426 | 4.564991 | 3.228848 | 2.878643 | 2.627725 | low |
| TCGA-BT-A42C | 2.598244 | 4.370001 | 2.507103 | 2.821609 | 6.806989 | 3.264833 | 2.920177 | 4.296159 | low |
| TCGA-KQ-A41O | 2.59111 | 2.956618 | 2.526797 | 2.382946 | 10.849339 | 3.176865 | 3.596484 | 2.653636 | low |
| TCGA-YF-AA3M | 2.634803 | 3.510686 | 2.497418 | 3.024078 | 3.333163 | 3.176865 | 3.411779 | 3.445913 | high |
| TCGA-ZF-AA4N | 2.732847 | 11.02166 | 9.105513 | 5.420419 | 3.308286 | 3.176865 | 3.012998 | 5.929744 | high |
| TCGA-G2-A2EC | 2.617318 | 6.274281 | 2.497418 | 3.130228 | 9.101855 | 3.176865 | 2.957147 | 2.633906 | low |
| TCGA-CF-A5U8 | 2.568406 | 3.206447 | 2.497418 | 2.382946 | 13.733566 | 3.176865 | 3.270232 | 2.615272 | low |
| TCGA-UY-A8OD | 2.821663 | 6.209218 | 2.512187 | 2.473681 | 3.93353 | 3.204098 | 4.287055 | 2.944582 | high |
| TCGA-4Z-AA7R | 2.726583 | 3.007466 | 2.497418 | 4.048058 | 3.284267 | 3.225963 | 3.540872 | 2.706194 | low |
| TCGA-FD-A3B4 | 3.006609 | 4.624388 | 2.797703 | 7.943492 | 3.591764 | 3.241107 | 2.863314 | 6.332517 | high |
| TCGA-FJ-A3Z7 | 2.603034 | 3.08787 | 2.497418 | 2.55084 | 3.342884 | 3.258786 | 2.870341 | 2.66388 | low |
| TCGA-G2-A2EJ | 2.708793 | 3.192799 | 2.497418 | 11.293537 | 3.319699 | 3.176865 | 2.83786 | 4.519647 | high |
| TCGA-SY-A9G0 | 2.828365 | 6.614924 | 2.515655 | 2.494054 | 5.30371 | 3.210315 | 4.243114 | 2.631134 | high |
| TCGA-G2-A2EK | 2.568406 | 4.941638 | 2.516853 | 2.382946 | 4.392417 | 3.176865 | 2.865807 | 3.242516 | low |
| TCGA-FD-A5BX | 2.640086 | 4.426236 | 2.497418 | 2.382946 | 4.243645 | 3.371516 | 2.882993 | 2.615272 | low |
| TCGA-XF-AAN4 | 2.568406 | 7.478792 | 2.497418 | 2.382946 | 4.694486 | 3.437257 | 2.83786 | 2.663757 | high |
| TCGA-FD-A3SO | 2.701563 | 3.896617 | 2.522961 | 3.899662 | 3.322616 | 3.292189 | 2.83786 | 2.691603 | low |
| TCGA-FD-A6TA | 2.592198 | 4.685379 | 3.226525 | 3.026579 | 3.386486 | 3.261434 | 2.882269 | 2.655493 | high |
| TCGA-FD-A3SS | 8.111591 | 3.81242 | 2.618832 | 3.65576 | 3.349079 | 3.212641 | 2.973046 | 8.561921 | high |
| TCGA-FD-A43P | 2.740502 | 3.21167 | 2.555163 | 2.88881 | 3.864555 | 3.316783 | 2.865953 | 2.729992 | low |
| TCGA-BT-A3PK | 2.659705 | 3.92525 | 2.504151 | 9.625485 | 3.334382 | 3.3674 | 3.36177 | 2.717559 | high |
| TCGA-XF-A9T6 | 5.671869 | 3.008491 | 2.497418 | 2.602852 | 3.306892 | 3.176865 | 4.018629 | 2.704766 | high |
| TCGA-FD-A5BV | 3.020997 | 3.363146 | 2.497418 | 2.55997 | 4.21982 | 3.176865 | 2.83786 | 2.7634 | low |
| TCGA-ZF-AA4U | 2.568406 | 3.255943 | 2.497418 | 2.676806 | 3.891271 | 3.204293 | 2.880384 | 4.723602 | low |
| TCGA-SY-A9G5 | 2.687295 | 4.506983 | 3.313651 | 2.526655 | 3.433635 | 3.263701 | 2.83786 | 2.656483 | high |
| TCGA-KQ-A41R | 2.716565 | 4.193959 | 2.497418 | 3.326581 | 3.509793 | 3.176865 | 2.83786 | 2.68376 | low |
| TCGA-GC-A6I1 | 3.183804 | 3.675135 | 2.525872 | 3.835434 | 3.448145 | 3.203125 | 4.223954 | 3.713882 | high |
| TCGA-GU-A764 | 2.837832 | 4.403757 | 2.665164 | 3.392444 | 3.849928 | 3.176865 | 3.090964 | 2.771561 | high |
| TCGA-G2-A2EF | 2.592974 | 2.992443 | 2.497418 | 3.739817 | 3.741431 | 3.206237 | 3.311006 | 4.357812 | low |
| TCGA-FT-A61P | 2.611344 | 5.55289 | 2.525343 | 2.426817 | 3.538263 | 3.327048 | 3.540499 | 2.687024 | high |
| TCGA-K4-AAQO | 2.591887 | 6.523148 | 2.512721 | 2.520903 | 8.915379 | 3.176865 | 2.881544 | 6.233826 | high |
| TCGA-DK-AA6L | 2.594061 | 3.210512 | 2.594427 | 2.532941 | 4.138634 | 3.176865 | 3.103883 | 2.68642 | low |
| TCGA-K4-A6MB | 2.72487 | 3.865375 | 2.508041 | 6.074762 | 4.050033 | 3.234987 | 4.082798 | 8.500511 | high |
| TCGA-BT-A20P | 2.603497 | 4.137609 | 2.520176 | 2.45381 | 7.880437 | 3.176865 | 2.965392 | 2.615272 | low |
| TCGA-FD-A3SJ | 3.116299 | 5.005225 | 2.497418 | 2.464376 | 6.259246 | 3.176865 | 3.662864 | 2.788397 | low |
| TCGA-G2-A3VY | 2.673192 | 5.147432 | 2.497418 | 2.414332 | 6.088289 | 3.176865 | 3.028316 | 2.624052 | low |
| TCGA-GC-A3WC | 2.568406 | 11.546067 | 7.964043 | 4.373659 | 3.553036 | 3.201567 | 2.985807 | 4.09485 | high |
| TCGA-CU-A3QU | 2.599791 | 2.929594 | 2.497418 | 2.53596 | 3.284267 | 3.176865 | 2.83786 | 2.615272 | low |
| TCGA-FD-A3SP | 2.637824 | 5.109454 | 3.40221 | 2.382946 | 3.301461 | 3.359324 | 2.995776 | 3.016982 | high |
| TCGA-GC-A3RB | 2.597625 | 3.438608 | 2.497418 | 2.792874 | 6.10504 | 3.211866 | 2.918623 | 3.377484 | low |
| TCGA-GU-AATQ | 2.568406 | 11.112718 | 2.843605 | 3.643984 | 3.342581 | 3.176865 | 3.236448 | 2.746114 | high |
| TCGA-GC-A6I3 | 2.594681 | 3.626452 | 2.497418 | 2.628853 | 5.194182 | 3.20818 | 3.408117 | 2.615272 | low |
| TCGA-GC-A3RD | 2.666092 | 3.088191 | 2.523358 | 4.337158 | 3.595378 | 3.176865 | 2.911678 | 5.064204 | high |
| TCGA-GD-A3OQ | 2.693134 | 3.505605 | 2.515921 | 5.252852 | 4.193075 | 3.21109 | 7.410855 | 3.174479 | high |
| TCGA-CF-A47T | 2.590332 | 3.385052 | 2.497418 | 4.339218 | 8.34239 | 3.203125 | 3.551366 | 3.586227 | low |
| TCGA-ZF-AA52 | 2.740502 | 4.747585 | 3.967544 | 2.580084 | 3.358552 | 3.268038 | 2.977402 | 2.754385 | high |
| TCGA-XF-A8HG | 2.59111 | 3.026243 | 2.497418 | 2.382946 | 9.534661 | 3.176865 | 2.83786 | 2.615272 | low |
| TCGA-FD-A3B7 | 5.573143 | 3.724319 | 3.332328 | 3.603821 | 3.624885 | 3.195909 | 4.18179 | 2.837826 | high |
| TCGA-DK-A3IL | 2.685978 | 5.239247 | 2.497418 | 4.549244 | 3.810508 | 3.176865 | 2.83786 | 5.30817 | high |
| TCGA-E7-A6ME | 2.592663 | 3.469671 | 2.497418 | 2.382946 | 5.118833 | 3.206043 | 3.09109 | 2.615272 | low |
| TCGA-GD-A2C5 | 2.568406 | 4.643231 | 2.519645 | 2.382946 | 9.329097 | 3.176865 | 3.020412 | 2.788397 | low |
| TCGA-DK-AA6X | 2.805735 | 3.453286 | 2.520972 | 2.524966 | 3.531563 | 3.304062 | 2.904987 | 2.926197 | low |
| TCGA-UY-A9PD | 2.693571 | 4.376864 | 2.497418 | 3.157679 | 5.709092 | 3.176865 | 2.955355 | 2.834819 | low |
| TCGA-DK-A3IM | 3.495299 | 2.973138 | 2.613144 | 6.489902 | 3.443749 | 3.193954 | 4.910785 | 8.448569 | high |
| TCGA-BT-A42F | 2.656427 | 3.749625 | 2.543601 | 4.542413 | 3.293044 | 3.440194 | 2.871509 | 3.038705 | low |
| TCGA-FD-A3SL | 2.736821 | 5.144672 | 2.510183 | 2.382946 | 3.674582 | 3.200398 | 3.668692 | 2.821199 | high |
| TCGA-BT-A3PH | 2.611804 | 5.327233 | 2.516321 | 4.778513 | 3.540935 | 3.194345 | 2.83786 | 2.631765 | high |
| TCGA-FD-A3SQ | 2.606271 | 5.797558 | 2.581804 | 2.382946 | 4.163801 | 3.199618 | 2.83786 | 3.218222 | high |
| TCGA-ZF-A9RF | 2.780295 | 2.924483 | 2.523888 | 2.382946 | 3.400561 | 4.4306 | 2.83786 | 2.694486 | high |
| TCGA-DK-A1A3 | 2.677318 | 4.377201 | 2.497418 | 2.743255 | 3.431203 | 3.203709 | 2.83786 | 2.804793 | low |
| TCGA-BT-A2LA | 5.028726 | 3.840283 | 2.549719 | 2.726272 | 5.881637 | 3.257272 | 2.863461 | 2.615272 | high |
| TCGA-PQ-A6FN | 2.615635 | 3.926815 | 2.543209 | 7.173865 | 3.440053 | 3.261056 | 2.83786 | 6.108323 | high |
| TCGA-GD-A3OP | 2.582537 | 5.30987 | 2.506567 | 2.382946 | 10.858265 | 3.210509 | 2.83786 | 2.631134 | low |
| TCGA-FD-A5BT | 9.979667 | 4.528162 | 2.497418 | 2.432529 | 3.399977 | 3.346151 | 3.240215 | 2.7846 | high |
| TCGA-BL-A3JM | 2.647603 | 9.195114 | 2.506165 | 2.382946 | 3.52281 | 3.317518 | 2.83786 | 2.645556 | high |
| TCGA-E7-A85H | 2.585815 | 3.42902 | 2.497418 | 2.382946 | 3.92707 | 3.238814 | 2.83786 | 2.625193 | low |
| TCGA-CU-A72E | 2.583943 | 9.662511 | 2.72954 | 6.097166 | 3.321848 | 3.176865 | 4.325228 | 5.8333 | high |
| TCGA-FD-A3SN | 4.836582 | 4.017562 | 2.510049 | 2.534843 | 3.340762 | 3.246065 | 3.19661 | 2.669644 | high |
| TCGA-GU-A762 | 2.627073 | 3.89457 | 2.510316 | 2.382946 | 3.559909 | 3.176865 | 3.013927 | 2.64867 | low |
| TCGA-GV-A3QH | 2.638729 | 3.040972 | 2.497418 | 3.65037 | 3.748231 | 3.176865 | 2.864634 | 2.623418 | low |
| TCGA-FD-A6TF | 2.676287 | 5.769005 | 2.497418 | 4.728901 | 3.504064 | 3.285105 | 3.003009 | 2.914331 | high |
| TCGA-XF-A9SJ | 4.321052 | 4.26426 | 2.497418 | 2.469214 | 3.366776 | 3.25329 | 3.132826 | 2.787504 | high |
| TCGA-ZF-A9RL | 2.592663 | 2.980966 | 2.497418 | 2.382946 | 11.431652 | 3.176865 | 2.83786 | 3.636387 | low |
| TCGA-E7-A678 | 2.568406 | 3.272591 | 2.515522 | 2.382946 | 11.09549 | 3.176865 | 2.83786 | 2.615272 | low |
| TCGA-GD-A76B | 2.651947 | 3.441493 | 2.508443 | 2.824545 | 5.183844 | 3.217478 | 2.83786 | 2.62494 | low |
| TCGA-GV-A6ZA | 2.568406 | 3.973078 | 2.524814 | 2.508292 | 4.778082 | 3.227502 | 3.095366 | 2.662773 | low |
| TCGA-FD-A5C1 | 2.854099 | 4.286921 | 2.721444 | 7.463787 | 3.537728 | 3.201762 | 2.876608 | 5.997054 | high |
| TCGA-BT-A20X | 2.583005 | 3.296606 | 2.675616 | 7.437525 | 3.624505 | 3.312736 | 4.475241 | 4.193582 | high |
| TCGA-K4-A3WV | 4.812974 | 6.550977 | 2.497418 | 8.961122 | 3.29445 | 3.201957 | 2.876899 | 5.624463 | high |
| TCGA-FD-A5C0 | 2.588153 | 5.145393 | 2.510183 | 3.373958 | 3.598598 | 3.269544 | 2.83786 | 4.253905 | high |
| TCGA-DK-A3IU | 3.372815 | 5.596156 | 2.517252 | 4.242283 | 3.475379 | 3.319354 | 2.83786 | 7.721289 | high |
| TCGA-UY-A78K | 2.586595 | 3.637082 | 2.520972 | 3.441925 | 3.429628 | 3.176865 | 2.83786 | 2.676113 | low |
| TCGA-DK-A1AE | 2.580034 | 11.24327 | 9.063965 | 5.772256 | 3.317854 | 3.176865 | 3.133438 | 2.697962 | high |
| TCGA-LT-A8JT | 2.568406 | 3.276535 | 2.508979 | 2.454286 | 12.953542 | 3.176865 | 2.935215 | 2.674529 | low |
| TCGA-BT-A20O | 2.63888 | 9.04546 | 8.367909 | 2.478016 | 3.375402 | 3.31568 | 3.5154 | 2.68158 | high |
| TCGA-K4-A4AB | 2.670829 | 5.091521 | 2.652707 | 2.849792 | 3.691169 | 3.176865 | 2.877044 | 2.673798 | low |
| TCGA-FD-A43U | 2.589243 | 4.912625 | 2.524285 | 2.720012 | 7.917643 | 3.75762 | 2.987023 | 3.093249 | low |
| TCGA-FD-A6TE | 2.611651 | 3.096806 | 2.497418 | 2.603064 | 3.802617 | 3.211672 | 2.83786 | 2.671967 | low |
| TCGA-XF-A9SV | 2.568406 | 4.266594 | 2.574428 | 2.540087 | 8.003714 | 3.176865 | 2.83786 | 2.615272 | low |
| TCGA-GD-A6C6 | 2.568406 | 3.059903 | 2.497418 | 2.382946 | 3.598856 | 3.176865 | 2.83786 | 2.62861 | low |
| TCGA-KQ-A41N | 7.885593 | 3.661957 | 2.497418 | 2.427913 | 6.516085 | 3.562999 | 5.839329 | 2.640183 | high |
| TCGA-4Z-AA7W | 2.968038 | 3.276168 | 2.576976 | 3.508174 | 3.296478 | 3.20682 | 2.83786 | 2.724846 | low |
| TCGA-CF-A9FH | 2.593905 | 3.721262 | 2.497418 | 2.382946 | 9.2606 | 3.207403 | 2.885308 | 2.615272 | low |
| TCGA-KQ-A41Q | 2.694591 | 3.768914 | 2.707419 | 2.551172 | 5.357358 | 3.176865 | 2.993895 | 2.710823 | low |
| TCGA-E7-A3X6 | 2.587374 | 3.384633 | 2.638189 | 2.598501 | 3.311997 | 3.176865 | 3.398203 | 4.37759 | high |
| TCGA-XF-A9SL | 2.632533 | 6.596042 | 2.552832 | 2.382946 | 4.116392 | 3.25367 | 2.956044 | 2.710468 | high |
| TCGA-4Z-AA81 | 2.664016 | 2.948633 | 2.513255 | 4.158862 | 3.284267 | 3.176865 | 3.693548 | 2.656359 | low |
| TCGA-DK-A1AB | 2.805735 | 4.691561 | 2.840154 | 3.580617 | 4.199935 | 3.207209 | 2.83786 | 7.104349 | high |
| TCGA-XF-A9T0 | 2.568406 | 4.058604 | 3.060658 | 3.035931 | 7.889534 | 3.206043 | 2.83786 | 3.329276 | low |
| TCGA-GC-A3RC | 2.568406 | 2.993135 | 2.5347 | 8.803492 | 3.376143 | 3.176865 | 3.502834 | 9.833192 | high |
| TCGA-XF-AAN8 | 2.6001 | 6.373363 | 2.497418 | 2.382946 | 4.195189 | 3.28865 | 2.953422 | 3.10592 | high |
| TCGA-XF-A8HF | 2.610269 | 4.269332 | 2.497418 | 3.215096 | 4.354695 | 3.202151 | 2.877189 | 4.706586 | high |
| TCGA-E5-A2PC | 2.62083 | 11.645459 | 2.531544 | 2.382946 | 3.297258 | 3.176865 | 2.83786 | 2.645057 | high |
| TCGA-ZF-A9R2 | 2.592974 | 2.970909 | 2.544775 | 2.614871 | 5.719573 | 3.176865 | 2.971273 | 2.77348 | low |
| TCGA-E5-A4U1 | 2.568406 | 3.101461 | 2.497418 | 2.382946 | 12.869574 | 3.205654 | 2.83786 | 5.730109 | low |
| TCGA-BT-A0S7 | 2.660301 | 5.101993 | 2.497418 | 8.144131 | 3.565307 | 3.251012 | 3.057716 | 6.841331 | high |
| TCGA-ZF-A9RM | 3.24905 | 4.070419 | 2.497418 | 2.422914 | 7.29721 | 3.176865 | 2.83786 | 2.637425 | low |
| TCGA-UY-A9PB | 2.71556 | 4.447323 | 2.683873 | 2.532157 | 3.495156 | 3.296093 | 3.181036 | 2.615272 | high |
| TCGA-XF-A9SY | 2.982779 | 4.307551 | 2.673248 | 2.6717 | 3.347269 | 3.269168 | 2.933536 | 2.630125 | high |
| TCGA-E7-A7DU | 2.568406 | 3.341972 | 2.497418 | 2.454167 | 8.513731 | 3.219024 | 2.903416 | 2.615272 | low |
| TCGA-XF-AAMJ | 2.887428 | 4.824068 | 2.535094 | 2.382946 | 3.572388 | 3.345788 | 2.83786 | 2.648172 | high |

# Appendix 9

## **GO and KEGG enrichment analysis**

**Table 7a. GO enrichment analysis.**

| ONTOLOGY | ID | Description | BgRatio | pvalue | qvalue |
| --- | --- | --- | --- | --- | --- |
| BP | GO:0072521 | purine-containing compound metabolic process | 460/18862 | 7.96E-56 | 7.44E-53 |
| BP | GO:0006163 | purine nucleotide metabolic process | 441/18862 | 1.36E-53 | 6.36E-51 |
| BP | GO:0019693 | ribose phosphate metabolic process | 435/18862 | 1.01E-50 | 3.16E-48 |
| BP | GO:0009150 | purine ribonucleotide metabolic process | 408/18862 | 1.57E-50 | 3.67E-48 |
| BP | GO:0009259 | ribonucleotide metabolic process | 425/18862 | 1.19E-49 | 2.22E-47 |
| BP | GO:1901293 | nucleoside phosphate biosynthetic process | 267/18862 | 5.73E-46 | 8.23E-44 |
| BP | GO:0034404 | nucleobase-containing small molecule biosynthetic process | 115/18862 | 6.16E-46 | 8.23E-44 |
| BP | GO:0072522 | purine-containing compound biosynthetic process | 208/18862 | 2.56E-45 | 3.00E-43 |
| BP | GO:0009165 | nucleotide biosynthetic process | 264/18862 | 1.63E-44 | 1.56E-42 |
| BP | GO:0006164 | purine nucleotide biosynthetic process | 197/18862 | 1.67E-44 | 1.56E-42 |
| BP | GO:0009126 | purine nucleoside monophosphate metabolic process | 43/18862 | 1.52E-43 | 1.29E-41 |
| BP | GO:0009123 | nucleoside monophosphate metabolic process | 75/18862 | 2.93E-42 | 2.28E-40 |
| BP | GO:0009152 | purine ribonucleotide biosynthetic process | 175/18862 | 3.49E-41 | 2.51E-39 |
| BP | GO:0009260 | ribonucleotide biosynthetic process | 188/18862 | 4.46E-40 | 2.98E-38 |
| BP | GO:0046390 | ribose phosphate biosynthetic process | 195/18862 | 1.62E-39 | 1.01E-37 |
| BP | GO:0009167 | purine ribonucleoside monophosphate metabolic process | 40/18862 | 2.36E-37 | 1.38E-35 |
| BP | GO:0009116 | nucleoside metabolic process | 104/18862 | 4.99E-34 | 2.75E-32 |
| BP | GO:0009161 | ribonucleoside monophosphate metabolic process | 56/18862 | 2.25E-33 | 1.17E-31 |
| BP | GO:1901292 | nucleoside phosphate catabolic process | 83/18862 | 6.19E-33 | 3.04E-31 |
| BP | GO:0009127 | purine nucleoside monophosphate biosynthetic process | 23/18862 | 1.59E-31 | 7.42E-30 |
| BP | GO:1901657 | glycosyl compound metabolic process | 129/18862 | 1.88E-31 | 8.35E-30 |
| BP | GO:0009187 | cyclic nucleotide metabolic process | 40/18862 | 2.68E-28 | 1.14E-26 |
| BP | GO:0009124 | nucleoside monophosphate biosynthetic process | 43/18862 | 1.26E-27 | 5.11E-26 |
| BP | GO:0009168 | purine ribonucleoside monophosphate biosynthetic process | 21/18862 | 2.87E-27 | 1.12E-25 |
| BP | GO:0046128 | purine ribonucleoside metabolic process | 58/18862 | 6.28E-27 | 2.35E-25 |
| BP | GO:0042278 | purine nucleoside metabolic process | 61/18862 | 1.80E-26 | 6.49E-25 |
| BP | GO:0046434 | organophosphate catabolic process | 154/18862 | 3.46E-26 | 1.20E-24 |
| BP | GO:0072523 | purine-containing compound catabolic process | 51/18862 | 4.24E-26 | 1.42E-24 |
| BP | GO:0009119 | ribonucleoside metabolic process | 72/18862 | 5.41E-25 | 1.74E-23 |
| BP | GO:0006195 | purine nucleotide catabolic process | 46/18862 | 5.75E-25 | 1.79E-23 |
| BP | GO:0009156 | ribonucleoside monophosphate biosynthetic process | 34/18862 | 3.23E-23 | 9.73E-22 |
| BP | GO:0009166 | nucleotide catabolic process | 73/18862 | 4.58E-23 | 1.34E-21 |
| BP | GO:0009154 | purine ribonucleotide catabolic process | 35/18862 | 5.35E-23 | 1.52E-21 |
| BP | GO:0034656 | nucleobase-containing small molecule catabolic process | 50/18862 | 2.50E-22 | 6.87E-21 |
| BP | GO:0009261 | ribonucleotide catabolic process | 40/18862 | 5.23E-22 | 1.40E-20 |
| BP | GO:0009141 | nucleoside triphosphate metabolic process | 109/18862 | 1.83E-21 | 4.75E-20 |
| BP | GO:0032201 | telomere maintenance via semi-conservative replication | 27/18862 | 1.51E-20 | 3.82E-19 |
| BP | GO:0042451 | purine nucleoside biosynthetic process | 22/18862 | 1.18E-19 | 2.75E-18 |
| BP | GO:0042455 | ribonucleoside biosynthetic process | 22/18862 | 1.18E-19 | 2.75E-18 |
| BP | GO:0046129 | purine ribonucleoside biosynthetic process | 22/18862 | 1.18E-19 | 2.75E-18 |
| BP | GO:0032481 | positive regulation of type I interferon production | 77/18862 | 3.44E-19 | 7.85E-18 |
| BP | GO:0009163 | nucleoside biosynthetic process | 38/18862 | 2.23E-18 | 4.96E-17 |
| BP | GO:0009262 | deoxyribonucleotide metabolic process | 40/18862 | 4.56E-18 | 9.68E-17 |
| BP | GO:1901068 | guanosine-containing compound metabolic process | 40/18862 | 4.56E-18 | 9.68E-17 |
| BP | GO:1901136 | carbohydrate derivative catabolic process | 198/18862 | 5.26E-18 | 1.09E-16 |
| BP | GO:1901659 | glycosyl compound biosynthetic process | 42/18862 | 8.93E-18 | 1.82E-16 |
| BP | GO:0009151 | purine deoxyribonucleotide metabolic process | 14/18862 | 1.19E-17 | 2.31E-16 |
| BP | GO:0046040 | IMP metabolic process | 14/18862 | 1.19E-17 | 2.31E-16 |
| BP | GO:0046058 | cAMP metabolic process | 22/18862 | 2.01E-17 | 3.83E-16 |
| BP | GO:0032479 | regulation of type I interferon production | 128/18862 | 3.66E-17 | 6.85E-16 |
| BP | GO:0009132 | nucleoside diphosphate metabolic process | 156/18862 | 3.82E-17 | 6.99E-16 |
| BP | GO:0032606 | type I interferon production | 129/18862 | 4.16E-17 | 7.48E-16 |
| BP | GO:0006188 | IMP biosynthetic process | 10/18862 | 4.96E-17 | 8.75E-16 |
| BP | GO:0046033 | AMP metabolic process | 16/18862 | 6.72E-17 | 1.16E-15 |
| BP | GO:0072527 | pyrimidine-containing compound metabolic process | 85/18862 | 6.89E-17 | 1.17E-15 |
| BP | GO:0009144 | purine nucleoside triphosphate metabolic process | 86/18862 | 8.19E-17 | 1.37E-15 |
| BP | GO:0015949 | nucleobase-containing small molecule interconversion | 27/18862 | 2.55E-16 | 4.19E-15 |
| BP | GO:0006283 | transcription-coupled nucleotide-excision repair | 73/18862 | 3.17E-16 | 5.10E-15 |
| BP | GO:0006144 | purine nucleobase metabolic process | 19/18862 | 5.35E-16 | 8.47E-15 |
| BP | GO:0033260 | nuclear DNA replication | 59/18862 | 8.33E-16 | 1.30E-14 |
| BP | GO:0009142 | nucleoside triphosphate biosynthetic process | 84/18862 | 2.18E-15 | 3.34E-14 |
| BP | GO:0006289 | nucleotide-excision repair | 108/18862 | 2.27E-15 | 3.42E-14 |
| BP | GO:0044786 | cell cycle DNA replication | 64/18862 | 2.38E-15 | 3.54E-14 |
| BP | GO:0009190 | cyclic nucleotide biosynthetic process | 22/18862 | 2.84E-15 | 4.08E-14 |
| BP | GO:0052652 | cyclic purine nucleotide metabolic process | 22/18862 | 2.84E-15 | 4.08E-14 |
| BP | GO:0009214 | cyclic nucleotide catabolic process | 14/18862 | 3.25E-15 | 4.60E-14 |
| BP | GO:0009112 | nucleobase metabolic process | 34/18862 | 3.84E-15 | 5.35E-14 |
| BP | GO:0009394 | 2'-deoxyribonucleotide metabolic process | 36/18862 | 7.36E-15 | 1.01E-13 |
| BP | GO:0019692 | deoxyribose phosphate metabolic process | 38/18862 | 1.36E-14 | 1.84E-13 |
| BP | GO:0050434 | positive regulation of viral transcription | 26/18862 | 1.75E-14 | 2.33E-13 |
| BP | GO:0009113 | purine nucleobase biosynthetic process | 10/18862 | 2.39E-14 | 3.14E-13 |
| BP | GO:0006362 | transcription elongation from RNA polymerase I promoter | 30/18862 | 7.85E-14 | 1.02E-12 |
| BP | GO:0006363 | termination of RNA polymerase I transcription | 31/18862 | 1.10E-13 | 1.41E-12 |
| BP | GO:0006370 | 7-methylguanosine mRNA capping | 33/18862 | 2.09E-13 | 2.63E-12 |
| BP | GO:0006354 | DNA-templated transcription, elongation | 119/18862 | 2.29E-13 | 2.86E-12 |
| BP | GO:0006220 | pyrimidine nucleotide metabolic process | 50/18862 | 2.78E-13 | 3.34E-12 |
| BP | GO:0009164 | nucleoside catabolic process | 34/18862 | 2.82E-13 | 3.34E-12 |
| BP | GO:0009452 | 7-methylguanosine RNA capping | 34/18862 | 2.82E-13 | 3.34E-12 |
| BP | GO:0036260 | RNA capping | 34/18862 | 2.82E-13 | 3.34E-12 |
| BP | GO:1901070 | guanosine-containing compound biosynthetic process | 13/18862 | 3.36E-13 | 3.93E-12 |
| BP | GO:0006361 | transcription initiation from RNA polymerase I promoter | 38/18862 | 8.60E-13 | 9.93E-12 |
| BP | GO:0009263 | deoxyribonucleotide biosynthetic process | 15/18862 | 1.25E-12 | 1.41E-11 |
| BP | GO:0043101 | purine-containing compound salvage | 15/18862 | 1.25E-12 | 1.41E-11 |
| BP | GO:0009205 | purine ribonucleoside triphosphate metabolic process | 80/18862 | 1.32E-12 | 1.47E-11 |
| BP | GO:0046782 | regulation of viral transcription | 42/18862 | 2.31E-12 | 2.54E-11 |
| BP | GO:0009199 | ribonucleoside triphosphate metabolic process | 87/18862 | 3.41E-12 | 3.70E-11 |
| BP | GO:1901658 | glycosyl compound catabolic process | 44/18862 | 3.63E-12 | 3.90E-11 |
| BP | GO:0046112 | nucleobase biosynthetic process | 18/18862 | 6.09E-12 | 6.47E-11 |
| BP | GO:0006383 | transcription by RNA polymerase III | 47/18862 | 6.88E-12 | 7.23E-11 |
| BP | GO:0009145 | purine nucleoside triphosphate biosynthetic process | 68/18862 | 7.20E-12 | 7.48E-11 |
| BP | GO:0009133 | nucleoside diphosphate biosynthetic process | 10/18862 | 7.46E-12 | 7.64E-11 |
| BP | GO:0044282 | small molecule catabolic process | 431/18862 | 7.52E-12 | 7.64E-11 |
| BP | GO:0006353 | DNA-templated transcription, termination | 75/18862 | 1.98E-11 | 1.99E-10 |
| BP | GO:0098781 | ncRNA transcription | 109/18862 | 4.19E-11 | 4.17E-10 |
| BP | GO:0045815 | positive regulation of gene expression, epigenetic | 58/18862 | 5.10E-11 | 5.01E-10 |
| BP | GO:0009125 | nucleoside monophosphate catabolic process | 13/18862 | 6.01E-11 | 5.85E-10 |
| BP | GO:0046039 | GTP metabolic process | 24/18862 | 6.43E-11 | 6.20E-10 |
| BP | GO:0006270 | DNA replication initiation | 40/18862 | 7.32E-11 | 6.99E-10 |
| BP | GO:0006260 | DNA replication | 280/18862 | 9.78E-11 | 9.24E-10 |
| BP | GO:0006261 | DNA-dependent DNA replication | 157/18862 | 1.40E-10 | 1.31E-09 |
| BP | GO:0000723 | telomere maintenance | 161/18862 | 1.88E-10 | 1.74E-09 |
| BP | GO:0009206 | purine ribonucleoside triphosphate biosynthetic process | 67/18862 | 1.96E-10 | 1.79E-09 |
| BP | GO:0006360 | transcription by RNA polymerase I | 68/18862 | 2.25E-10 | 2.04E-09 |
| BP | GO:0006352 | DNA-templated transcription, initiation | 249/18862 | 2.28E-10 | 2.05E-09 |
| BP | GO:0009218 | pyrimidine ribonucleotide metabolic process | 29/18862 | 2.83E-10 | 2.52E-09 |
| BP | GO:0035019 | somatic stem cell population maintenance | 71/18862 | 3.35E-10 | 2.95E-09 |
| BP | GO:0046068 | cGMP metabolic process | 17/18862 | 4.25E-10 | 3.72E-09 |
| BP | GO:0009201 | ribonucleoside triphosphate biosynthetic process | 73/18862 | 4.32E-10 | 3.74E-09 |
| BP | GO:0032200 | telomere organization | 174/18862 | 4.63E-10 | 3.97E-09 |
| BP | GO:0042795 | snRNA transcription by RNA polymerase II | 74/18862 | 4.89E-10 | 4.16E-09 |
| BP | GO:0009301 | snRNA transcription | 75/18862 | 5.53E-10 | 4.66E-09 |
| BP | GO:0001819 | positive regulation of cytokine production | 437/18862 | 7.25E-10 | 6.05E-09 |
| BP | GO:0043094 | cellular metabolic compound salvage | 34/18862 | 9.52E-10 | 7.88E-09 |
| BP | GO:0006171 | cAMP biosynthetic process | 10/18862 | 1.59E-09 | 1.30E-08 |
| BP | GO:0019935 | cyclic-nucleotide-mediated signaling | 89/18862 | 2.62E-09 | 2.13E-08 |
| BP | GO:0006368 | transcription elongation from RNA polymerase II promoter | 90/18862 | 2.89E-09 | 2.31E-08 |
| BP | GO:0006183 | GTP biosynthetic process | 11/18862 | 2.90E-09 | 2.31E-08 |
| BP | GO:0048524 | positive regulation of viral process | 91/18862 | 3.19E-09 | 2.53E-08 |
| BP | GO:0009134 | nucleoside diphosphate catabolic process | 12/18862 | 4.94E-09 | 3.78E-08 |
| BP | GO:0009265 | 2'-deoxyribonucleotide biosynthetic process | 12/18862 | 4.94E-09 | 3.78E-08 |
| BP | GO:0046051 | UTP metabolic process | 12/18862 | 4.94E-09 | 3.78E-08 |
| BP | GO:0046385 | deoxyribose phosphate biosynthetic process | 12/18862 | 4.94E-09 | 3.78E-08 |
| BP | GO:0046940 | nucleoside monophosphate phosphorylation | 13/18862 | 7.99E-09 | 6.07E-08 |
| BP | GO:0009143 | nucleoside triphosphate catabolic process | 14/18862 | 1.24E-08 | 9.25E-08 |
| BP | GO:0043173 | nucleotide salvage | 14/18862 | 1.24E-08 | 9.25E-08 |
| BP | GO:0046037 | GMP metabolic process | 15/18862 | 1.85E-08 | 1.37E-07 |
| BP | GO:0008543 | fibroblast growth factor receptor signaling pathway | 113/18862 | 2.18E-08 | 1.61E-07 |
| BP | GO:0000731 | DNA synthesis involved in DNA repair | 53/18862 | 2.49E-08 | 1.82E-07 |
| BP | GO:0009200 | deoxyribonucleoside triphosphate metabolic process | 16/18862 | 2.67E-08 | 1.94E-07 |
| BP | GO:0009162 | deoxyribonucleoside monophosphate metabolic process | 17/18862 | 3.77E-08 | 2.71E-07 |
| BP | GO:0060964 | regulation of gene silencing by miRNA | 121/18862 | 3.98E-08 | 2.84E-07 |
| BP | GO:0046148 | pigment biosynthetic process | 57/18862 | 4.19E-08 | 2.97E-07 |
| BP | GO:0060147 | regulation of posttranscriptional gene silencing | 124/18862 | 4.92E-08 | 3.46E-07 |
| BP | GO:0009208 | pyrimidine ribonucleoside triphosphate metabolic process | 18/18862 | 5.20E-08 | 3.62E-07 |
| BP | GO:0060966 | regulation of gene silencing by RNA | 125/18862 | 5.28E-08 | 3.65E-07 |
| BP | GO:0006213 | pyrimidine nucleoside metabolic process | 36/18862 | 6.11E-08 | 4.20E-07 |
| BP | GO:0006271 | DNA strand elongation involved in DNA replication | 19/18862 | 7.02E-08 | 4.79E-07 |
| BP | GO:0019933 | cAMP-mediated signaling | 62/18862 | 7.61E-08 | 5.15E-07 |
| BP | GO:0006165 | nucleoside diphosphate phosphorylation | 132/18862 | 8.46E-08 | 5.69E-07 |
| BP | GO:0046939 | nucleotide phosphorylation | 133/18862 | 9.03E-08 | 6.03E-07 |
| BP | GO:0072528 | pyrimidine-containing compound biosynthetic process | 40/18862 | 1.18E-07 | 7.84E-07 |
| BP | GO:0009185 | ribonucleoside diphosphate metabolic process | 138/18862 | 1.24E-07 | 8.17E-07 |
| BP | GO:0009220 | pyrimidine ribonucleotide biosynthetic process | 22/18862 | 1.57E-07 | 1.02E-06 |
| BP | GO:0044344 | cellular response to fibroblast growth factor stimulus | 142/18862 | 1.59E-07 | 1.03E-06 |
| BP | GO:0060968 | regulation of gene silencing | 144/18862 | 1.79E-07 | 1.15E-06 |
| BP | GO:0006297 | nucleotide-excision repair, DNA gap filling | 23/18862 | 1.99E-07 | 1.27E-06 |
| BP | GO:0046135 | pyrimidine nucleoside catabolic process | 23/18862 | 1.99E-07 | 1.27E-06 |
| BP | GO:0019827 | stem cell population maintenance | 146/18862 | 2.01E-07 | 1.27E-06 |
| BP | GO:0042440 | pigment metabolic process | 72/18862 | 2.17E-07 | 1.36E-06 |
| BP | GO:0071774 | response to fibroblast growth factor | 148/18862 | 2.26E-07 | 1.40E-06 |
| BP | GO:0098727 | maintenance of cell number | 148/18862 | 2.26E-07 | 1.40E-06 |
| BP | GO:0006152 | purine nucleoside catabolic process | 10/18862 | 2.32E-07 | 1.41E-06 |
| BP | GO:0006228 | UTP biosynthetic process | 10/18862 | 2.32E-07 | 1.41E-06 |
| BP | GO:0046130 | purine ribonucleoside catabolic process | 10/18862 | 2.32E-07 | 1.41E-06 |
| BP | GO:0009147 | pyrimidine nucleoside triphosphate metabolic process | 25/18862 | 3.12E-07 | 1.88E-06 |
| BP | GO:0009264 | deoxyribonucleotide catabolic process | 26/18862 | 3.84E-07 | 2.29E-06 |
| BP | GO:0022616 | DNA strand elongation | 26/18862 | 3.84E-07 | 2.29E-06 |
| BP | GO:0046386 | deoxyribose phosphate catabolic process | 27/18862 | 4.69E-07 | 2.78E-06 |
| BP | GO:0006221 | pyrimidine nucleotide biosynthetic process | 30/18862 | 8.17E-07 | 4.80E-06 |
| BP | GO:0006241 | CTP biosynthetic process | 14/18862 | 1.09E-06 | 6.31E-06 |
| BP | GO:0043951 | negative regulation of cAMP-mediated signaling | 14/18862 | 1.09E-06 | 6.31E-06 |
| BP | GO:0019083 | viral transcription | 180/18862 | 1.18E-06 | 6.79E-06 |
| BP | GO:0009135 | purine nucleoside diphosphate metabolic process | 135/18862 | 1.33E-06 | 7.58E-06 |
| BP | GO:0009179 | purine ribonucleoside diphosphate metabolic process | 135/18862 | 1.33E-06 | 7.58E-06 |
| BP | GO:0032728 | positive regulation of interferon-beta production | 33/18862 | 1.34E-06 | 7.60E-06 |
| BP | GO:0009209 | pyrimidine ribonucleoside triphosphate biosynthetic process | 15/18862 | 1.47E-06 | 8.31E-06 |
| BP | GO:0050792 | regulation of viral process | 186/18862 | 1.55E-06 | 8.65E-06 |
| BP | GO:0006367 | transcription initiation from RNA polymerase II promoter | 187/18862 | 1.62E-06 | 8.99E-06 |
| BP | GO:0090305 | nucleic acid phosphodiester bond hydrolysis | 305/18862 | 1.86E-06 | 1.03E-05 |
| BP | GO:0043174 | nucleoside salvage | 16/18862 | 1.96E-06 | 1.07E-05 |
| BP | GO:0046036 | CTP metabolic process | 16/18862 | 1.96E-06 | 1.07E-05 |
| BP | GO:0071897 | DNA biosynthetic process | 194/18862 | 2.19E-06 | 1.19E-05 |
| BP | GO:0043903 | regulation of biological process involved in symbiotic interaction | 197/18862 | 2.49E-06 | 1.34E-05 |
| BP | GO:0019080 | viral gene expression | 198/18862 | 2.59E-06 | 1.39E-05 |
| BP | GO:0034199 | activation of protein kinase A activity | 18/18862 | 3.26E-06 | 1.74E-05 |
| BP | GO:0040029 | regulation of gene expression, epigenetic | 205/18862 | 3.45E-06 | 1.83E-05 |
| BP | GO:0072529 | pyrimidine-containing compound catabolic process | 40/18862 | 3.60E-06 | 1.90E-05 |
| BP | GO:0009148 | pyrimidine nucleoside triphosphate biosynthetic process | 19/18862 | 4.11E-06 | 2.16E-05 |
| BP | GO:0019985 | translesion synthesis | 42/18862 | 4.61E-06 | 2.41E-05 |
| BP | GO:0000082 | G1/S transition of mitotic cell cycle | 275/18862 | 5.21E-06 | 2.70E-05 |
| BP | GO:0043434 | response to peptide hormone | 435/18862 | 9.77E-06 | 5.04E-05 |
| BP | GO:0044843 | cell cycle G1/S phase transition | 298/18862 | 1.05E-05 | 5.40E-05 |
| BP | GO:0042454 | ribonucleoside catabolic process | 24/18862 | 1.10E-05 | 5.62E-05 |
| BP | GO:0032648 | regulation of interferon-beta production | 50/18862 | 1.11E-05 | 5.62E-05 |
| BP | GO:0007589 | body fluid secretion | 86/18862 | 1.16E-05 | 5.87E-05 |
| BP | GO:0006301 | postreplication repair | 52/18862 | 1.34E-05 | 6.72E-05 |
| BP | GO:0032608 | interferon-beta production | 52/18862 | 1.34E-05 | 6.72E-05 |
| BP | GO:0019932 | second-messenger-mediated signaling | 307/18862 | 1.36E-05 | 6.77E-05 |
| BP | GO:0071377 | cellular response to glucagon stimulus | 26/18862 | 1.54E-05 | 7.60E-05 |
| BP | GO:0060249 | anatomical structure homeostasis | 466/18862 | 1.94E-05 | 9.53E-05 |
| BP | GO:0007189 | adenylate cyclase-activating G protein-coupled receptor signaling pathway | 143/18862 | 2.19E-05 | 0.000107034 |
| BP | GO:0071375 | cellular response to peptide hormone stimulus | 325/18862 | 2.22E-05 | 0.00010826 |
| BP | GO:0007168 | receptor guanylyl cyclase signaling pathway | 10/18862 | 2.31E-05 | 0.000111422 |
| BP | GO:0051607 | defense response to virus | 260/18862 | 2.32E-05 | 0.000111422 |
| BP | GO:0140546 | defense response to symbiont | 260/18862 | 2.32E-05 | 0.000111422 |
| BP | GO:0019934 | cGMP-mediated signaling | 30/18862 | 2.77E-05 | 0.000131929 |
| BP | GO:0006182 | cGMP biosynthetic process | 11/18862 | 3.16E-05 | 0.000150042 |
| BP | GO:0006296 | nucleotide-excision repair, DNA incision, 5'-to lesion | 37/18862 | 6.46E-05 | 0.00030186 |
| BP | GO:0033762 | response to glucagon | 37/18862 | 6.46E-05 | 0.00030186 |
| BP | GO:0043949 | regulation of cAMP-mediated signaling | 37/18862 | 6.46E-05 | 0.00030186 |
| BP | GO:0007188 | adenylate cyclase-modulating G protein-coupled receptor signaling pathway | 230/18862 | 6.50E-05 | 0.000302249 |
| BP | GO:0010752 | regulation of cGMP-mediated signaling | 14/18862 | 6.89E-05 | 0.000318677 |
| BP | GO:0031100 | animal organ regeneration | 73/18862 | 7.04E-05 | 0.000324251 |
| BP | GO:0003091 | renal water homeostasis | 38/18862 | 7.19E-05 | 0.000327657 |
| BP | GO:0006298 | mismatch repair | 38/18862 | 7.19E-05 | 0.000327657 |
| BP | GO:0007190 | activation of adenylate cyclase activity | 39/18862 | 7.97E-05 | 0.000358213 |
| BP | GO:0033683 | nucleotide-excision repair, DNA incision | 39/18862 | 7.97E-05 | 0.000358213 |
| BP | GO:0042769 | DNA damage response, detection of DNA damage | 39/18862 | 7.97E-05 | 0.000358213 |
| BP | GO:0046031 | ADP metabolic process | 122/18862 | 8.42E-05 | 0.000376702 |
| BP | GO:0046034 | ATP metabolic process | 313/18862 | 9.77E-05 | 0.000434723 |
| BP | GO:1901653 | cellular response to peptide | 391/18862 | 0.000104798 | 0.000464257 |
| BP | GO:0007193 | adenylate cyclase-inhibiting G protein-coupled receptor signaling pathway | 85/18862 | 0.000145413 | 0.000641145 |
| BP | GO:0007595 | lactation | 46/18862 | 0.000153215 | 0.000672376 |
| BP | GO:0009615 | response to virus | 359/18862 | 0.000272265 | 0.001189233 |
| BP | GO:0006359 | regulation of transcription by RNA polymerase III | 23/18862 | 0.00032238 | 0.001395095 |
| BP | GO:0006541 | glutamine metabolic process | 23/18862 | 0.00032238 | 0.001395095 |
| BP | GO:0006754 | ATP biosynthetic process | 56/18862 | 0.000329619 | 0.001419847 |
| BP | GO:0000377 | RNA splicing, via transesterification reactions with bulged adenosine as nucleophile | 383/18862 | 0.000436533 | 0.001863213 |
| BP | GO:0000398 | mRNA splicing, via spliceosome | 383/18862 | 0.000436533 | 0.001863213 |
| BP | GO:0000375 | RNA splicing, via transesterification reactions | 386/18862 | 0.000461852 | 0.001962317 |
| BP | GO:0071880 | adenylate cyclase-activating adrenergic receptor signaling pathway | 26/18862 | 0.000467244 | 0.001976247 |
| BP | GO:0003014 | renal system process | 118/18862 | 0.000665256 | 0.00280108 |
| BP | GO:0071875 | adrenergic receptor signaling pathway | 31/18862 | 0.000790701 | 0.003314338 |
| BP | GO:0050891 | multicellular organismal water homeostasis | 72/18862 | 0.000859021 | 0.003584637 |
| BP | GO:0046683 | response to organophosphorus | 130/18862 | 0.001029423 | 0.004276621 |
| BP | GO:0030104 | water homeostasis | 79/18862 | 0.001215661 | 0.005027979 |
| BP | GO:0042417 | dopamine metabolic process | 38/18862 | 0.001440242 | 0.005930606 |
| BP | GO:0009396 | folic acid-containing compound biosynthetic process | 10/18862 | 0.00149756 | 0.006086194 |
| BP | GO:1904321 | response to forskolin | 10/18862 | 0.00149756 | 0.006086194 |
| BP | GO:1904322 | cellular response to forskolin | 10/18862 | 0.00149756 | 0.006086194 |
| BP | GO:0014074 | response to purine-containing compound | 144/18862 | 0.001621201 | 0.006560158 |
| BP | GO:0014075 | response to amine | 42/18862 | 0.001926781 | 0.007729755 |
| BP | GO:0043114 | regulation of vascular permeability | 42/18862 | 0.001926781 | 0.007729755 |
| BP | GO:0008380 | RNA splicing | 481/18862 | 0.002149309 | 0.008488939 |
| BP | GO:0001973 | G protein-coupled adenosine receptor signaling pathway | 12/18862 | 0.002179593 | 0.008488939 |
| BP | GO:0035588 | G protein-coupled purinergic receptor signaling pathway | 12/18862 | 0.002179593 | 0.008488939 |
| BP | GO:0036005 | response to macrophage colony-stimulating factor | 12/18862 | 0.002179593 | 0.008488939 |
| BP | GO:0036006 | cellular response to macrophage colony-stimulating factor stimulus | 12/18862 | 0.002179593 | 0.008488939 |
| BP | GO:0071872 | cellular response to epinephrine stimulus | 12/18862 | 0.002179593 | 0.008488939 |
| BP | GO:1901077 | regulation of relaxation of muscle | 12/18862 | 0.002179593 | 0.008488939 |
| BP | GO:0032869 | cellular response to insulin stimulus | 226/18862 | 0.002204507 | 0.008550346 |
| BP | GO:0051591 | response to cAMP | 93/18862 | 0.00221835 | 0.008568484 |
| BP | GO:0016311 | dephosphorylation | 491/18862 | 0.002467964 | 0.009493401 |
| BP | GO:0086103 | G protein-coupled receptor signaling pathway involved in heart process | 14/18862 | 0.002982191 | 0.01142444 |
| BP | GO:0048732 | gland development | 413/18862 | 0.003065584 | 0.011695976 |
| BP | GO:0019674 | NAD metabolic process | 51/18862 | 0.003363832 | 0.012765311 |
| BP | GO:0042053 | regulation of dopamine metabolic process | 15/18862 | 0.003427802 | 0.012765311 |
| BP | GO:0042069 | regulation of catecholamine metabolic process | 15/18862 | 0.003427802 | 0.012765311 |
| BP | GO:0042559 | pteridine-containing compound biosynthetic process | 15/18862 | 0.003427802 | 0.012765311 |
| BP | GO:0043117 | positive regulation of vascular permeability | 15/18862 | 0.003427802 | 0.012765311 |
| BP | GO:0071871 | response to epinephrine | 15/18862 | 0.003427802 | 0.012765311 |
| BP | GO:0006584 | catecholamine metabolic process | 52/18862 | 0.003554635 | 0.013132998 |
| BP | GO:0009712 | catechol-containing compound metabolic process | 52/18862 | 0.003554635 | 0.013132998 |
| BP | GO:0007625 | grooming behavior | 16/18862 | 0.003902482 | 0.014361393 |
| BP | GO:0043116 | negative regulation of vascular permeability | 17/18862 | 0.004405879 | 0.016087254 |
| BP | GO:0055119 | relaxation of cardiac muscle | 17/18862 | 0.004405879 | 0.016087254 |
| BP | GO:0009223 | pyrimidine deoxyribonucleotide catabolic process | 18/18862 | 0.004937644 | 0.017958746 |
| BP | GO:0042493 | response to drug | 359/18862 | 0.005346965 | 0.019372111 |
| BP | GO:0046653 | tetrahydrofolate metabolic process | 19/18862 | 0.005497433 | 0.019840359 |
| BP | GO:0031099 | regeneration | 192/18862 | 0.005571362 | 0.020029835 |
| BP | GO:0046365 | monosaccharide catabolic process | 62/18862 | 0.005830229 | 0.020880191 |
| BP | GO:0032868 | response to insulin | 278/18862 | 0.006055728 | 0.021583367 |
| BP | GO:0006244 | pyrimidine nucleotide catabolic process | 20/18862 | 0.006084903 | 0.021583367 |
| BP | GO:0035690 | cellular response to drug | 63/18862 | 0.006095843 | 0.021583367 |
| BP | GO:0030879 | mammary gland development | 132/18862 | 0.007711113 | 0.027199477 |
| BP | GO:0009219 | pyrimidine deoxyribonucleotide metabolic process | 23/18862 | 0.008010024 | 0.02814761 |
| BP | GO:0001556 | oocyte maturation | 24/18862 | 0.008704857 | 0.030474721 |
| BP | GO:0001889 | liver development | 138/18862 | 0.008986811 | 0.031344415 |
| BP | GO:0034035 | purine ribonucleoside bisphosphate metabolic process | 25/18862 | 0.009425705 | 0.03256343 |
| BP | GO:0050427 | 3'-phosphoadenosine 5'-phosphosulfate metabolic process | 25/18862 | 0.009425705 | 0.03256343 |
| BP | GO:0061008 | hepaticobiliary system development | 140/18862 | 0.009440828 | 0.03256343 |
| BP | GO:0009064 | glutamine family amino acid metabolic process | 75/18862 | 0.009852186 | 0.033857359 |
| BP | GO:0010460 | positive regulation of heart rate | 26/18862 | 0.010172243 | 0.034829196 |
| BP | GO:0000288 | nuclear-transcribed mRNA catabolic process, deadenylation-dependent decay | 77/18862 | 0.010583116 | 0.036103754 |
| BP | GO:0006735 | NADH regeneration | 27/18862 | 0.01094415 | 0.036798203 |
| BP | GO:0006760 | folic acid-containing compound metabolic process | 27/18862 | 0.01094415 | 0.036798203 |
| BP | GO:0061621 | canonical glycolysis | 27/18862 | 0.01094415 | 0.036798203 |
| BP | GO:0061718 | glucose catabolic process to pyruvate | 27/18862 | 0.01094415 | 0.036798203 |
| BP | GO:0061620 | glycolytic process through glucose-6-phosphate | 28/18862 | 0.011741107 | 0.039336363 |
| BP | GO:0061615 | glycolytic process through fructose-6-phosphate | 29/18862 | 0.012562798 | 0.041938964 |
| BP | GO:0001975 | response to amphetamine | 30/18862 | 0.013408909 | 0.04428905 |
| BP | GO:0032743 | positive regulation of interleukin-2 production | 30/18862 | 0.013408909 | 0.04428905 |
| BP | GO:0045948 | positive regulation of translational initiation | 30/18862 | 0.013408909 | 0.04428905 |
| BP | GO:0090501 | RNA phosphodiester bond hydrolysis | 160/18862 | 0.014817079 | 0.048767851 |
| BP | GO:0008016 | regulation of heart contraction | 245/18862 | 0.014949749 | 0.049031862 |
| BP | GO:0035590 | purinergic nucleotide receptor signaling pathway | 32/18862 | 0.015173154 | 0.04941779 |
| BP | GO:0090075 | relaxation of muscle | 32/18862 | 0.015173154 | 0.04941779 |
| CC | GO:0061695 | transferase complex, transferring phosphorus-containing groups | 253/19520 | 4.97E-46 | 6.44E-44 |
| CC | GO:0055029 | nuclear DNA-directed RNA polymerase complex | 103/19520 | 2.65E-42 | 1.58E-40 |
| CC | GO:0000428 | DNA-directed RNA polymerase complex | 104/19520 | 3.66E-42 | 1.58E-40 |
| CC | GO:0030880 | RNA polymerase complex | 108/19520 | 1.28E-41 | 4.15E-40 |
| CC | GO:0005666 | RNA polymerase III complex | 18/19520 | 5.02E-35 | 1.30E-33 |
| CC | GO:0042575 | DNA polymerase complex | 20/19520 | 6.70E-23 | 1.45E-21 |
| CC | GO:0005665 | RNA polymerase II, core complex | 15/19520 | 1.46E-22 | 2.70E-21 |
| CC | GO:0005736 | RNA polymerase I complex | 13/19520 | 5.99E-21 | 9.69E-20 |
| CC | GO:0043601 | nuclear replisome | 22/19520 | 2.35E-13 | 3.38E-12 |
| CC | GO:0030894 | replisome | 24/19520 | 5.36E-13 | 6.94E-12 |
| CC | GO:0016591 | RNA polymerase II, holoenzyme | 79/19520 | 7.16E-13 | 8.43E-12 |
| CC | GO:0043596 | nuclear replication fork | 35/19520 | 1.63E-11 | 1.76E-10 |
| CC | GO:0005657 | replication fork | 65/19520 | 3.04E-09 | 3.03E-08 |
| CC | GO:0000228 | nuclear chromosome | 250/19520 | 1.73E-08 | 1.60E-07 |
| CC | GO:0032993 | protein-DNA complex | 208/19520 | 2.73E-06 | 2.36E-05 |
| CC | GO:0005671 | Ada2/Gcn5/Ada3 transcription activator complex | 15/19520 | 0.003149627 | 0.025487113 |
| CC | GO:0101002 | ficolin-1-rich granule | 185/19520 | 0.00397575 | 0.030279704 |
| CC | GO:1904813 | ficolin-1-rich granule lumen | 124/19520 | 0.005335251 | 0.03837637 |
| MF | GO:0016779 | nucleotidyltransferase activity | 131/18337 | 5.38E-51 | 4.19E-49 |
| MF | GO:0003899 | DNA-directed 5'-3' RNA polymerase activity | 39/18337 | 3.52E-47 | 1.37E-45 |
| MF | GO:0034062 | 5'-3' RNA polymerase activity | 43/18337 | 1.39E-45 | 2.71E-44 |
| MF | GO:0097747 | RNA polymerase activity | 43/18337 | 1.39E-45 | 2.71E-44 |
| MF | GO:0004112 | cyclic-nucleotide phosphodiesterase activity | 25/18337 | 1.83E-25 | 2.85E-24 |
| MF | GO:0004114 | 3',5'-cyclic-nucleotide phosphodiesterase activity | 23/18337 | 8.72E-24 | 1.13E-22 |
| MF | GO:0140098 | catalytic activity, acting on RNA | 386/18337 | 1.66E-22 | 1.84E-21 |
| MF | GO:0042578 | phosphoric ester hydrolase activity | 367/18337 | 1.20E-20 | 1.16E-19 |
| MF | GO:0008081 | phosphoric diester hydrolase activity | 89/18337 | 1.51E-19 | 1.31E-18 |
| MF | GO:0019205 | nucleobase-containing compound kinase activity | 42/18337 | 1.77E-19 | 1.38E-18 |
| MF | GO:0004115 | 3',5'-cyclic-AMP phosphodiesterase activity | 13/18337 | 5.97E-18 | 4.23E-17 |
| MF | GO:0016776 | phosphotransferase activity, phosphate group as acceptor | 39/18337 | 2.63E-14 | 1.71E-13 |
| MF | GO:0009975 | cyclase activity | 22/18337 | 4.48E-13 | 2.49E-12 |
| MF | GO:0016849 | phosphorus-oxygen lyase activity | 22/18337 | 4.48E-13 | 2.49E-12 |
| MF | GO:0003887 | DNA-directed DNA polymerase activity | 25/18337 | 1.49E-12 | 7.74E-12 |
| MF | GO:0004550 | nucleoside diphosphate kinase activity | 18/18337 | 7.90E-12 | 3.84E-11 |
| MF | GO:0034061 | DNA polymerase activity | 36/18337 | 3.95E-11 | 1.81E-10 |
| MF | GO:0008253 | 5'-nucleotidase activity | 13/18337 | 7.52E-11 | 3.25E-10 |
| MF | GO:0017110 | nucleoside-diphosphatase activity | 14/18337 | 1.31E-10 | 5.10E-10 |
| MF | GO:0047555 | 3',5'-cyclic-GMP phosphodiesterase activity | 14/18337 | 1.31E-10 | 5.10E-10 |
| MF | GO:0008252 | nucleotidase activity | 15/18337 | 2.17E-10 | 8.05E-10 |
| MF | GO:0000287 | magnesium ion binding | 216/18337 | 7.13E-10 | 2.53E-09 |
| MF | GO:0004016 | adenylate cyclase activity | 10/18337 | 1.91E-09 | 6.47E-09 |
| MF | GO:0016829 | lyase activity | 194/18337 | 2.90E-08 | 9.41E-08 |
| MF | GO:0050145 | nucleoside monophosphate kinase activity | 20/18337 | 1.12E-07 | 3.49E-07 |
| MF | GO:0047429 | nucleoside-triphosphate diphosphatase activity | 11/18337 | 4.21E-07 | 1.26E-06 |
| MF | GO:0030551 | cyclic nucleotide binding | 37/18337 | 2.90E-06 | 8.36E-06 |
| MF | GO:0051536 | iron-sulfur cluster binding | 67/18337 | 3.34E-06 | 8.98E-06 |
| MF | GO:0051540 | metal cluster binding | 67/18337 | 3.34E-06 | 8.98E-06 |
| MF | GO:0140097 | catalytic activity, acting on DNA | 204/18337 | 4.47E-06 | 1.16E-05 |
| MF | GO:0001882 | nucleoside binding | 390/18337 | 4.72E-06 | 1.18E-05 |
| MF | GO:0051539 | 4 iron, 4 sulfur cluster binding | 42/18337 | 5.52E-06 | 1.32E-05 |
| MF | GO:0019001 | guanyl nucleotide binding | 398/18337 | 5.80E-06 | 1.32E-05 |
| MF | GO:0032561 | guanyl ribonucleotide binding | 398/18337 | 5.80E-06 | 1.32E-05 |
| MF | GO:0004551 | nucleotide diphosphatase activity | 20/18337 | 5.93E-06 | 1.32E-05 |
| MF | GO:0032549 | ribonucleoside binding | 384/18337 | 2.33E-05 | 5.04E-05 |
| MF | GO:0016791 | phosphatase activity | 276/18337 | 4.95E-05 | 0.000104136 |
| MF | GO:0003697 | single-stranded DNA binding | 116/18337 | 7.80E-05 | 0.00015997 |
| MF | GO:0030553 | cGMP binding | 15/18337 | 9.57E-05 | 0.000191081 |
| MF | GO:0001883 | purine nucleoside binding | 384/18337 | 0.000122534 | 0.000238619 |
| MF | GO:0004527 | exonuclease activity | 82/18337 | 0.000145834 | 0.000277067 |
| MF | GO:0016879 | ligase activity, forming carbon-nitrogen bonds | 47/18337 | 0.000192118 | 0.00035631 |
| MF | GO:0004518 | nuclease activity | 206/18337 | 0.00027284 | 0.00049425 |
| MF | GO:0030552 | cAMP binding | 23/18337 | 0.00035935 | 0.000636169 |
| MF | GO:0032550 | purine ribonucleoside binding | 381/18337 | 0.000549389 | 0.000948403 |
| MF | GO:0030145 | manganese ion binding | 62/18337 | 0.00056007 | 0.000948403 |
| MF | GO:0016814 | hydrolase activity, acting on carbon-nitrogen (but not peptide) bonds, in cyclic amidines | 35/18337 | 0.001259137 | 0.002086811 |
| MF | GO:0016884 | carbon-nitrogen ligase activity, with glutamine as amido-N-donor | 10/18337 | 0.001611459 | 0.002615086 |
| MF | GO:0008296 | 3'-5'-exodeoxyribonuclease activity | 11/18337 | 0.001961712 | 0.003056141 |
| MF | GO:0030955 | potassium ion binding | 11/18337 | 0.001961712 | 0.003056141 |
| MF | GO:0008179 | adenylate cyclase binding | 12/18337 | 0.002344679 | 0.00358114 |
| MF | GO:0016763 | transferase activity, transferring pentosyl groups | 50/18337 | 0.003528621 | 0.005285789 |
| MF | GO:0016208 | AMP binding | 15/18337 | 0.003685818 | 0.005316774 |
| MF | GO:0019215 | intermediate filament binding | 15/18337 | 0.003685818 | 0.005316774 |
| MF | GO:0031420 | alkali metal ion binding | 16/18337 | 0.004195617 | 0.005942118 |
| MF | GO:0008408 | 3'-5' exonuclease activity | 55/18337 | 0.004620102 | 0.006426458 |
| MF | GO:0016796 | exonuclease activity, active with either ribo- or deoxyribonucleic acids and producing 5'-phosphomonoesters | 57/18337 | 0.005107604 | 0.00697992 |
| MF | GO:0004536 | deoxyribonuclease activity | 59/18337 | 0.00562498 | 0.007554419 |
| MF | GO:0016810 | hydrolase activity, acting on carbon-nitrogen (but not peptide) bonds | 117/18337 | 0.005760907 | 0.007605837 |
| MF | GO:0004529 | exodeoxyribonuclease activity | 22/18337 | 0.007886128 | 0.010070293 |
| MF | GO:0016895 | exodeoxyribonuclease activity, producing 5'-phosphomonoesters | 22/18337 | 0.007886128 | 0.010070293 |
| MF | GO:0005525 | GTP binding | 376/18337 | 0.008304358 | 0.010433319 |
| MF | GO:0043015 | gamma-tubulin binding | 28/18337 | 0.012601158 | 0.015580379 |
| MF | GO:0042562 | hormone binding | 84/18337 | 0.014774083 | 0.017981614 |
| MF | GO:0019239 | deaminase activity | 32/18337 | 0.016275263 | 0.018921751 |
| MF | GO:0030515 | snoRNA binding | 32/18337 | 0.016275263 | 0.018921751 |
| MF | GO:0031369 | translation initiation factor binding | 32/18337 | 0.016275263 | 0.018921751 |
| MF | GO:0003727 | single-stranded RNA binding | 88/18337 | 0.016720126 | 0.019153086 |
| MF | GO:0016874 | ligase activity | 163/18337 | 0.017810872 | 0.020106858 |
| MF | GO:0016831 | carboxy-lyase activity | 35/18337 | 0.019293801 | 0.021469793 |
| MF | GO:0043531 | ADP binding | 39/18337 | 0.023652115 | 0.025948947 |
| MF | GO:0017046 | peptide hormone binding | 50/18337 | 0.037447427 | 0.040513298 |
| MF | GO:0016830 | carbon-carbon lyase activity | 51/18337 | 0.03882354 | 0.041426705 |

**Table 7b. KEGG enrichment analysis.**

| ID | Description | BgRatio | pvalue | qvalue |
| --- | --- | --- | --- | --- |
| hsa00230 | Purine metabolism | 128/8165 | 2.80E-106 | 1.59E-104 |
| hsa01232 | Nucleotide metabolism | 85/8165 | 1.67E-48 | 4.74E-47 |
| hsa03020 | RNA polymerase | 34/8165 | 7.38E-41 | 1.40E-39 |
| hsa00240 | Pyrimidine metabolism | 58/8165 | 5.08E-22 | 7.21E-21 |
| hsa04623 | Cytosolic DNA-sensing pathway | 63/8165 | 1.12E-16 | 1.28E-15 |
| hsa03030 | DNA replication | 36/8165 | 2.31E-14 | 2.19E-13 |
| hsa05032 | Morphine addiction | 91/8165 | 1.01E-12 | 8.24E-12 |
| hsa00983 | Drug metabolism - other enzymes | 80/8165 | 9.34E-09 | 6.42E-08 |
| hsa03410 | Base excision repair | 33/8165 | 1.02E-08 | 6.42E-08 |
| hsa03420 | Nucleotide excision repair | 47/8165 | 1.96E-07 | 1.12E-06 |
| hsa00760 | Nicotinate and nicotinamide metabolism | 36/8165 | 8.11E-06 | 4.19E-05 |
| hsa04924 | Renin secretion | 69/8165 | 4.05E-05 | 0.000177847 |
| hsa00730 | Thiamine metabolism | 15/8165 | 4.08E-05 | 0.000177847 |
| hsa01240 | Biosynthesis of cofactors | 153/8165 | 4.38E-05 | 0.000177847 |
| hsa04022 | cGMP-PKG signaling pathway | 167/8165 | 9.19E-05 | 0.000348298 |
| hsa04923 | Regulation of lipolysis in adipocytes | 58/8165 | 0.000131953 | 0.00046878 |
| hsa04024 | cAMP signaling pathway | 221/8165 | 0.000211877 | 0.000708442 |
| hsa03430 | Mismatch repair | 23/8165 | 0.000242939 | 0.000767176 |
| hsa04928 | Parathyroid hormone synthesis, secretion and action | 106/8165 | 0.00060379 | 0.001806352 |
| hsa04925 | Aldosterone synthesis and secretion | 98/8165 | 0.002203294 | 0.006221123 |
| hsa03440 | Homologous recombination | 41/8165 | 0.002298359 | 0.006221123 |
| hsa05016 | Huntington disease | 306/8165 | 0.003083236 | 0.007966255 |
| hsa04913 | Ovarian steroidogenesis | 51/8165 | 0.005107315 | 0.012622198 |
| hsa04270 | Vascular smooth muscle contraction | 134/8165 | 0.010112927 | 0.023024853 |
| hsa04213 | Longevity regulating pathway - multiple species | 62/8165 | 0.010164705 | 0.023024853 |
| hsa04713 | Circadian entrainment | 97/8165 | 0.010531738 | 0.023024853 |
| hsa04927 | Cortisol synthesis and secretion | 65/8165 | 0.01195754 | 0.024314873 |
| hsa04371 | Apelin signaling pathway | 139/8165 | 0.011977326 | 0.024314873 |
| hsa04914 | Progesterone-mediated oocyte maturation | 102/8165 | 0.012900826 | 0.025286556 |
| hsa04921 | Oxytocin signaling pathway | 154/8165 | 0.019007263 | 0.035483169 |
| hsa04918 | Thyroid hormone synthesis | 75/8165 | 0.019351469 | 0.035483169 |
| hsa04971 | Gastric acid secretion | 76/8165 | 0.020216268 | 0.035910477 |
| hsa04935 | Growth hormone synthesis, secretion and action | 120/8165 | 0.024353195 | 0.041948088 |
| hsa00670 | One carbon pool by folate | 20/8165 | 0.030166559 | 0.04664583 |
| hsa04742 | Taste transduction | 86/8165 | 0.030179959 | 0.04664583 |
| hsa04911 | Insulin secretion | 86/8165 | 0.030179959 | 0.04664583 |
| hsa04540 | Gap junction | 88/8165 | 0.032465537 | 0.04664583 |
| hsa00770 | Pantothenate and CoA biosynthesis | 21/8165 | 0.033048184 | 0.04664583 |
| hsa04211 | Longevity regulating pathway | 89/8165 | 0.033645464 | 0.04664583 |
| hsa04727 | GABAergic synapse | 89/8165 | 0.033645464 | 0.04664583 |
| hsa04976 | Bile secretion | 89/8165 | 0.033645464 | 0.04664583 |

**Appendix 10**

**gene set enrichment analyses (GSEA)**

**Table 8a. GSEA of high rish.**

| NAME | SIZE | ES | NES | NOM p-val | FDR q-val |
| --- | --- | --- | --- | --- | --- |
| KEGG_FOCAL_ADHESION | 199 | 0.6476552 | 2.3587148 | 0 | 0 |
| KEGG_ECM_RECEPTOR_INTERACTION | 84 | 0.7297853 | 2.2827163 | 0 | 0 |
| KEGG_HYPERTROPHIC_CARDIOMYOPATHY_HCM | 83 | 0.6739827 | 2.25665 | 0 | 0 |
| KEGG_DILATED_CARDIOMYOPATHY | 90 | 0.6563518 | 2.2496815 | 0 | 3.26E-04 |
| KEGG_ARRHYTHMOGENIC_RIGHT_VENTRICULAR_CARDIOMYOPATHY_ARVC | 74 | 0.65538347 | 2.19847 | 0 | 8.76E-04 |
| KEGG_REGULATION_OF_ACTIN_CYTOSKELETON | 213 | 0.5651081 | 2.1939614 | 0 | 7.30E-04 |
| KEGG_MELANOMA | 71 | 0.6035899 | 2.1793203 | 0 | 7.44E-04 |
| KEGG_CYTOKINE_CYTOKINE_RECEPTOR_INTERACTION | 264 | 0.5867292 | 2.1346319 | 0 | 0.001805137 |
| KEGG_COMPLEMENT_AND_COAGULATION_CASCADES | 69 | 0.67470324 | 2.1027026 | 0.002040816 | 0.002401257 |
| KEGG_NOD_LIKE_RECEPTOR_SIGNALING_PATHWAY | 62 | 0.60374904 | 2.0300221 | 0.006085193 | 0.007559298 |
| KEGG_GLYCOSAMINOGLYCAN_BIOSYNTHESIS_CHONDROITIN_SULFATE | 22 | 0.7352404 | 2.0292728 | 0 | 0.006975394 |
| KEGG_GAP_JUNCTION | 90 | 0.54247814 | 2.0247746 | 0.002 | 0.007530927 |
| KEGG_VIRAL_MYOCARDITIS | 68 | 0.66202694 | 2.0167093 | 0 | 0.008186874 |
| KEGG_PATHWAYS_IN_CANCER | 325 | 0.47564045 | 2.0123951 | 0 | 0.008091019 |
| KEGG_CALCIUM_SIGNALING_PATHWAY | 178 | 0.4935515 | 1.9637626 | 0.002079002 | 0.013382114 |
| KEGG_CELL_ADHESION_MOLECULES_CAMS | 131 | 0.5821723 | 1.9602876 | 0.00998004 | 0.013164851 |
| KEGG_MELANOGENESIS | 101 | 0.49961072 | 1.9530722 | 0.00203252 | 0.013502355 |
| KEGG_PATHOGENIC_ESCHERICHIA_COLI_INFECTION | 56 | 0.5709074 | 1.9345485 | 0.002057613 | 0.016161595 |
| KEGG_WNT_SIGNALING_PATHWAY | 151 | 0.4796536 | 1.927668 | 0 | 0.016424933 |
| KEGG_LEISHMANIA_INFECTION | 70 | 0.6376505 | 1.9214228 | 0.015655577 | 0.016243324 |
| KEGG_CHEMOKINE_SIGNALING_PATHWAY | 188 | 0.5254078 | 1.917269 | 0.012195122 | 0.016020415 |
| KEGG_GLIOMA | 65 | 0.5216135 | 1.907154 | 0.003960396 | 0.01703244 |
| KEGG_MAPK_SIGNALING_PATHWAY | 267 | 0.44702625 | 1.8801389 | 0.001976285 | 0.022012113 |
| KEGG_RENIN_ANGIOTENSIN_SYSTEM | 17 | 0.684217 | 1.8742491 | 0.004219409 | 0.02193081 |
| KEGG_HEMATOPOIETIC_CELL_LINEAGE | 85 | 0.6276564 | 1.8680438 | 0.014403292 | 0.021992952 |
| KEGG_ACUTE_MYELOID_LEUKEMIA | 57 | 0.5323443 | 1.8639356 | 0.004166667 | 0.022111472 |
| KEGG_PRION_DISEASES | 35 | 0.5994577 | 1.852381 | 0.004065041 | 0.024435151 |
| KEGG_LEUKOCYTE_TRANSENDOTHELIAL_MIGRATION | 116 | 0.49949536 | 1.8444147 | 0.004132231 | 0.02552428 |
| KEGG_VASCULAR_SMOOTH_MUSCLE_CONTRACTION | 115 | 0.49270564 | 1.836324 | 0.004106776 | 0.026392955 |
| KEGG_SMALL_CELL_LUNG_CANCER | 84 | 0.5068021 | 1.8227104 | 0.01183432 | 0.02872633 |
| KEGG_PANCREATIC_CANCER | 70 | 0.49452564 | 1.7990465 | 0.00990099 | 0.034377463 |
| KEGG_RENAL_CELL_CARCINOMA | 70 | 0.49358043 | 1.7902948 | 0.015841585 | 0.03529477 |
| KEGG_ALLOGRAFT_REJECTION | 35 | 0.761138 | 1.7877699 | 0.021276595 | 0.03481406 |
| KEGG_NEUROACTIVE_LIGAND_RECEPTOR_INTERACTION | 272 | 0.43834627 | 1.7785578 | 0 | 0.036705583 |
| KEGG_JAK_STAT_SIGNALING_PATHWAY | 155 | 0.46041235 | 1.7605431 | 0.011764706 | 0.04089989 |
| KEGG_OOCYTE_MEIOSIS | 113 | 0.4936886 | 1.7583714 | 0.018072288 | 0.04050501 |
| KEGG_PROGESTERONE_MEDIATED_OOCYTE_MATURATION | 85 | 0.49459055 | 1.7560087 | 0.01632653 | 0.04017899 |
| KEGG_AUTOIMMUNE_THYROID_DISEASE | 50 | 0.6271997 | 1.7494363 | 0.04263566 | 0.041076906 |
| KEGG_ADHERENS_JUNCTION | 73 | 0.47597402 | 1.746465 | 0.01814516 | 0.041181836 |
| KEGG_TOLL_LIKE_RECEPTOR_SIGNALING_PATHWAY | 102 | 0.4726971 | 1.7358736 | 0.01814516 | 0.043357868 |
| KEGG_AXON_GUIDANCE | 129 | 0.44513237 | 1.7238455 | 0.009940358 | 0.046484873 |
| KEGG_TGF_BETA_SIGNALING_PATHWAY | 86 | 0.45135763 | 1.7119277 | 0.011904762 | 0.049359947 |
| KEGG_BLADDER_CANCER | 42 | 0.49490622 | 1.7070447 | 0.008350731 | 0.049938023 |
| KEGG_O_GLYCAN_BIOSYNTHESIS | 30 | 0.5454836 | 1.6784239 | 0.03206413 | 0.059748825 |
| KEGG_PROTEASOME | 46 | 0.62279195 | 1.6729017 | 0.050485436 | 0.06011664 |
| KEGG_LONG_TERM_POTENTIATION | 70 | 0.4537139 | 1.6644652 | 0.01814516 | 0.061615452 |
| KEGG_GRAFT_VERSUS_HOST_DISEASE | 37 | 0.71909297 | 1.6616324 | 0.054 | 0.061480176 |
| KEGG_NATURAL_KILLER_CELL_MEDIATED_CYTOTOXICITY | 132 | 0.47953925 | 1.6607195 | 0.038306452 | 0.06079806 |
| KEGG_CHRONIC_MYELOID_LEUKEMIA | 73 | 0.4509745 | 1.6550096 | 0.034343433 | 0.06166143 |
| KEGG_NEUROTROPHIN_SIGNALING_PATHWAY | 126 | 0.42928237 | 1.6474732 | 0.026476579 | 0.06331403 |
| KEGG_BASAL_CELL_CARCINOMA | 55 | 0.4628864 | 1.6244329 | 0.023809524 | 0.07108297 |
| KEGG_ASTHMA | 28 | 0.6342366 | 1.6146822 | 0.09393346 | 0.07366732 |
| KEGG_COLORECTAL_CANCER | 62 | 0.4527929 | 1.6084965 | 0.03941909 | 0.07439589 |
| KEGG_EPITHELIAL_CELL_SIGNALING_IN_HELICOBACTER_PYLORI_INFECTION | 68 | 0.43966338 | 1.6019952 | 0.019723866 | 0.075358205 |
| KEGG_HEDGEHOG_SIGNALING_PATHWAY | 56 | 0.43543902 | 1.5829576 | 0.010504202 | 0.08239447 |
| KEGG_TYPE_I_DIABETES_MELLITUS | 41 | 0.6112285 | 1.5751454 | 0.09765625 | 0.08403598 |
| KEGG_TIGHT_JUNCTION | 131 | 0.38178927 | 1.5577933 | 0.041666668 | 0.09144766 |
| KEGG_SYSTEMIC_LUPUS_ERYTHEMATOSUS | 137 | 0.49454588 | 1.5332227 | 0.06324111 | 0.10324756 |
| KEGG_INTESTINAL_IMMUNE_NETWORK_FOR_IGA_PRODUCTION | 46 | 0.5875405 | 1.5220956 | 0.11198428 | 0.10765146 |
| KEGG_T_CELL_RECEPTOR_SIGNALING_PATHWAY | 108 | 0.44147354 | 1.5194709 | 0.09054326 | 0.10722647 |
| KEGG_GNRH_SIGNALING_PATHWAY | 101 | 0.36574957 | 1.5176643 | 0.030360531 | 0.10650706 |
| KEGG_GLYCOSAMINOGLYCAN_DEGRADATION | 21 | 0.505942 | 1.5155034 | 0.05338809 | 0.10608971 |
| KEGG_GLYCOSAMINOGLYCAN_BIOSYNTHESIS_KERATAN_SULFATE | 15 | 0.52920794 | 1.5035491 | 0.06138614 | 0.11181389 |
| KEGG_PROSTATE_CANCER | 89 | 0.39561024 | 1.4946076 | 0.06175299 | 0.115314156 |
| KEGG_FC_GAMMA_R_MEDIATED_PHAGOCYTOSIS | 96 | 0.38876724 | 1.4738575 | 0.078313254 | 0.12637375 |
| KEGG_ANTIGEN_PROCESSING_AND_PRESENTATION | 81 | 0.5054848 | 1.4668698 | 0.12974052 | 0.128816 |
| KEGG_CELL_CYCLE | 125 | 0.45260364 | 1.4190221 | 0.16564417 | 0.1606433 |
| KEGG_PURINE_METABOLISM | 159 | 0.35434112 | 1.4183502 | 0.07942974 | 0.15874912 |
| KEGG_NUCLEOTIDE_EXCISION_REPAIR | 44 | 0.50272584 | 1.4070519 | 0.15927419 | 0.16483252 |
| KEGG_GALACTOSE_METABOLISM | 25 | 0.45315564 | 1.3840203 | 0.10204082 | 0.1803119 |
| KEGG_RIG_I_LIKE_RECEPTOR_SIGNALING_PATHWAY | 71 | 0.39272803 | 1.3815867 | 0.115384616 | 0.17954509 |
| KEGG_LONG_TERM_DEPRESSION | 70 | 0.36549655 | 1.3805428 | 0.084645666 | 0.17804928 |
| KEGG_GLYCOSAMINOGLYCAN_BIOSYNTHESIS_HEPARAN_SULFATE | 26 | 0.42012417 | 1.3674879 | 0.1244898 | 0.18607217 |
| KEGG_SNARE_INTERACTIONS_IN_VESICULAR_TRANSPORT | 38 | 0.3906212 | 1.3497537 | 0.12865497 | 0.19740579 |
| KEGG_AMINO_SUGAR_AND_NUCLEOTIDE_SUGAR_METABOLISM | 44 | 0.40625954 | 1.346359 | 0.13636364 | 0.19739522 |
| KEGG_GLYCOSPHINGOLIPID_BIOSYNTHESIS_LACTO_AND_NEOLACTO_SERIES | 26 | 0.41377443 | 1.3427628 | 0.10878661 | 0.19791345 |
| KEGG_NICOTINATE_AND_NICOTINAMIDE_METABOLISM | 24 | 0.40842858 | 1.3322366 | 0.13279678 | 0.2036081 |
| KEGG_ENDOMETRIAL_CANCER | 52 | 0.3895145 | 1.3317512 | 0.14579055 | 0.20153116 |
| KEGG_APOPTOSIS | 87 | 0.3646811 | 1.3288031 | 0.14087301 | 0.20118092 |
| KEGG_P53_SIGNALING_PATHWAY | 68 | 0.35562557 | 1.3171111 | 0.13438736 | 0.20874722 |
| KEGG_CYTOSOLIC_DNA_SENSING_PATHWAY | 55 | 0.3994451 | 1.3132508 | 0.15992293 | 0.2092106 |
| KEGG_LYSOSOME | 121 | 0.37107342 | 1.3127766 | 0.16945606 | 0.207086 |
| KEGG_GLYCOLYSIS_GLUCONEOGENESIS | 61 | 0.37344766 | 1.3038081 | 0.13872832 | 0.21216902 |
| KEGG_MISMATCH_REPAIR | 23 | 0.5423658 | 1.294374 | 0.22937626 | 0.21815103 |
| KEGG_TYPE_II_DIABETES_MELLITUS | 47 | 0.36756513 | 1.2914011 | 0.13565892 | 0.21805218 |
| KEGG_GLYOXYLATE_AND_DICARBOXYLATE_METABOLISM | 16 | 0.49976486 | 1.2890611 | 0.19444445 | 0.21740805 |
| KEGG_ERBB_SIGNALING_PATHWAY | 87 | 0.3417738 | 1.2870888 | 0.17373738 | 0.21673746 |
| KEGG_ARGININE_AND_PROLINE_METABOLISM | 54 | 0.35566992 | 1.2847086 | 0.14059407 | 0.2163346 |
| KEGG_ALDOSTERONE_REGULATED_SODIUM_REABSORPTION | 42 | 0.37681037 | 1.2816207 | 0.14202334 | 0.21624856 |
| KEGG_NON_SMALL_CELL_LUNG_CANCER | 54 | 0.36281946 | 1.2664268 | 0.186722 | 0.22689994 |
| KEGG_INSULIN_SIGNALING_PATHWAY | 136 | 0.31246114 | 1.2605304 | 0.16666667 | 0.22924289 |
| KEGG_PRIMARY_BILE_ACID_BIOSYNTHESIS | 16 | 0.46421972 | 1.2598349 | 0.18629551 | 0.22732843 |
| KEGG_VIBRIO_CHOLERAE_INFECTION | 54 | 0.36527896 | 1.2531081 | 0.18292683 | 0.23058547 |
| KEGG_B_CELL_RECEPTOR_SIGNALING_PATHWAY | 75 | 0.38859943 | 1.2409338 | 0.23790322 | 0.23933752 |
| KEGG_PENTOSE_PHOSPHATE_PATHWAY | 27 | 0.40667054 | 1.2123903 | 0.25338492 | 0.26391783 |
| KEGG_TRYPTOPHAN_METABOLISM | 40 | 0.37328127 | 1.2120453 | 0.21161826 | 0.26145363 |
| KEGG_SPHINGOLIPID_METABOLISM | 39 | 0.3550977 | 1.2071719 | 0.22478992 | 0.26381895 |
| KEGG_DNA_REPLICATION | 36 | 0.5003353 | 1.1945876 | 0.334 | 0.27300096 |
| KEGG_PYRIMIDINE_METABOLISM | 98 | 0.3313719 | 1.1792989 | 0.2729045 | 0.28599286 |
| KEGG_AMYOTROPHIC_LATERAL_SCLEROSIS_ALS | 53 | 0.3131808 | 1.168088 | 0.23373984 | 0.29424316 |
| KEGG_BASAL_TRANSCRIPTION_FACTORS | 35 | 0.38810617 | 1.1680351 | 0.28683692 | 0.29135063 |
| KEGG_ADIPOCYTOKINE_SIGNALING_PATHWAY | 66 | 0.32225302 | 1.1466768 | 0.25963488 | 0.31026936 |
| KEGG_STARCH_AND_SUCROSE_METABOLISM | 51 | 0.32619452 | 1.1459957 | 0.25882354 | 0.30803227 |
| KEGG_GLYCOSPHINGOLIPID_BIOSYNTHESIS_GANGLIO_SERIES | 15 | 0.4115999 | 1.1386511 | 0.29795918 | 0.3130558 |
| KEGG_FC_EPSILON_RI_SIGNALING_PATHWAY | 79 | 0.29638174 | 1.1365849 | 0.31558186 | 0.31214935 |
| KEGG_RIBOFLAVIN_METABOLISM | 16 | 0.39630738 | 1.1314269 | 0.296 | 0.3142374 |
| KEGG_MTOR_SIGNALING_PATHWAY | 52 | 0.30999422 | 1.1242348 | 0.299389 | 0.31869173 |
| KEGG_PRIMARY_IMMUNODEFICIENCY | 35 | 0.45554015 | 1.1096376 | 0.35586482 | 0.33097935 |
| KEGG_DRUG_METABOLISM_OTHER_ENZYMES | 51 | 0.31045854 | 1.0837259 | 0.34816247 | 0.3558499 |
| KEGG_FRUCTOSE_AND_MANNOSE_METABOLISM | 34 | 0.32517695 | 1.0489041 | 0.412 | 0.393247 |
| KEGG_ONE_CARBON_POOL_BY_FOLATE | 17 | 0.3856233 | 1.046965 | 0.3944773 | 0.3922797 |
| KEGG_ABC_TRANSPORTERS | 44 | 0.29472578 | 1.0414505 | 0.38477367 | 0.39572585 |
| KEGG_LYSINE_DEGRADATION | 44 | 0.32642642 | 1.0383449 | 0.40365112 | 0.39608392 |
| KEGG_NOTCH_SIGNALING_PATHWAY | 47 | 0.29116657 | 1.0283308 | 0.4338843 | 0.40444013 |
| KEGG_PYRUVATE_METABOLISM | 40 | 0.31815672 | 1.012003 | 0.43346775 | 0.42029017 |
| KEGG_DORSO_VENTRAL_AXIS_FORMATION | 24 | 0.3267135 | 0.9986891 | 0.4347826 | 0.43308008 |
| KEGG_THYROID_CANCER | 29 | 0.29953015 | 0.991076 | 0.46588695 | 0.43881568 |
| KEGG_PANTOTHENATE_AND_COA_BIOSYNTHESIS | 16 | 0.32907787 | 0.9493192 | 0.5040816 | 0.48949215 |
| KEGG_PROTEIN_EXPORT | 24 | 0.3323486 | 0.9385577 | 0.53252035 | 0.4994019 |
| KEGG_STEROID_HORMONE_BIOSYNTHESIS | 55 | 0.26840368 | 0.9310073 | 0.5390946 | 0.5063065 |
| KEGG_CITRATE_CYCLE_TCA_CYCLE | 31 | 0.3465604 | 0.91333216 | 0.54545456 | 0.5261602 |
| KEGG_AMINOACYL_TRNA_BIOSYNTHESIS | 41 | 0.30538097 | 0.8811297 | 0.52871287 | 0.5683824 |
| KEGG_STEROID_BIOSYNTHESIS | 17 | 0.29161704 | 0.7332462 | 0.7476809 | 0.78191894 |
| KEGG_MATURITY_ONSET_DIABETES_OF_THE_YOUNG | 25 | 0.26891187 | 0.69555926 | 0.85071576 | 0.82435805 |
| KEGG_TASTE_TRANSDUCTION | 51 | 0.22210543 | 0.69063514 | 0.8782435 | 0.8238359 |

**Table 8b. GSEA of low rish.**

| NAME | SIZE | ES | NES | NOM p-val | FDR q-val |
| --- | --- | --- | --- | --- | --- |
| KEGG_PEROXISOME | 78 | -0.5765119 | -2.0248337 | 0 | 0.04480083 |
| KEGG_ALPHA_LINOLENIC_ACID_METABOLISM | 19 | -0.6762739 | -1.9579917 | 0.004016064 | 0.04714387 |
| KEGG_PARKINSONS_DISEASE | 130 | -0.589184 | -1.9492227 | 0.00984252 | 0.03570184 |
| KEGG_GLYCEROPHOSPHOLIPID_METABOLISM | 76 | -0.4880469 | -1.9485425 | 0 | 0.027072692 |
| KEGG_OXIDATIVE_PHOSPHORYLATION | 132 | -0.6005878 | -1.9215146 | 0.017274473 | 0.027205234 |
| KEGG_LINOLEIC_ACID_METABOLISM | 29 | -0.6073212 | -1.8970006 | 0.007920792 | 0.029862836 |
| KEGG_VALINE_LEUCINE_AND_ISOLEUCINE_DEGRADATION | 44 | -0.5936589 | -1.8072033 | 0.01443299 | 0.05454541 |
| KEGG_FATTY_ACID_METABOLISM | 42 | -0.54337823 | -1.800713 | 0.003992016 | 0.050156087 |
| KEGG_RIBOSOME | 88 | -0.7824908 | -1.7576834 | 0.027504912 | 0.06331224 |
| KEGG_ETHER_LIPID_METABOLISM | 33 | -0.5224973 | -1.7454667 | 0.010080645 | 0.0630704 |
| KEGG_HUNTINGTONS_DISEASE | 182 | -0.4569181 | -1.7099185 | 0.031496063 | 0.076147676 |
| KEGG_GLYCOSYLPHOSPHATIDYLINOSITOL_GPI_ANCHOR_BIOSYNTHESIS | 25 | -0.57333773 | -1.6905304 | 0.0234375 | 0.08060847 |
| KEGG_PPAR_SIGNALING_PATHWAY | 69 | -0.4496032 | -1.5957383 | 0.019762846 | 0.13776089 |
| KEGG_ALZHEIMERS_DISEASE | 166 | -0.41804948 | -1.5875816 | 0.053359684 | 0.13404785 |
| KEGG_CARDIAC_MUSCLE_CONTRACTION | 79 | -0.45426527 | -1.5534707 | 0.057539683 | 0.15258697 |
| KEGG_GLYCEROLIPID_METABOLISM | 49 | -0.40906706 | -1.5181943 | 0.029045643 | 0.1742368 |
| KEGG_ARACHIDONIC_ACID_METABOLISM | 58 | -0.41951063 | -1.5106231 | 0.045454547 | 0.17118123 |
| KEGG_DRUG_METABOLISM_CYTOCHROME_P450 | 71 | -0.42068946 | -1.4746875 | 0.059670784 | 0.1948373 |
| KEGG_BUTANOATE_METABOLISM | 34 | -0.438849 | -1.4300116 | 0.0877551 | 0.22867732 |
| KEGG_ALANINE_ASPARTATE_AND_GLUTAMATE_METABOLISM | 32 | -0.43168792 | -1.420278 | 0.08232932 | 0.22739455 |
| KEGG_RETINOL_METABOLISM | 64 | -0.39255285 | -1.3964615 | 0.08350305 | 0.24080274 |
| KEGG_METABOLISM_OF_XENOBIOTICS_BY_CYTOCHROME_P450 | 69 | -0.40973642 | -1.3786833 | 0.11434511 | 0.24783823 |
| KEGG_SPLICEOSOME | 127 | -0.44809657 | -1.3601053 | 0.187251 | 0.2575759 |
| KEGG_SELENOAMINO_ACID_METABOLISM | 26 | -0.4303671 | -1.3091121 | 0.18461539 | 0.30768555 |
| KEGG_GLUTATHIONE_METABOLISM | 49 | -0.38372266 | -1.2747691 | 0.18162839 | 0.3381019 |
| KEGG_VEGF_SIGNALING_PATHWAY | 76 | -0.31785947 | -1.2628822 | 0.16205534 | 0.34074223 |
| KEGG_RNA_DEGRADATION | 59 | -0.37768197 | -1.2315263 | 0.23505977 | 0.36890545 |
| KEGG_GLYCINE_SERINE_AND_THREONINE_METABOLISM | 31 | -0.38419732 | -1.1904689 | 0.27671754 | 0.41381437 |
| KEGG_PROPANOATE_METABOLISM | 33 | -0.3944007 | -1.1834431 | 0.28131416 | 0.40971503 |
| KEGG_ENDOCYTOSIS | 181 | -0.2825495 | -1.1648552 | 0.24902724 | 0.42367366 |
| KEGG_TERPENOID_BACKBONE_BIOSYNTHESIS | 15 | -0.45335937 | -1.1483628 | 0.34008098 | 0.43410438 |
| KEGG_PENTOSE_AND_GLUCURONATE_INTERCONVERSIONS | 28 | -0.38143802 | -1.1379116 | 0.30290458 | 0.4355307 |
| KEGG_OTHER_GLYCAN_DEGRADATION | 16 | -0.43415207 | -1.1260742 | 0.3138833 | 0.43865126 |
| KEGG_PHOSPHATIDYLINOSITOL_SIGNALING_SYSTEM | 76 | -0.30202642 | -1.1224985 | 0.2949495 | 0.43084392 |
| KEGG_RNA_POLYMERASE | 29 | -0.3858503 | -1.1108137 | 0.34747475 | 0.4343823 |
| KEGG_BASE_EXCISION_REPAIR | 35 | -0.3788956 | -1.1094239 | 0.35829958 | 0.42411005 |
| KEGG_PROXIMAL_TUBULE_BICARBONATE_RECLAMATION | 23 | -0.36133233 | -1.0973355 | 0.31666666 | 0.42892373 |
| KEGG_PORPHYRIN_AND_CHLOROPHYLL_METABOLISM | 41 | -0.32759324 | -1.0973283 | 0.32024795 | 0.41764796 |
| KEGG_HISTIDINE_METABOLISM | 29 | -0.33740947 | -1.0896862 | 0.33140656 | 0.41675606 |
| KEGG_TYROSINE_METABOLISM | 42 | -0.32243046 | -1.0862725 | 0.33464566 | 0.41074142 |
| KEGG_INOSITOL_PHOSPHATE_METABOLISM | 54 | -0.29598042 | -1.0630538 | 0.3501006 | 0.43042842 |
| KEGG_BETA_ALANINE_METABOLISM | 22 | -0.3356741 | -1.0540819 | 0.37575758 | 0.43093747 |
| KEGG_VASOPRESSIN_REGULATED_WATER_REABSORPTION | 44 | -0.30315197 | -1.0152049 | 0.4027505 | 0.4732842 |
| KEGG_UBIQUITIN_MEDIATED_PROTEOLYSIS | 135 | -0.26585183 | -1.0110018 | 0.41751528 | 0.46838728 |
| KEGG_BIOSYNTHESIS_OF_UNSATURATED_FATTY_ACIDS | 22 | -0.3538422 | -1.0033997 | 0.46893787 | 0.46894723 |
| KEGG_REGULATION_OF_AUTOPHAGY | 35 | -0.29270598 | -0.96554464 | 0.49593496 | 0.5115271 |
| KEGG_N_GLYCAN_BIOSYNTHESIS | 46 | -0.30604145 | -0.96508753 | 0.48 | 0.5011776 |
| KEGG_PHENYLALANINE_METABOLISM | 18 | -0.33249772 | -0.9551726 | 0.52705413 | 0.5047533 |
| KEGG_ASCORBATE_AND_ALDARATE_METABOLISM | 25 | -0.32051027 | -0.9157955 | 0.5400411 | 0.5493842 |
| KEGG_NITROGEN_METABOLISM | 23 | -0.28849152 | -0.90887374 | 0.5607287 | 0.54811954 |
| KEGG_CYSTEINE_AND_METHIONINE_METABOLISM | 34 | -0.2707751 | -0.88207567 | 0.5925926 | 0.57527584 |
| KEGG_HOMOLOGOUS_RECOMBINATION | 28 | -0.2555209 | -0.69824255 | 0.78498983 | 0.829885 |
| KEGG_OLFACTORY_TRANSDUCTION | 387 | -0.15787192 | -0.5269783 | 0.9882353 | 0.968824 |
